# Phosphorylation in the *Plasmodium falciparum* Proteome: A Meta-Analysis of Publicly Available Data Sets

**DOI:** 10.1021/acs.jproteome.4c00418

**Published:** 2024-10-30

**Authors:** Oscar J. M. Camacho, Kerry A. Ramsbottom, Ananth Prakash, Zhi Sun, Yasset Perez Riverol, Emily Bowler-Barnett, Maria Martin, Jun Fan, Eric W. Deutsch, Juan Antonio Vizcaíno, Andrew R. Jones

**Affiliations:** † Institute of Systems, Molecular and Integrative Biology, University of Liverpool, Liverpool L69 7BE, United Kingdom; ‡ European Molecular Biology Laboratory, EMBL-European Bioinformatics Institute (EMBL-EBI), Hinxton, Cambridge CB10 1SD, United Kingdom; § Institute for Systems Biology, Seattle, Washington 98109, United States

**Keywords:** phosphoproteomics, cell signaling, meta-analysis, bioinformatics, *Plasmodium falciparum*, malaria

## Abstract

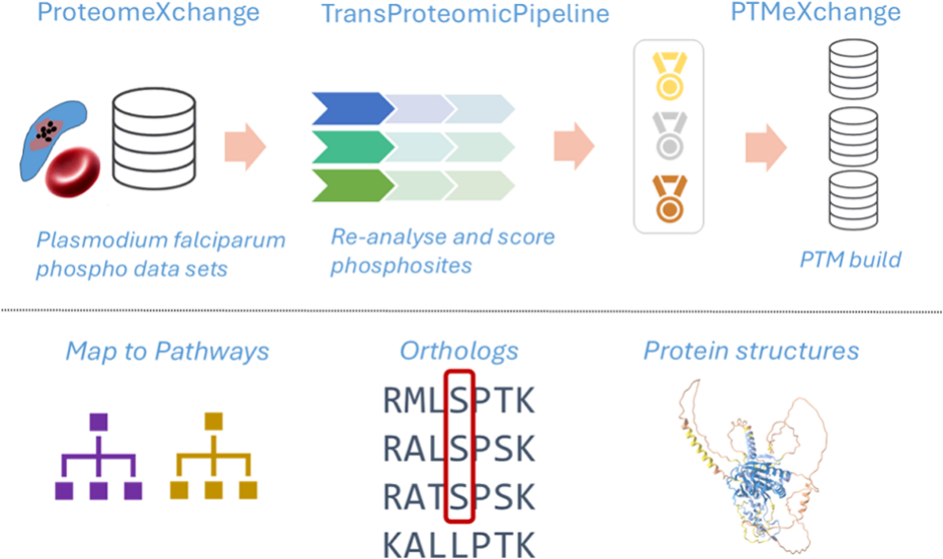

Malaria is a deadly disease caused by Apicomplexan parasites of the *Plasmodium* genus. Several species of the *Plasmodium* genus are known to be infectious to humans, of which *P. falciparum* is the most virulent. Post-translational modifications (PTMs) of proteins coordinate cell signaling and hence regulate many biological processes in *P. falciparum* homeostasis and host infection, of which the most highly studied is phosphorylation. Phosphosites on proteins can be identified by tandem mass spectrometry (MS) performed on enriched samples (phosphoproteomics), followed by downstream computational analyses. We have performed a large-scale meta-analysis of 11 publicly available phosphoproteomics data sets to build a comprehensive atlas of phosphosites in the *P. falciparum* proteome, using robust pipelines aimed at strict control of false identifications. We identified a total of 26,609 phosphorylated sites on *P. falciparum* proteins, split across three categories of data reliability (gold/silver/bronze). We identified significant sequence motifs, likely indicative of different groups of kinases responsible for different groups of phosphosites. Conservation analysis identified clusters of phosphoproteins that are highly conserved and others that are evolving faster within the *Plasmodium* genus, and implicated in different pathways. We were also able to identify over 180,000 phosphosites within *Plasmodium* species beyond *falciparum*, based on orthologue mapping. We also explored the structural context of phosphosites, identifying a strong enrichment for phosphosites on fast-evolving (low conservation) intrinsically disordered regions (IDRs) of proteins. In other species, IDRs have been shown to have an important role in modulating protein–protein interactions, particularly in signaling, and thus warranting further study for their roles in host–pathogen interactions. All data have been made available via UniProtKB, PRIDE, and PeptideAtlas, with visualization interfaces for exploring phosphosites in the context of other data on *Plasmodium* proteins.

## Introduction

Malaria remains a major global health burden, with 247 million cases worldwide in 2021. In the same year, the World Health Organization (WHO) estimated that 619,000 people died from the disease. Most malaria cases (95%) and deaths (96%) occurred in Sub-Saharan Africa. Malaria is caused by apicomplexan parasites of the *Plasmodium* genus. Of the approximately 156 named *Plasmodium* species, only five have been found to infect humans: *P. falciparum*, *P. vivax*, *P. ovale*, *P. malariae*, and *P. knowlesi*. *Plasmodium* is transmitted from one human to another by female *Anopheles* mosquitos with the exception of *P. knowlesi*, which is believed to be zoonotic i.e., transmission happens from macaques to humans in southeast Asia, where macaques have been previously infected by *Anopheles* mosquitos.^1^ Most severe cases and deaths from malaria are caused by *P. falciparum* infections, endemic to Sub-Saharan Africa.

The *P. falciparum* life cycle requires two hosts, an *Anopheles* mosquito (around 40 species of *Anopheles* can transmit *P. falciparum*(2)) and a human host. Extracellular sporozoites are transmitted to human dermal tissue during a blood meal. Once they reach the liver, they replicate and develop into merozoites and get released into the peripheral blood. There, merozoites break into erythrocytes and replicate, causing most malaria symptoms. A small proportion of parasites will develop into gametocytes with sexual attributes, which, closing the cycle, are transmitted back to mosquitoes where sporozoites are formed from gametocytes.^3^

Post-translational modifications (PTMs) of proteins are critically important, as they can act as molecular switches. PTMs, and specifically phosphorylation have been shown to dynamically change, for example, between extra and intraerythrocytic life-cycle stages suggesting protein sets and pathways with roles in cell invasion.^4^ Across stages in the *Plasmodium* intraerythrocytic asexual cycle, a study has reported changes in abundance, defined as peak changes of at least 1.5-fold between stages, for 34% of identified proteins and 75% of phosphorylation sites.^5^ Interruption and obstruction of interactions among these proteins could constitute efficient treatments against malaria.

All stages in the *Plasmodium* life cycle have the potential to generate targets for vaccines. For example, transmission-blocking vaccines preventing mosquito infection or interfering in *Plasmodium* sexual stages; pre-erythrocytic stage by disabling the ability of merozoites to reach the human liver or replicate there; or targeting interactions at the blood stage by suppressing their ability to enter erythrocytes or replicate.^6^ The most advanced vaccine (RTS,S), targets the *P. falciparum* circumsporozoite surface protein (PfCSP).^7−9^ Artemisinin-based combination treatments (ACTs) have proved effective for treating *P. falciparum* malaria^10,11^ but increasing *Plasmodium* drug resistance has been observed, highlighting the importance of the development of new drugs.^12^

There are several useful online resources to support research in *Plasmodium*. PlasmoDB^13,14^ stands out, it is part of the Eukaryotic Pathogen, Vector and Host Informatics Resources^15^ (VEuPathDB), which has been running since 2004, collating genome, functional genomic and phenotypic data sets for multiple *Plasmodium* species. PlasmoDB hosts 20 *Plasmodium falciparum* annotated genomes, including the canonical reference P. *falciparum* clone 3D7.^16^ A search of the *P. falciparum* proteome in the UniProt Knowledgebase (UniProtKB),^17^ the world’s most popular protein knowledge base, returns 18 proteomes linked to different isolates of countries of origin, which are mostly well synchronized with PlasmoDB.

Tandem mass spectrometry (MS/MS) is most often used in large-scale phosphosite identification and localization studies.^18^ Protein samples are purified and enzymatically digested, typically using trypsin. Samples are then enriched for phosphorylated peptides using reagents such as TiO2, or other metal ions that promote phosphate binding. Then liquid chromatography is used to separate peptides that are subsequently fragmented and analyzed by MS/MS. Results from MS analyses can then be compared against protein sequence databases, with and without the mass shift for phosphorylation, via one of the many available search algorithms.^19,20^ Algorithms provide identification of peptides and localization of PTM sites in those peptides with scores aiming to reflect the level of confidence that those identifications are correct. Score thresholding is used to select a subset of what is expected to be the most confident findings. However, score thresholding does not provide information about the global false discovery rate (FDR) of peptides or the global false localization rate (FLR) of the phosphosites within those peptides. The absence of objective calculations of FLR in phosphosite localization studies hinders comparisons among studies, as it is not possible to establish a common quality threshold among results from different studies. To overcome this problem, we have recently published an approach that allows estimation of global site level FLR, by including a decoy amino acid, specifically Alanine, for phosphorylation searches (which cannot be modified) as a search parameter to compete against target sites (S, T or Y), i.e., the pASTY method.^21^ An important benefit of pASTY searches is that it allows the combination of results from multiple studies as FLR can provide objective comparable thresholds.^22^

For *P. falciparum*, PlasmoDB provides information on 16,118 phosphorylation sites, although phosphorylation sites have been loaded from multiple publications over the last 10 years. In our previous work examining databases containing human phosphosites, we estimated that there is a high proportion of false positive sites recorded, due to historically inadequate statistics associated with the detection of sites by MS^23^ and, prior to,^22^ lack of methods for calculating adequate statistics for controlling the FLR across studies.

In this work, we aim to provide a high-quality mapping of *P. falciparum* phosphosites via a large-scale reanalysis of public phospho-enriched studies, underpinned by robust analysis pipelines enabling meta-analyses with FLR control within and across studies’ results. This analysis is part of the “PTMeXchange” initiative, which is reanalyzing phospho-enriched data sets and depositing results into the proteomics resources PRIDE,^24^ PeptideAtlas,^25^ and UniProtKB. Results from downstream analysis in the most confident phosphosites are also reported in this manuscript, including analysis motifs centered on phosphosites and pathway enrichment analysis for these motifs. We examine phosphorylation site conservation between our reference isolate 3D7 (*P. falciparum*) and other species of the *Plasmodium* genus as well as investigating the structure and disordered regions of phosphoproteins.

## Methods

### Data Selection

The ProteomeXchange Central database^26^ was used to identify suitable *P. falciparum* phosphoproteomics data sets, via the PRIDE repository.^27^ Overall 11 were deemed suitable for phosphosite (localization) reanalysis, based on inclusion criteria that data sets were generated by data-dependent acquisition (DDA) methods, “high-quality” (i.e., likely to deliver >1000 phosphosites based on analysis of source manuscripts), raw data were available and readable using open source tools: PXD000070,^28^ PXD001684,^4^ PXD002266,^29^ PXD005207,^30^ PXD009157,^31^ PXD009465,^32^ PXD012143,^33^ PXD015093,^34^ PXD015833,^35^ PXD020381,^36^ and PXD026474.^37^ These 11 data sets consist of both labeled (TMT or iTRAQ) and unlabeled MS data sets. Most of the studies focus on the blood stage in *P. falciparum* life cycle, with only one study (PXD026474) including gametocytes. There were no data available for the liver stage of the parasite, possibly due to challenges with the development of liver models. A brief description of the studies’ objectives extracted from the abstracts of their publications, *P. falciparum* stages included in the analysis and enrichment details are shown in Table 1 and more details about the studies’ objectives and biological samples in Supplementary Table 1.

**Table 1:** Summary Results and Sample Used for Each Study Are Included in This Analysis

study ID	sample	enrichment method	refs
PXD000070	*Parasites cultured from infected erythrocytes*	*IMAC*	(28)
PXD001684	*Merozoites*	*TiO2*	(4)
PXD002266	*Late-stage schizonts*	*TiO2*	(29)
PXD005207	*Late-stage schizonts*	*TiO2*	(30)
PXD009157	*Fully segmented schizonts*	*TiO2*	(31)
PXD009465	Schizont/Ring Stages (time course)	IMAC	(32)
PXD012143	Schizont/Ring	SMOAC (TiO2 and Fe-NTA)	(33)
PXD015093	*Merozoites*	TiO_2_/ZrO_2_	(34)
PXD015833	*Asexual red blood cell stages*	TiO_2_	(35)
PXD020381	*Schizont*	TiO2 and Fe-NTA	(36)
PXD026474	*Gametocytes*	Ti-IMAC/Zr-IMAC	(37)

### Phosphosite Localization

The search database was created from sequences derived from the PlasmoDB 51 release of *Plasmodium falciparum* 3D7 and humans. The human sequences were obtained from the Level 1 PeptideAtlas Tiered Human Integrated Search Proteome,^38^ containing core isoforms from neXtProt^39^ (2020 build). A fasta file was created combining these sequences with cRAP contaminant sequences (https://www.thegpm.org/crap/) plus decoy sequences. One decoy sequence was generated for each protein and contaminant sequence using the de Bruijn method (with *k* = 2).^40^

Data analysis was performed using the Trans-Proteomic Pipeline (TPP),^19^ including Comet^41^ search engine for individual data sets, followed by PeptideProphet,^42^ iProphet,^43^ and PTMProphet;^44^ these were grouped within each study according to the labeling and MS characteristics, as they determine search parameters. The files were searched with several variable modifications depending on the study design, and phosphorylation of ASTY. Alanine was included in searches as a decoy amino acid to enable the estimation of the FLR as explained by Ramsbottom et al.^21^ Other variable modifications included in the analyses were as follows: Oxidation in MWH (HYDR), protein N-terminal acetylation or at K (ACET), ammonia loss at peptide n-terminal QC (PYRO), pyroglu at peptide n-terminal E (DHB), deamidation at NQ (DEAM), formylation of the N-terminus (FORM) and fixed modification Carbamidomethylation (C), as noted in Supplementary Table 1. iTRAQ4plex, TMT6, or TMT10 labeling were included in searches as appropriate. As search parameters, a maximum of 2 missed cleavages were specified and the maximum number of 5 different modifications per peptide were allowed.

### Postprocessing Search Results

The data files obtained from searching with TPP were processed by custom Python scripts (https://github.com/PGB-LIV/mzidFLR) and analyzed following a previously published pipeline.^22^ First, at the peptide-spectrum match (PSM) level, the FDR was calculated based on decoy sequences, and the PSMs were filtered to 1% FDR. Data from the most confident PSMs (1% FDR) were transformed to give a site localization score for each phosphosite found on each PSM, removing matches to decoy PSMs and contaminant entries. A final site-based PSM score was obtained by multiplying the peptide identification probability by the site localization probability and adjusted considering the number of occasions each site was observed, phosphorylated and not phosphorylated, in the data set.^22^

Once the final probability at PSM level was calculated, the data was collapsed to the “peptidoform-site” level by taking the maximum score among all matches for each phosphosite on a given peptidoform within each analysis and study. FLR was calculated by ordering all peptidoform sites by their final score. At this stage, matches to *P. falciparum* and human matches were separated according to their respective protein matches.

A further categorization at the protein-site level was achieved by using FLR calculations for each study. This gold–silver–bronze (GSB) categorization allows additional grading for our confidence in the findings among the most confident sites. A simple exclusive criterion was applied: Gold sites observed at 2 or more independent studies at 1% FLR; silver sites observed in only 1 data set at <1% FLR; bronze: all other sites at 5% FLR.

Note that within each study, if there was more than 1 peptidoform site as evidence for a protein site, the one with the lowest FLR was used for GSB categorization.

### Downstream Analysis

#### Summarizing Outcomes

Peptidoforms mapping to only human sequences were filtered from the results. A frequency table was produced for PSMs at 1% FDR level, PSM sites at 1% FDR level, number of phosphosites at the PSM level, peptidoform-site level, and protein-site level at 1% and 5% FLR.

GSB phosphosite counts were generated considering that some of the sequences and therefore sites could map to different proteins or sites within their respective proteome. The count of phosphosites in sequences mapping to a single site (unique) and to more than one site was also calculated.

All analyses and graphical output were obtained using the R programming language (version 4.2.1) or above via RStudio (2022.02.3 Build 492).

### Motif and Pathway Enrichment Analysis

All *P. falciparum* data sets were investigated using motif and pathway enrichment analysis. For this, we used all STY phosphosites at 5% FLR, combining all 11 studies and removing matches to the decoy amino acid Alanine. 15mer peptides centered on each phosphosite were generated to investigate motifs around these sites. They were compared against 15mer “background” sequences generated using any STY in these data sets, whether they were phosphorylated or not, at its center (position 0). Statistically significant enriched motifs surrounding phosphosites were identified using the R package *rmotifx*(45) Results were thresholded via *p*-value < 1 × 10^–9^ for pS and *p* < 1 × 10^–6^ for pT and pY respectively; and a minimum of 20 sequences per motif.

The R package clusterProfiler^46^ was used to carry out a pathway enrichment analysis considering those phosphoproteins containing specific enriched motifs. All other proteins in the search database were used as the background for this analysis. A heatmap on the adjusted p-values of a subset of motifs with fold-change enrichment (versus the background) > 4 was produced representing motifs against GO (Gene Ontology) terms.

### Conservation Analysis

*P. falciparum* isolate 3D7 identified phosphosites were investigated regarding 22 species of the *Plasmodium* genus. The 22 species of *Plasmodium* were: *P. gorilla clade G1*(PPRFG01), *P. reichenowi* (PRCDC), *P blacklocki* G01 (PBLACG01), *P. billcollinsi G01* (PBILCG01), *P. adleri G01* (PADL01), *P. gaboni* (PGSY75), *P. malariae* (PmUG01), *P. brasilianum strain Bolivian I* (MKS88), *P. ovale* (PocGH01), *P. relictum* (PRELSG), *P. gallinaceum* (PGAL8A), *P. chabaudi AS* (PCHAS), *P. vinckei vinckei CY* (PVVCY), *P. yoelii 17X* (PY17X), *P. berghei ANKA* (PBANKA), *P. cynomolgi* (PcyM), *P. vivax* (PVP01), *P. knowlesi* (PKNH), *P. coatneyi Hackeri* (PCOAH), *P. fragile strain nilgiri* (AK88), *P.inui San Antonio 1* (C922), and *P. vivax-like* (PVL). They were compared against *P. falciparum* 3D7 in terms of sequence conservation. Protein sequences were downloaded from PlasmoDB and mapped to the identified phosphosites using the “syntenic ortholog” mappings stored in PlasmoDB, generated by OrthoMCL,^47^ followed by protein-level multiple sequence alignment with muscle 5.1 running on Linux.^48^

Matched residues in orthologs were labeled as 1 while not matching sites, due to having a different amino acid in that position in the ortholog, a gap in the sequence, or simply not having an ortholog for a protein, were labeled as 0. For each species and phosphoprotein considered, a mean conservation score was calculated for the proportion of phosphosites within each protein, which were conserved within the orthologue from that particular species. Heatmaps were created based on the mean conservation score. Protein clusters were identified and investigated for enrichment analysis using clusterProfiler.

Further functional enrichment analyses were performed comparing conservation results from human transmissible species *P. malariae*, *P. ovale and P. vivax* to the other 17 animal species *(*excluding *P. knowlesi* and *P. vivax-like* due to their zoonotic character). The subset of proteins included in the enrichment analysis were those proteins fully conserved for the three human transmissible species and those not fully conserved across all of the animal species.

Conservation of phosphosites within *Plasmodium falciparum* was calculated using single nucleotide polymorphism (SNP) data stored in PlasmoDB (public search strategy: https://plasmodb.org/plasmo/app/workspace/strategies/import/b4d2489952494797), using variants from 115 aligned genomes from two unbiased SNP data set https://plasmodb.org/plasmo/app/record/dataset/DS_9a7f849906 (“*P. falciparum* 100 Genomes project”), and https://plasmodb.org/plasmo/app/record/dataset/DS_d1c8287de9.^49^ SNP sites were downloaded and nonsynonymous variants, altering the amino acid sequence, were matched to the phosphosite positions in proteins (using Python 3.9 code). Site conservation was estimated using the major allele frequency, calculated by the PlasmoDB.

We next explored the average disorder of amino acid positions within proteins using metapredict v2^50^ running on Linux, comparing and correlating disorder scores with site classifications. To visualize examples of phosphosites with different conservation values, we used iCn3D,^51^ and generated URLs encoding phosphosite conservation scores using custom Python code. The list of *P. falciparum* 3D7 kinases was generated by searching for proteins annotated with Interpro or Pfam “protein kinase” domains in PlasmoDB: https://plasmodb.org/plasmo/app/workspace/strategies/import/6a11331d6eea4d20.

Phosphosite data for *Plasmodium falciparum* 3D7 was loaded into UniProtKB, by taking all mapping all peptides carrying GSB sites to proteins in UniProtKB *P. falciparum* 3D7 proteome (https://www.UniProt.org/proteomes/UP000001450), assuming tryptic cleavage.

To facilitate data reuse, for example in data-independent acquisition approaches, spectral libraries were created for all data sets using SpectraST,^52^ divided by peptide fragmentation mode (HCD, CID or ETD) and by labeling type (TMT, iTRAQ or label-free–all HCD), making six libraries in total, based on the combination of data sets in the build. The SpectraST library-building process combines replicate fragment ion spectra from the same precursor ion, weighted by their signal-to-noise ratio, into a high-quality, denoised consensus spectrum.

## Results

### Phosphosite Identification

**Figure 1:**
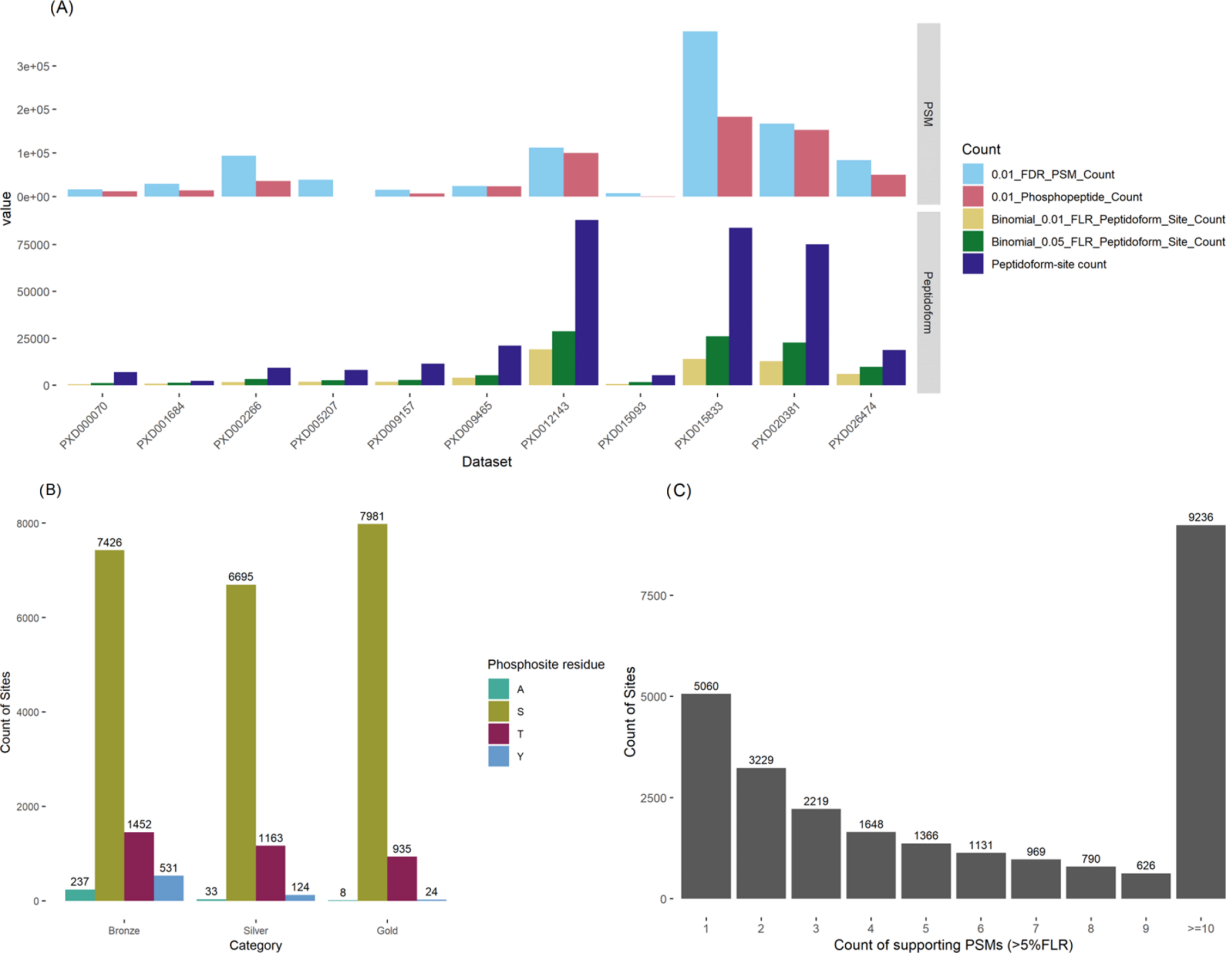
A: Counts of phosphosites before and after (peptidoform) collapse for removing redundancy at 1% and 5% FLR levels, per data set. B: The count of protein sites classified as gold–silver–bronze for P. falciparum, sites colored by phosphorylated amino acid. This includes all potential locations for the identified sites when peptides match more than one location or protein and decoy matches to Alanine. C: Counts of phosphosites with one or more supporting PSMs, at the 5% FLR threshold.

As described in the methods, we first curated all publicly available phosphoproteome MS data sets and applied inclusion criteria to select 11 data sets for reanalysis. We then applied our reanalysis pipeline consisting of sequence database search, and statistical analysis at the peptide and PTM-site level per data set to generate robust groups of phosphosites for each data set. The counts of peptide-spectrum matches (PSMs), and PSM sites passing the 1% FDR threshold are displayed in Table 2 as well as the number of overall PSM sites at 1% and 5% FLR. Next, data were collapsed to peptidoform-site level i.e., removing redundancy caused by the common occurrence of multiple PSMs identifying the same peptidoform. A peptidoform is defined as a unique sequence of amino acids with specific modifications. For example, two identical peptide sequences but with modifications at different positions in the sequence are different peptidoforms. Table 2 also displays the number of peptidoform sites and protein sites (accepting the mapping from a peptide sequence to all proteins it can be found in, assuming tryptic cleavage) at 1% and 5% FLR. Note that FLR threshold counts only considered PSMs that passed 1% FDR. The data is unequally distributed among the 11 studies, with four studies (PXD012143, PXD015833, PXD020381, and PXD026474) contributing significantly more to the overall number of sites at every stage of the analysis. In fact, nearly 70% of all *P. falciparum* protein sites at 5% FLR come from these four studies. Figure 1A shows the summary of PSM counts and site counts, before and after removing redundancy, for each data set at 1% and 5% FLR thresholds.

**Table 2:** P. falciparum PSM Count at 1% FDR, PSM Site Count at 1% and 5% FLR, Peptidoform Site Count at 1% and 5% FLR and Protein Site Count at 1% and 5% FLR

	1% FDR PSM	1% FDR PSM site	1% FLR PSM Site	5% FLR PSM-Site	1% FLR Peptidoform site	5% FLR Peptidoform site	1% FLR Protein site	5% FLR Protein site
PXD000070	16,184	38,903	5100	10,390	482	1125	393	905
PXD001684	28,923	13,758	5523	11,669	901	1291	803	1141
PXD002266	93,194	41,237	9509	16,755	1609	3298	1160	2455
PXD005207	38,213	20,169	4161	7462	1820	2637	1336	1944
PXD009157	15,250	22,671	3869	6571	1794	2748	1320	2069
PXD009465	23,906	35,314	7504	10,257	3905	5382	2538	3573
PXD012143	112,204	326,827	90,514	139,019	19,132	28,713	10,753	16,048
PXD015093	7506	27,393	5064	10,889	678	1604	520	1261
PXD015833	379,174	352,661	70,265	120,056	13,921	26,134	6503	12,083
PXD020381	167,500	266,964	65,190	101,867	12,741	22,729	7423	13,266
PXD026474	83,621	98,398	37,976	63,682	5987	9825	4751	7673

Figure 1B shows the gold–silver–bronze (GSB, see Methods) quality categorization for *P. falciparum* protein sites, generated following merging of sites across different data sets. If a sequence, and therefore the sites within the sequence, matches more than one protein sequence, these sites were counted for all mapping proteins in the proteome. In total, there were 26,609 protein sites in *P. falciparum* classified as GSB (5% FLR) of which 8940 were gold (seen in 2 or more studies at <1% FLR), 7982 silver (1 study at <1% FLR), and 9409 bronze (≥1% FLR and <5% FLR), excluding decoy hits to alanine.

Within the gold set, 89.3% were pS sites, 10.5% pT and 0.2% were pY sites. While tyrosine phosphorylation can occur in *Plasmodium falciparum*, these sites likely occur at low site occupancy and require specialized protocols (e.g., antiphosphotyrosine antibodies) for their detection, which were not employed in these source data sets. All gold, silver, and bronze phosphosites can be found in Supp File 1. Figure 1C displays the PSM counts supporting sites, showing that most sites are supported by multiple PSMs. PSM counts for individual sites are also given within Supp File 1—giving a semiquantitative measure of how frequently or abundantly a given site is observed in different sample types.

### Motif and Pathway Enrichment Analysis

**Figure 2:**
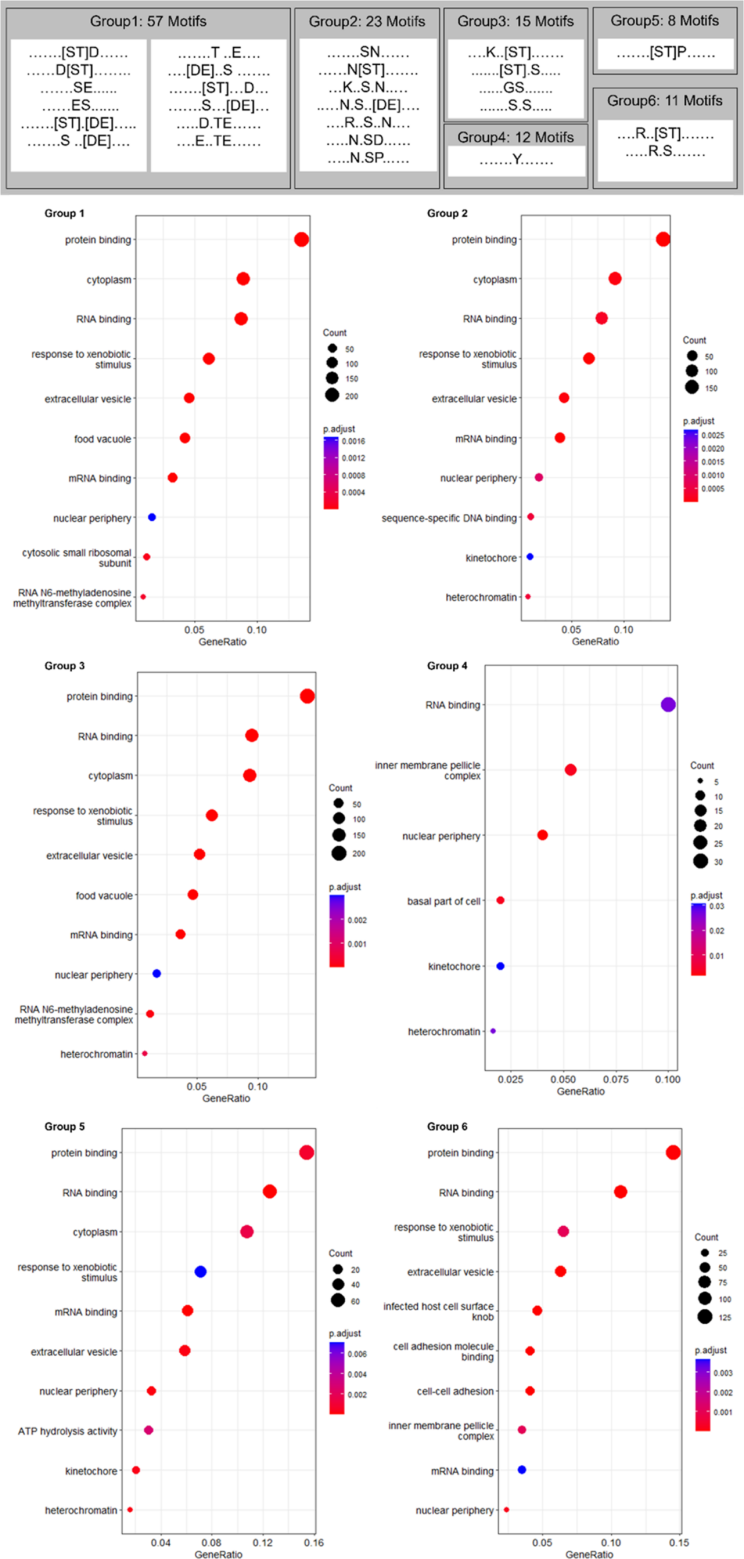
Statistically significant GO terms for motifs surrounding phosphorylated sites, grouped by similar motifs, formed by similar groups of amino acids.

We investigated if there were over-represented sequence motifs centered on phosphosites (<5% FLR). Motif analysis was performed for *P. falciparum* by comparing 15mer peptides centered on S, T, and Y phosphosites against a background of 15mer peptides centered on all STY sites, phosphorylated or not. The analysis returned 107 statistically significant motifs, of which 65, 30, and 12 were centered on S, T, and Y, respectively (Supp Figure 1 and Supp File 1). Under the hypothesis that similar motifs may indicate that proteins are regulated by the same or related kinases, we identified six groups based on the amino acids forming those motifs (Figure 2, top panel).

The most common significant motifs (group 1 in Figure 2) were those with S and T combining with E and D in different positions such: [ST]D, D[ST], [ST].[DE], [ST]···D, and [ST]N[DE], N[ST].[DE] or N.[ST] [DE]. There were also other common motifs to S and T not containing D or E like N[ST] (group 2), K..[ST] (group 3), R..[ST] (group 6). Within several groups of motifs, K was commonly found at positions −7, −6, + 6, and +7, with significant motifs for K···.DS, K···DS and SD···K, SD···.K, SN···.K. Motifs with K relatively distant to the target site may be due to a preference for particular kinases or an artifact related to tryptic cleavage enriching for lysines to be present somewhere in many detected peptides. There were only six 3-amino acid motifs with a P in the +1 position, which can be very common for other species. Many of these motifs agreed with those found by Pease et al.,^32^ which can be expected as their MS data was included in this analysis. Treeck et al.^53^ also found many of these motifs in *P. falciparum* (S[DE].E,SD.[ED], K..S.D, [KR]..S, S[DE], SN, DS).

Motifs were independently analyzed to investigate the functional enrichment of the proteins in which specific motifs were found. Summing across all motif results, 39 different GO terms were found to be statistically significant. Table 3 contains significant GO terms for motifs at least 4-fold enriched versus the background, and the full analysis for all motifs can be found in Supp File 2. It is worth noting that the RSF.D motif gave highly significant matches to several GO terms. However, this result is an unusual artifact of the protocol. *P. falciparum* has a 65-gene family, in which all members are called “PfEMP1″, with highly related protein sequences. Phosphosites are mapped to all positions where a peptidoform can be found, typically resulting in the vast majority of sites mapping to a single protein (counts for the few exceptions are shown in Figure 1D). Phosphosites identified in PfEMP1 mapped to 36 different proteins, which gives an apparently extremely significant signal under motif-GO analysis since the PfEMP1 proteins all contain the same motif and are also all mapped to the same GO terms.

**Table 3:** Statistically Significant GO Terms for the Subset of Motifs Surrounding Phosphorylated Sites^a^

ID	description	GeneRatio	BgRatio	pvalue	p.adjust	qvalue	count	motif
GO:0034399	nuclear periphery	4/52	35/3454	0.0016	0.0374	0.0367	4	.···..SD.E..K.
GO:0005515	protein binding	13/52	354/3454	0.0017	0.0374	0.0367	13	.···..SD.E..K.
GO:0045178	basal part of cell	4/144	11/3454	0.0007	0.0314	0.0302	4	.···R.NS···...
GO:0048870	cell motility	4/144	12/3454	0.0011	0.0314	0.0302	4	.···R.NS···...
GO:0009410	response to xenobiotic stimulus	14/144	145/3454	0.0024	0.0470	0.0451	14	.···R.NS···...
GO:0000792	heterochromatin	5/233	11/3454	0.0004	0.0209	0.0197	5	.···..SD.E···
GO:0005515	protein binding	40/233	354/3454	0.0005	0.0209	0.0197	40	.···..SD.E···
GO:0034399	nuclear periphery	8/233	35/3454	0.0018	0.0472	0.0446	8	.···..SD.E···
GO:0050839	cell adhesion molecule binding	30/36	54/3454	5.59 × 10^–53^	6.71 × 10^–52^	2.35 × 10^–52^	30	.···.RSF.D···
GO:0098609	cell–cell adhesion	30/36	57/3454	5.57 × 10^–52^	3.34 × 10^–51^	1.17 × 10^–51^	30	.···.RSF.D···
GO:0020030	infected host cell surface knob	30/36	61/3454	9.17 × 10^–51^	3.67 × 10^–50^	1.29 × 10^–50^	30	.···.RSF.D···
GO:0020002	host cell plasma membrane	30/36	214/3454	1.10 × 10^–31^	3.30 × 10^–31^	1.16 × 10^–31^	30	.···.RSF.D···
GO:0020013	modulation by symbiont of host erythrocyte aggregation	29/36	191/3454	2.49 × 10^–31^	5.98 × 10^–31^	2.10 × 10^–31^	29	.···.RSF.D···
GO:0020035	adhesion of symbiont to microvasculature	28/36	172/3454	7.88 × 10^–31^	1.58 × 10^–30^	5.53 × 10^–31^	28	.···.RSF.D···
GO:0020033	antigenic variation	29/36	202/3454	1.40 × 10^–30^	2.41 × 10^–30^	8.44 × 10^–31^	29	.···.RSF.D···
GO:0046789	host cell surface receptor binding	7/36	37/3454	5.95 × 10^–08^	8.93 × 10^–08^	3.13 × 10^–08^	7	.···.RSF.D···
GO:0043565	sequence-specific DNA binding	4/77	17/3454	0.0004	0.0178	0.0160	4	.···N..SP···..
GO:0009410	response to xenobiotic stimulus	10/75	145/3454	0.0009	0.0484	0.0416	10	.···K..T.D···.
GO:1903561	extracellular vesicle	7/57	91/3454	0.0006	0.0221	0.0206	7	.···E..T.E···.
GO:0042393	histone binding	2/19	12/3454	0.0018	0.0274	0.0212	2	.···..TP···E..
GO:1903561	extracellular vesicle	20/209	91/3454	2.51 × 10^–07^	1.88 × 10^–05^	1.72 × 10^–05^	20	.···R..T···...
GO:0003723	RNA binding	27/209	199/3454	4.23 × 10^–05^	0.0014	0.0013	27	.···R..T···...
GO:0020020	food vacuole	17/209	98/3454	5.83 × 10^–05^	0.0014	0.0013	17	.···R..T···...
GO:0045178	basal part of cell	5/209	11/3454	0.0002	0.0049	0.0045	5	.···R..T···...
GO:0048870	cell motility	5/209	12/3454	0.0004	0.0064	0.0059	5	.···R..T···...
GO:0005515	protein binding	35/209	354/3454	0.0018	0.0236	0.0216	35	.···R..T···...
GO:0042393	histone binding	3/30	12/3454	0.0001	0.0033	0.0028	3	.···D.TE···..
GO:0032991	protein-containing complex	2/23	14/3454	0.0036	0.0446	0.0405	2	.···..Y.SD···
GO:0006511	ubiquitin-dependent protein catabolic process	2/23	15/3454	0.0042	0.0446	0.0405	2	.···..Y.SD···
GO:0003724	RNA helicase activity	2/23	18/3454	0.0061	0.0446	0.0405	2	.···..Y.SD···
GO:0003723	RNA binding	6/20	199/3454	0.0006	0.0153	0.0147	6	.···.SYE···..

a Analyses were carried out independently for each motif and include only motifs with at least a 4-fold change over the background.

We next explored the enriched GO terms for each group of phosphorylation motifs (Figure 2, bottom panel). Our analysis showed that besides general agreement on high-level functional terms such as “protein binding”, “cytoplasm” or “RNA binding” there were also group-specific GO terms pointing to some functional specificity among motif groups. There was almost complete agreement in significant terms between groups 1 to 3 which could suggest there is overlap in the signaling cascades with regards to downstream effects, or the method may not be sensitive enough to draw more specific terms allowing differentiation. Groups 4 to 6 returned more diverse GO terms, with group 6 results biased by the PfEMP1 protein family, as previously explained.

**Figure 3:**
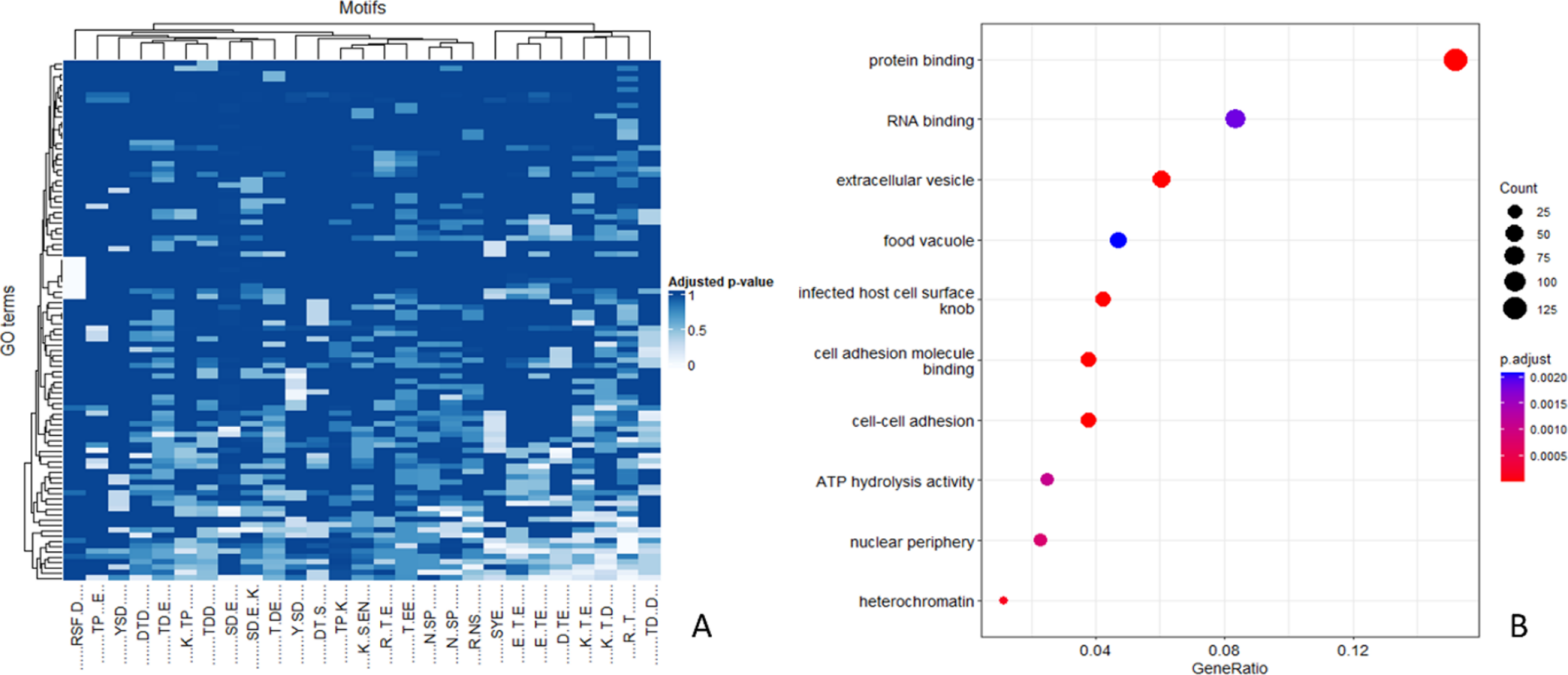
(A) Heatmap of the adjusted p-values resulting from an enrichment analysis of the proteins where phosphorylation motifs (on the *x*-axis) were found (only motifs with fold change over background above 4 are considered). (B) Significant GO Terms (p-adjusted ≤0.05) for the genes in which motifs with fold change over background above 4 could be found.

As an alternative and more objective approach for grouping motifs, we took a subset of motifs with a 4-fold change over the background, i.e., the most strongly over-represented motifs, and performed an enrichment analysis for all *P. falciparum* phosphoproteins in which those phosphorylation motifs can be found. Figure 3A shows a heatmap of the p-values for the GO terms associated with proteins containing these phosphorylation motifs. These motifs did not seem to form strong clusters according to these terms, although some small groups (similar pairs clustering together) could be observed. In a few cases, there was some overlap in protein sets carrying motifs, for example, SD.E and SD.E..K, the latter being matched to a subset of the proteins matched by the former. However, the motifs K..T.E and K..T.D are mutually exclusive, yet proteins carrying these phosphosite motifs can be seen to be acting in many of the same signaling pathways. Other examples of “pairing” on the dendrogram of mutually exclusive motifs are N.SP and N..SP, and E..T.E and E···TE. It is possible that these pairs of phospho-motifs are recognized by the same kinase or that there are closely related kinases within the same family that are involved within the same types of downstream pathways. Another “pair” of phospho-motifs is K..TP and TDD; this latter example is surprising since it would typically be assumed that S/TP and S/TD phosphosites are regulated by different kinase families. It is possible that there is cross-talk between two kinases, although the evidence here is not sufficient to form any strong conclusions.

Therefore, in general, the heatmap suggests some differences in functionality between proteins containing different phosphorylation motifs. However, when analyzed as a group, 10 GO terms summarized the majority of the functional processes for the subset of genes where these phosphorylated motifs were found (Figure 3B).

The *P. falciparum* proteome has around 90 protein kinases; 89 are annotated in PlasmoDB with the InterPro protein kinase domain term. An analysis by Adderley et al.^54^ identified 98 protein kinases in isolate 3D7, by including keyword searching in addition to InterPro domain searching. From their list of kinases, there are several sequences annotated as pseudokinases or having kinase-like domains (PF3D7_0424700, PF3D7_0708300, PF3D7_0724000, PF3D7_0823000, PF3D7_1106800, PF3D7_1321100, PF3D7_1428500) and two pseudogenes (PF3D7_0731400 and PF3D7_1476400). Adderley et al.^54^ classified these kinases into families, using an HMM (Hidden Markov Models)-based technique. Supp Table 2 shows the counts of 3D7 kinases classified into families, with additional notes on potential kinase motifs for these families based upon information on kinase motifs found in humans from,^55^ with the caveat that we cannot be sure that even if orthologous kinases exist between humans and *P. falciparum* that they recognize the same motifs. Accurate prediction of kinase-substrate relationships is not straightforward without high-quality experimental data, which is lacking in *P. falciparum.* FIKK kinases are unique to Apicomplexa,^56^ and seem to have an important role in host invasion (such as phosphorylation of erythrocyte proteins), but otherwise little is known about the motifs for their targets. The publication associated with data set PXD015833 specifically investigated substrate specificity and concluded that some of the FIKK family have a preference for a basic motif near the pS/pT site with arginine enriched in the minus position (group 6 in our analysis).^35^ We could speculate that the motifs groups identified in Figure 2 are driven by different kinase groups in Supp. Table 2, but without new experimental data, robust conclusions are not possible. This is an area that requires further work.

### Conservation Analysis

**Figure 4:**
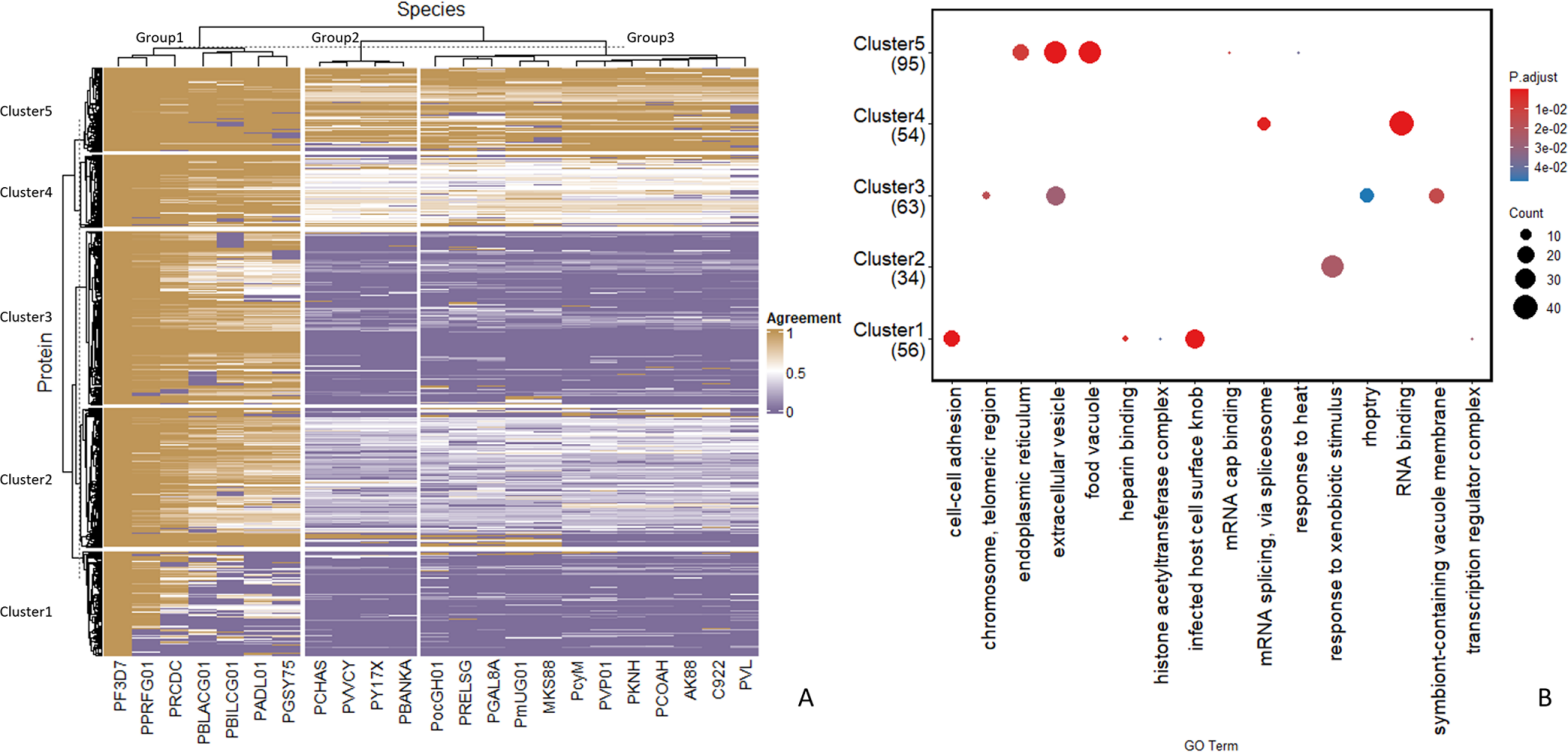
(A) Heatmap of the agreement in sequence conservation between 23 species of Plasmodium: P. gorilla clade G1(PPRFG01), P. reichenowi (PRCDC), P. blacklocki G01 (PBLACG01), P. billcollinsi G01 (PBILCG01), P. adleri G01 (PADL01), P. gaboni (PGSY75), P. malariae (PmUG01), P. brasilianum strain Bolivian I (MKS88), P. ovale (PocGH01), P. relictum (PRELSG), P. gallinaceum (PGAL8A), P. chabaudi AS (PCHAS), P. vinckei vinckei CY (PVVCY), P. yoelii 17X (PY17X), P. berghei ANKA (PBANKA), P. cynomolgi (PcyM), P. vivax (PVP01), P. knowlesi (PKNH), P. coatneyi Hackeri (PCOAH), P. fragile strain nilgiri (AK88), P. inui San Antonio 1 (C922), P. vivax-like (PVL) and the reference P. falciparum (PF3D7). The heatmap displays the mean conservation (agreement) of phosphosites per protein with three “groups” of species and five “clusters” for proteins. (B) Pathway enrichment analysis for the genes found in each cluster with significant GO terms in *y*-axis and clusters in the *x*-axis; the number of genes where those terms were found are in parentheses under each cluster label.

Based on unique phosphorylated protein sites at 5% FLR in *P. falciparum* 3D7, we investigated the existence of their orthologs in other species of *Plasmodium*. Conservation for each site, defined as the proportion of species containing the same amino acid at that position in the multiple sequence alignment, can be found as supplementary files (Supp File 3.). We generated a heatmap (Figure 4) representing the average agreement among sites within each protein. If the site was conserved with respect to the reference 3D7 it was labeled as 1 and 0 otherwise. For each species, a nonconserved site could be the result of having a different amino acid with respect to the reference phosphorylation position, there could be a gap in the protein or the orthologue was not found for that species. Then, within each protein and species, the average (proportion of sites conserved) was calculated. In this way, proteins without any sites conserved or when the protein is not found in the species have score = 0 and when all phosphosites are conserved, the score = 1. Changes in a site between Ser, Thr, or Try were considered not conserved, although scores were also calculated allowing for S ↔ T substitutions as “conserved” (not disruptive to S/T phosphorylation), as shown in Supp File 3.

Mapping across species returned 26,316 sites belonging to 2,890 proteins, where only 1 mapping occurrence was used per phosphorylated protein site, i.e., when phosphorylated peptides could match to more than one protein (or very rarely multiple positions within one protein), only one match was used per peptide. Of those 2890 proteins, only 108 proteins contained all phosphosites that were completely conserved across all species considered. Hence, the heatmap in Figure 4A is formed of the 2782 proteins for which there were differences between two or more orthologous proteins in the conservation of their identified phosphosites.

The dendrogram suggests three main groups for the *Plasmodium* species on the *x*-axis, with PPRFG01 (*Plasmodium sp.* gorilla clade 1) closest to the *P. falciparum* reference PF3D7 (Figure 4A). This group (group 1) containing *P. falciparum* has six more species in the cluster, none of which (beyond 3D7) is transmissible to humans. The two other groups are formed of 4 and 12 species. All other species transmissible to humans are included in the largest group (group 3). On the *y*-axis, five clusters of proteins were formed and subsequently analyzed separately with clusterProfiler for enrichment analysis, to determine if there were different biological processes associated with phosphosites of different conservation patterns, which might be indicative of those under different selective pressures. The analysis yielded 16 statistically significant GO terms (Figure 4B).

For Cluster 5 (proteins containing phosphosites mostly conserved across the genus), the most significant GO terms per cluster were: “food vacuole” (GO:0020020), ”extracellular vesicle” (GO:1903561) and “endoplasmic reticulum” (ER) (GO:0005783) indicating conserved signaling mechanisms related to the infection of host cells. The ER in Apicomplexa is known to be involved in the processing of effector proteins before translocation to host cells.^57^ The “food vacuole” is a GO term mostly used in the annotation of Apicomplexa proteins related to the digestion of the host cell cytoplasm. Cluster 4 contains sites that are highly conserved in Group 1 species and around 50% conserved in Group 2 and 3 species. Cluster 4 is annotated to be enriched with GO terms related to RNA binding and splicing. This result is somewhat surprising since one would assume that mechanisms related to transcription would be very highly conserved. Cluster 3 are highly conserved in Group 1 species but has low conservation in groups 2 and 3, with enrichment for GO terms related to symbiont-containing vacuole membrane, rhoptry, and extracellular vesicle; we could speculate that this may be indicative of cell signaling related to host cell invasion, and evolving faster than Cluster 5 proteins, to evade host immune responses. Cluster 2 proteins have highly conserved phosphosites in Group 1 proteins but with average conservation between those in Cluster 2 and Cluster 4 for the other groups. Only one GO term is enriched for “response to xenobiotic stimulus” (GO:0009410)—a term related to *Plasmodium*’s ability to respond to small molecules from the host (and proteins implicated in drug resistance). Cluster 1 contained proteins with the least conserved phosphosites and had the strongest enrichment (by p-value and count of mapped terms) for “infected host cell surface knob” (GO:0020030) and “cell–cell adhesion” (GO:0098609). Term GO:0020030 is a commonly used gene annotation in *Plasmodium*, related to the protrusions in the membrane of an infected erythrocyte. These proteins are potentially under pressure to evade host immune responses, thus evolving much faster (and changing their cell signaling mechanisms). Data for this analysis including the clusters can be found in Supp. File 4.

We next implemented a “strict” phosphosite matching process, for the purposes of providing highly likely phosphosites for all *Plasmodium* species aligned, requiring that the phosphosite amino acid matched between *Plasmodium falciparum* 3D7 and the target species (allowing for S ↔ T substitutions) and requiring the +1 residue was also matched (as the most important position for phosphorylation motifs). This gives an additional candidate set of 183,134 phosphosites, identified across the *Plasmodium* genus, based on orthologue mapping (Supp. File 5). The multiple sequence alignments for all phosphoproteins are provided in a compressed folder (Supp. File 6).

**Figure 5:**
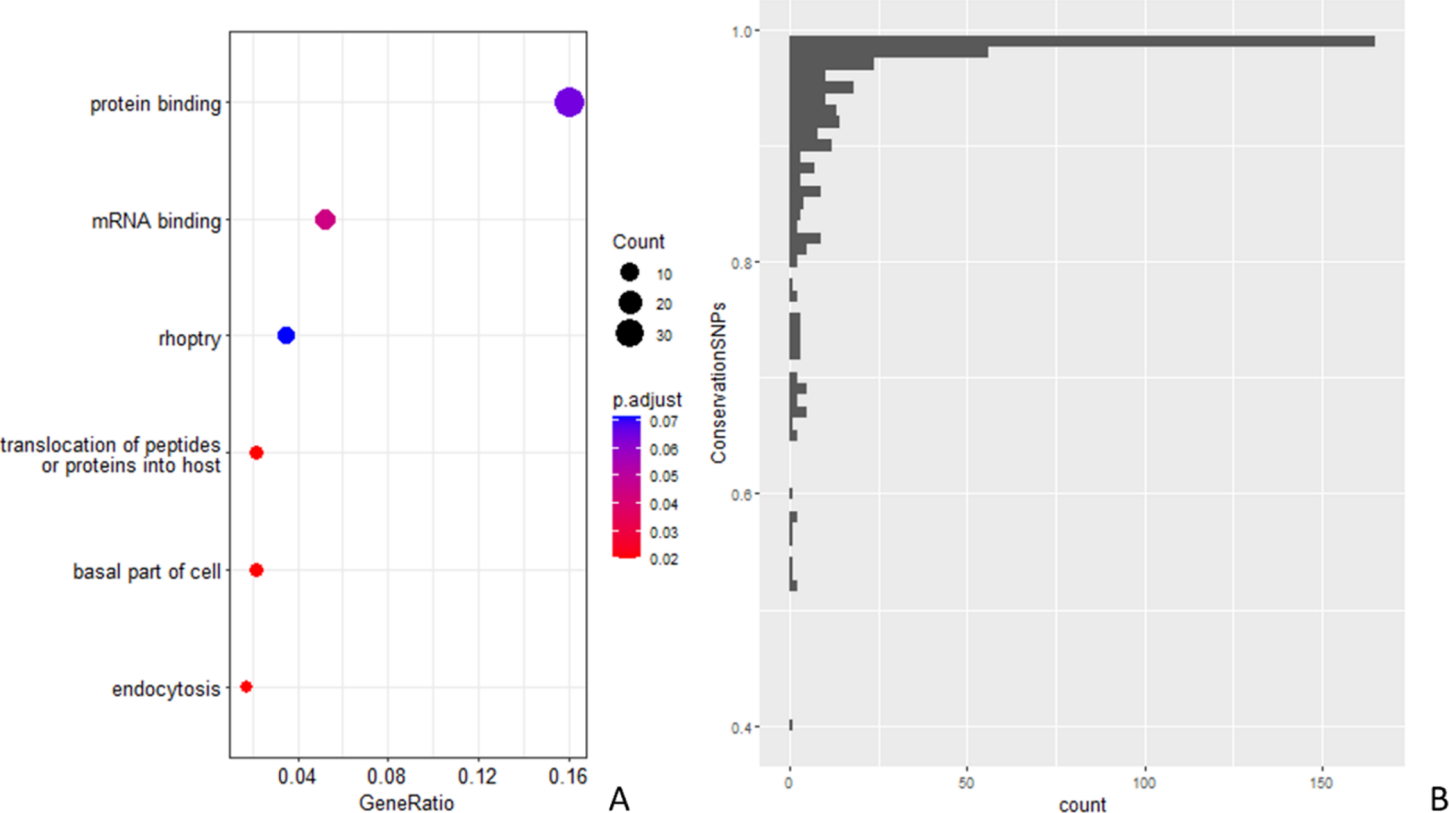
(A) Pathway analysis results across all *Plasmodium* species for those genes, which were not fully conserved compared to PF3D7, based on SNP analysis. (B) Histogram of conservation across *Plasmodium* species based on SNP analysis.

We also investigated conservation based on SNP analysis within the *Plasmodium falciparum* species, using data sets derived from whole genome sequencing of different isolates downloaded from PlasmoDB (Supp. File 7). Out of 26,006 phosphosites, 25,587 did not have any recorded single amino acid variants (SAAVs recorded in PlasmoDB), indicating a very high conservation of phosphosites within the species sampled (98.3%). Analysis of the total serines within the proteome found 4,660 SAAVs with major allele frequency (AF) < 1 (from 261,791 total serines), i.e., 98.2% conservation. This indicates that that pS is no more or less likely to be mutated than other serines. On average, 97.9% threonines in the proteome are conserved (i.e., have no SAAV in this analysis), compared to 98.7% for pT sites. On average, 99.0% of pY (663/670) and 99.0% of all tyrosines do not have a SAAV in this analysis, again indicating no particular selective pressure signal that could be identified. A histogram of the major allele frequencies for phosphosite SAAVs is presented in Figure 5B, confirming that most phosphosites are highly conserved across different isolates, with only a single site (pSer 33 in PF3D7_1366900, a protein of unknown function) having major AF < 0.5. A table of proteins with phosphosites and AF < 0.9 is provided in Supp. File 7, including several zinc finger proteins and two rhoptry proteins.

We performed GO term enrichment analysis for the (419) proteins containing nonfully conserved phosphosites (Figure 5A). Besides “protein binding” (GO:0005515) or “mRNA binding” (GO:0003723) which appear to be significant in most analyses, the analysis also returned “translocation of peptides or proteins into host” (GO:0042000), “rhoptry” (GO:0020008) and “endocytosis” (GO:0006897), indicative perhaps of proteins under some positive selective.

Other comparative analyses of conservation data have been included as Supp. Figure 2. In Supp Figure 2 (A) a pathway analysis was performed comparing the conservation of the phosphosites within three human transmissible species (PmUG01, PocGH01, PVP01) vs 17 not transmissible, to test for any specificity in human invasion-related phosphosites. *P. knowlesi* (PKNH) and *P. vivax*-like (PVL) were excluded from this analysis because of their potential for nonvectorial infection to humans. From phosphoproteins, a subset was selected for pathway analysis as proteins with fully conserved phosphosites for the human transmissible species, i.e., all phosphosites conserved with respect to the reference PF3D7, and not conserved for nontransmissible species (at least one phosphosite within the protein not conserved with respect to the *P. falciparum* reference PF3D7). Some of the significant terms not previously observed in other analyses were “cytosol”, “structural constituent of ribosomes” and “cytosolic small ribosomal subunit”. Supp Figure 2 (B) investigates the enriched pathways for sites within phosphoproteins not conserved in *P. gorilla* clade G1 (PPRFG01), compared to PF3D7, as our analysis (Figure 4A) suggested this species to be the closest relative to *P. falciparium*. This additional analysis returned “infected host cell surface knob” (GO:0020030) as the most significant term, which may indicate differences in the cell invasion between the two species of *Plasmodium*.

### Disorder and Structure Analysis

**Figure 6:**
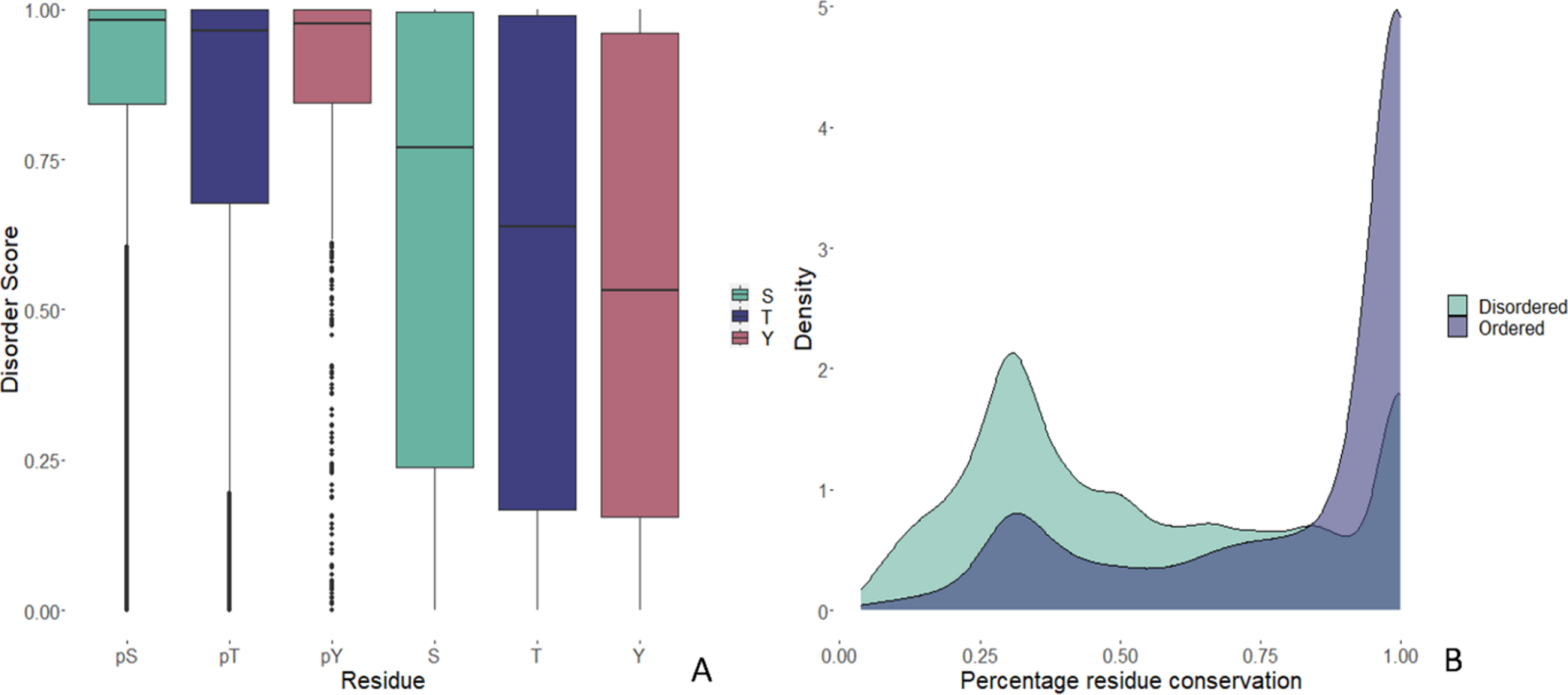
(A) Boxplot of the disorder scores (from metapredict) for phosphosites (pS, pT, and pY) versus disorder scores for all S, T, and Y residues in the *P. falciparum* proteome. (B) Density functions for percentage of conservation by residue for ordered and disordered regions (<0.3 metapredict score).

Next, we explored the structural context of phosphosites in *P. falciparum* to discover any particular trends that point to the functional importance of the phosphosites. First, we performed an analysis to predict all the structured and disordered regions of *P. falciparum* proteins (using metapredict v2) and mapped phosphosites onto these regions. Metapredict gives a score from 0 to 1 to indicate the likelihood of each amino acid within a sequence to be in an ordered (score = 0) or within a disordered region (score = 1). It has been reported before that a high proportion of mammalian phosphosites are located on disordered regions, having a role in transition from disorder to order, altering the local or global structure, and potentially changing the interaction potential of the protein.^58^ From our mapping of phosphosites to predicted disordered regions, we could observe that phosphosites had a very strong tendency toward disordered regions in *P. falciparum*. This tendency for phosphosites to be found in disordered regions can be observed for all three residues S, T, Y. Figure 6A, displays boxplots with strikingly higher disorder scores for phosphosites (pS, pT, pY) than for other S, T or Y residues in the *P. falciparum* proteome–median disorder scores pS = 0.982, pT = 0.965, and pY = 0.975 compared to S = 0.769, T = 0.639 and Y = 0.532 median disorder scores for all residues in the proteome. The trend is particularly striking for pY sites since tyrosines do not have a strong preference to be in disordered regions of proteins. Metapredict documentation suggests that a threshold of 0.3 can differentiate ordered from disordered regions. Using such a threshold across the entire proteome of *P. falciparum* would suggest that 66% of all residues fall in disordered regions. Comparing against metapredict disorder scores for the human proteome (Supplementary Figure 3), revealed that only 39% of residues within the human proteome are located in disordered regions. It is possible that *P. falciparum* proteins are generally more disordered than human proteins or that the tool is less well-calibrated for Apicomplexa than for humans. Nevertheless, using the metapredict-recommended threshold of >0.3 for determining disordered regions, in agreement with previous reports,^53^ revealed that 89% of phosphosites were located within predicted disordered regions (and still 85% if a more conservative score >0.5 was used to determine disorder).

If we restrict the analysis to FLR “gold” category phosphosites (those observed with high confidence in more than one study), remarkably only 5.8% fall in ordered regions (on only 330 proteins), indicating that it is highly unusual for phosphorylation to occur on well-structured regions of *P. falciparum* proteins. For comparison, 12% of “gold standard” human phosphosites^23^ are predicted to fall into ordered regions (metapredict score <0.3). Investigating the biological functions link to these 330 *Plasmodium falciparum* proteins with phosphosites in their ordered regions, there are several GO terms returned from pathway enrichment analysis with clusterProfiler (Supplementary Figure 4), including proteins localizing to the cytosol and cytoplasm and ribosome-related functions.

Next, we wished to explore whether there was any difference in the conservation of phosphosites in disordered versus ordered regions. As shown on Figure 6B, there is a striking difference–phosphosites in ordered regions (2,776) had high conservation overall.

Examining the set of ordered sites, since these are relatively unusual in the *P. falciparum* phosphoproteome, these sites are highly conserved (Figure 6B), compared to disordered phosphosites −48% of all “ordered” sites have conservation >90% across the genus, compared to only 16% of “disordered” sites. We can thus conclude that there is a relatively small number of ancient, highly conserved phosphosites and ordered structures, but the vast majority of phosphosites in *Plasmodium* species map to disordered, fast-evolving regions of proteins.

Exploring the high-quality (gold) set of sites mapped to ordered regions (330 proteins), 209/330 (63%) have a human ortholog (OrthoMCL DB^47^), indicative that these phosphosites are indeed highly conserved across all eukaryotes. For the proteins containing “gold” phosphosites in disordered regions (1724), 718/1724 (42%) have a human orthologue, indicative of proteins that are less well conserved across eukaryotes. All disorder and site conservation data is available as Supp. File 8.

### Protein Structural Context

The release of AlphaFold2 (AF2) has had a very significant impact on the ability to understand the three-dimensional (3D) structure of proteins across both model and nonmodel organisms.^59^ 3D structure predictions for *P. falciparum* proteins are available via the AlphaFold database, UniProtKB and PlasmoDB (via mapping to UniProt identifiers). We have mapped all the phosphosites onto AF2 structures, also incorporating the conservation scores (across the *Plasmodium* genus), enabling visual exploration of the relationship between order/disorder (which can be clearly visualized) on structures, conservation, and positions of phosphosites. In Supp. File 9, we have created an html page with a table of the phosphoproteins identified, with hyperlinks so that every phosphoprotein’s structure and phosphosites can be visualized via the online iCn3D viewer,^51^ and links to their corresponding record in UniProtKB (see section below on data access). A caveat is that AF2 models have no awareness of PTM sites and generally have been trained on few example proteins with PTMs intact. As such, protein structure models demonstrate the position of PTMs as they would appear on an otherwise unmodified structure. Given that phosphosites often change the structure of proteins by introducing more negative charge, it remains an open research question how to remodel AF2 structures to reflect the presence of phosphosites.

**Figure 7:**
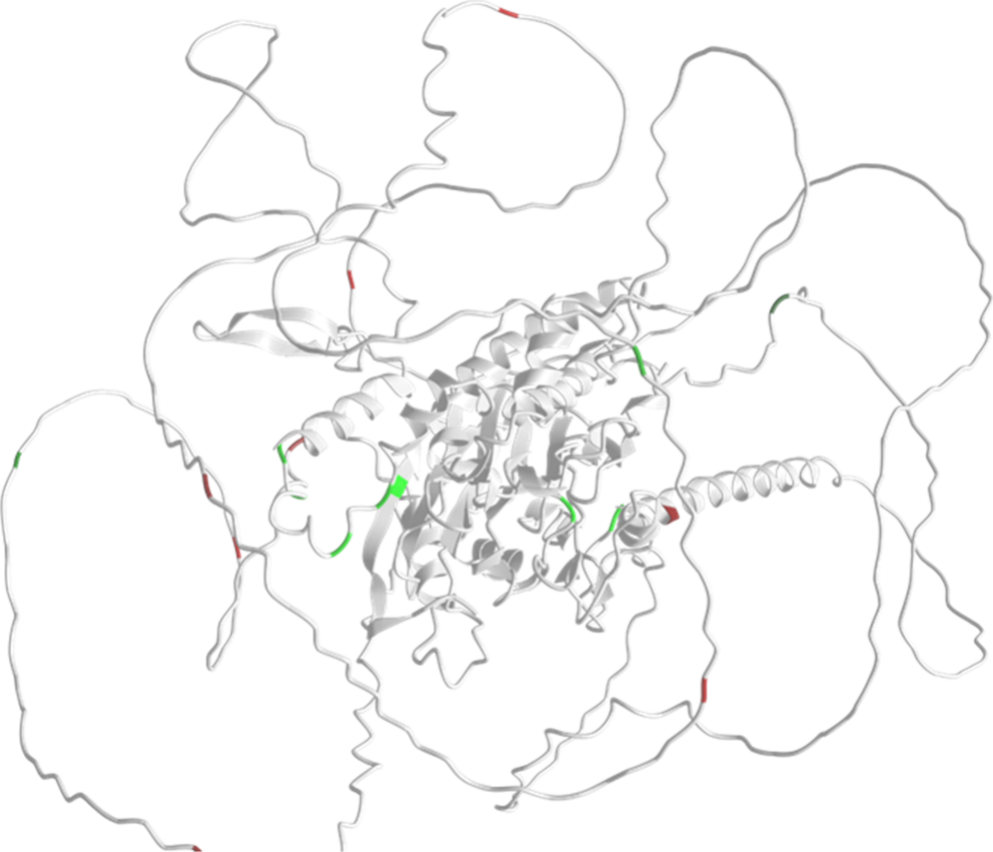
Protein PF3D7_1138500 protein phosphatase PPM2, visualized in iCn3D (AlphaFold structure Q8IHY0), with mapped phosphorylation sites colored red-black-green conservation scale (0–9). Phosphosites in the structured core are fully conserved across the *Plasmodium* genus, whereas disordered regions have mostly low conservation.

An example is presented in Figure 7, for protein phosphatase PPM2 (UniProtKB identifier: Q8IHY0, PlasmoDB identifier: PF3D7_1138500). The image displays all identified phosphosites on the green to red color scale, indicating fully conserved across the genus = green, to unique to *P. falciparum* = red. In PPM2, it can also be observed that phosphosites that are highly conserved (green) are located in the structured core, and that large, disordered regions are around the outside, containing nonconserved phosphosites in red.

### Open Access Data Availability

**Figure 8:**
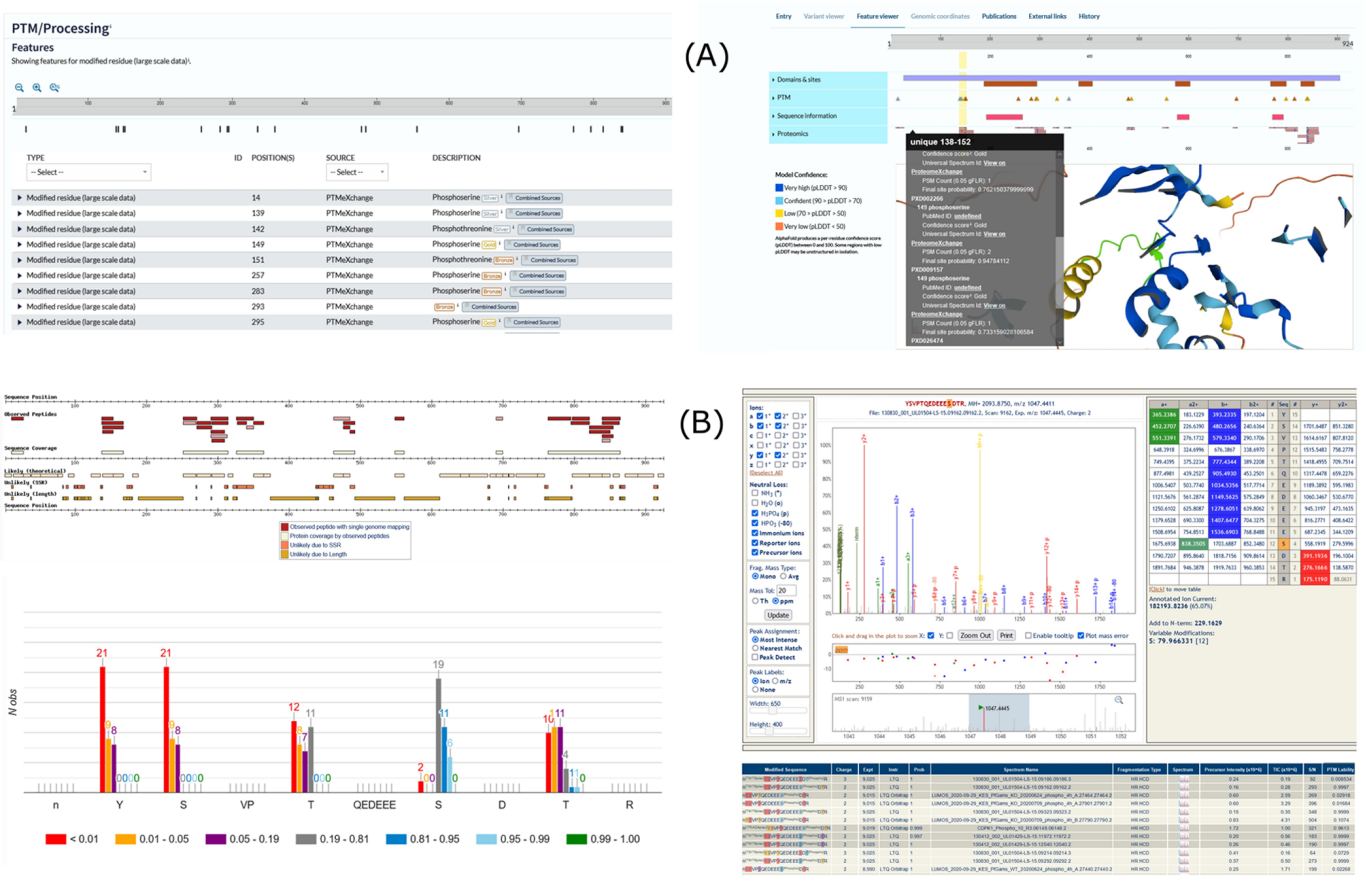
(A) Visualization of phosphosites in UniProtKB for example protein (Q8IHY0). (B) Examples of different visualizations at the protein-, peptidoforms-, and spectral levels for the same protein, with PeptideAtlas identifier PF3D7_1138500.1-p1.

The *Plasmodium falciparum* “phosphosite build” is part of a wider project, called PTMeXchange (https://www.proteomexchange.org/ptmexchange/index.html), aimed at high-quality reanalysis of MS/MS data sets enriched for particular PTMs, and providing simple public access to the resulting data sets. The build is provided via PRIDE/ProteomeXchange with identifier PXD046874, where researchers can download the phosphosites identified per study in simple tab-separated text files. The data has also been loaded into UniProtKB (Figure 8A), enabling phosphosite evidence to be explored alongside other protein features and AF2 models with links to the raw evidence. We have also released the phosphosite build within PeptideAtlas (https://peptideatlas.org/builds/pfalciparum/phospho/), enabling more detailed exploration of the evidence within each protein, peptidoform, and spectrum for a given site (Figure 8B). Our data are deposited in PRIDE, UniProtKB, and PeptideAtlas all provide evidence for each site using Universal Spectrum Identifiers,^60^ which can be rendered via https://proteomecentral.proteomexchange.org/usi/ (and shown in the right panel in Figure 8B). This allows a user to explore the evidence for a given site on a peptide, and test other possible explanations to see if the spectrum could support alternative explanations (peptides or sites within those peptides).

The spectra and corresponding identifications were also converted to spectral libraries in sptxt (SpectraST) format (available from https://peptideatlas.org/builds/pfalciparum/phospho/), enabling their reuse in spectral library searching approaches for new DDA data sets, or data independent acquisition-based quantitative approaches, where a spectral library is typically required.

## Discussion

We have reported a comprehensive meta-analysis of *P. falciparum* phosphoproteomics data sets. Data sets were reanalyzed using a robust pipeline ensuring objective false localization rate calculation. Additional gold, silver, and bronze labeling was also provided as an easy way to display confidence in phosphosites being correctly identified.

We have also provided over-represented motifs found in these phosphoproteins as well as conservation data in relation to another 22 species of the genus *Plasmodium*, and from 115 different *Plasmodium falciparum* isolates, with respect to *P. falciparum* strain 3D7. We provided predictive disorder scores for all identified phosphosites and links to protein structures to visualize these proteins. We concluded that most phosphosites in *Plasmodium* map to disordered regions of proteins, evolving relatively quickly at the genus level, although sites were highly conserved at the species level. When exploring functional relationships across orthologues, it might be typical to conclude that highly conserved regions are most significant for function, and in fact, most protein domains are captured from conserved regions in multiple sequence alignments across assumed orthologues. However, the data presented here do not support such a conclusion for phosphosites. The fact that the vast majority of phosphosites are present on disordered regions of proteins, which are evolving the fastest and seem to have a role in host cell invasion and potentially evading host cell responses, points to a specialization in the functional role of phosphorylation. Further experiments to understand the interplay between phosphorylation, protein disorder, and the ability to infect hosts are clearly required. While AF2 provides a significant advance across species for modeling plausible protein structures, new methods and approaches are required to understand the effects of phosphosites on disordered regions, which are not modeled by current AF2 or similar pipelines.

Results from our meta-analysis have been deposited into UniProtKB, enabling sites to be used for further research with other bioinformatics tools. The data have also been made available in PeptideAtlas and PRIDE, enabling detailed exploration of scores and visualization of source mass spectra, as a full evidence trail. We expect our results to be a useful resource for researchers facilitating the identification of areas for future research in developing malaria treatments and vaccines.

## Supporting Information Available

Supp Table 1. Study synopsis extracted from abstracts and search parameters used for each study included in this analysis. Supp Figure 1. Full list of phosphorylation motifs, identified from motif analysis, using rmotifx, from a foreground of all phosphosites identified at 5% FLR, against a background of all STY residues in the database. Supp Table 2. Counts of P. falciparum kinases in different kinase groups *=(including 4 EIF2AK and assorted others—weak matches to mammalian homologues) Supp Figure 2 A: Pathway analysis results for those proteins in 3 human transmissible species vs 17 not transmissible to humans. The list of genes was generated from the proteins with fully conserved phosphosites for the 3 human transmissible species with respect to the reference but not for any one or more of the species in the no transmissible group. B: Enrichment analysis of those genes not conserved for PPRFG01 with respect to the reference PF3D7. Supp Figure 3. Histogram of disorder scores for the P. falciparum proteome (Pf) and human proteome (Hs). Supp Figure 4. Dotplot of the GO analysis of the 330 phosphoproteins, containing “gold” standard sites in the ordered regions of P. falciparum (PDF)
Supp File 1. GSB_withMotifs.txt: Phosphosites mapped to all source proteins, at 5% FLR classified as gold, silver, and bronze, PSM counts, data sets detected, and statistically significant motifs for those sites (tab-separated text) (TXT)
Supp File 2. EnrichmentResult_Allmotifs_05.xlsx: Enrichment analysis results for all statistically significant motifs (XLSX)
Supp File 3. Conservation.txt: Conservation scores across species with respect to the reference 3D7 (tab-separated text) (TXT)
Supp File 4. ConservationSpeciesWithClusters.txt: Proportion of sites conserved within each protein and species with respect to 3D7 and its clusters (tab-separated text) (TXT)
Supp File 5. mapped_plasmodium_sites.txt: Phosphosites putatively identified in other Plasmodium species based on orthologue mapping (tab-separated text) (TXT)
Supp File 6. plasmodium_alignments.zip: Multiple sequence aligments of orthologous phosphoproteins within the Plasmodium genus (ZIP)
Supp File 7. SNP_data.xlsx: Conservation analysis based on single amino acid variants (XLSX)
Supp File 8. Disorder.txt: Disorder scores from metapredict for gold, silver, and bronze phosphosites (tab-separated text) (TXT)
Supp File 9. Proteins_3D.pdf: Hyperlinks for visualizing identified phosphoproteins in iCn3D viewer (PDF)
