# Supplementary material for: Phosphorylation in the Plasmodium falciparum Proteome: A Meta-Analysis of Publicly Available Data Sets
Source: J Proteome Res. 2024 Oct 30;23(12):5326–41. doi: 10.1021/acs.jproteome.4c00418 (PMC11629380; doi:10.1021/acs.jproteome.4c00418)
Supplement: Supplementary file 1 — pr4c00418_si_001.pdf [file pr4c00418_si_001.pdf]

Supplementary Information for:

Phosphorylation in the *Plasmodium falciparum* proteome: A meta-analysis of publicly available data sets

*Oscar J M Camacho[1], Kerry A Ramsbottom[1], Ananth Prakash[2], Yasset Perez Riverol[2], Emily Bowler-Barnett[2], Maria Martin[2], Jun Fan[2], Eric W Deutsch[3], Juan Antonio Vizcaíno[2] and Andrew R Jones[1]**

[1]Institute of Systems, Molecular and Integrative Biology, University of Liverpool, Liverpool, L69 3BX, United Kingdom

[2]European Molecular Biology Laboratory, EMBL-European Bioinformatics Institute (EMBL-EBI), Hinxton, Cambridge, CB10 1SD, United Kingdom.

[3]Institute for Systems Biology, Seattle, Washington 98109, United States

*Andrew.Jones@liverpool.ac.uk

Table of Contents

Supp File 1. GSB_withMotifs.txt : Phosphosites mapped to all source proteins, at 5% FLR classified as Gold, Silver and Bronze, PSM counts, datasets detected in, and statistically significant motifs for those sites (tab-separated text).

Supp File 2. EnrichmentResult_Allmotifs_05.xlsx : Enrichment analysis results for all statistically significant motifs.

Supp File 3. Conservation.txt : Conservation scores across species with respect to the reference 3D7 (tab-separated text).

Supp File 4. ConservationSpeciesWithClusters.txt : Proportion of sites conserved within each protein and species with respect to 3D7 and its clusters (tab-separated text).

Supp File 5. mapped_plasmodium_sites.txt : Phosphosites putatively identified in other *Plasmodium* species based on orthologue mapping (tab-separated text)..

Supp File 6. plasmodium_alignments.zip : Multiple sequence aligments of orthologous phosphoproteins within the *Plasmodium* genus.

Supp File 7. SNP_data.xlsx : Conservation analysis based on single amino acid variants.

Supp File 8. Disorder.txt : Disorder scores from metapredict for Gold, Silver and Bronze phosphosites (tab-separated text).

Supp File 9. Proteins_3D.pdf : Hyperlinks for visualising identified phosphoproteins in iCn3D viewer.

Supplementary Information.docx : Suplementary tables and figures.

Supp Table 1 Study synopsis extracted from abstracts and search parameters used for each study included in this analysis.

Study ID	Summary	Enrichment Method	Fragmentation Type	Labelling Type	Peptide Tolerance	Fragment Tolerance	Variable Modifications	Fixed Modifications
PXD000070	"We analyzed the Plasmodium falciparum schizont phosphoproteome using for the first time a data-dependent neutral loss-triggered-ETD..."	Pre-equilibrated Phos-Select beads (Sigma) for 1 h	CID and ETD	Unlabelled	20 ppm	1.0005 Da	pASTY, PYRO_QC, HYDR_MW, ACET_Kn, FORM_n, DHB_E	C
PXD001684	"Phosphoproteome analysis of extracellular merozoites revealing 1765 unique phosphorylation sites..."	Titansphere 10 µm TiO2 beads	CID	Unlabelled	20 ppm	1.0005 Da	pASTY, PYRO_QC, HYDR_MW, ACET_Kn, FORM_n, DHB_E	C
PXD002266	"Employing chemical and genetic tools in combination with quantitative global phosphoproteomics, we identify the phosphorylation sites..."	TiO2 beads	HCD	TMT6	10 ppm	0.2 Da	pASTY, PYRRO_QC, HYDR_MW	C
PXD005207	"PfCDPK1 is critical for asexual development of Plasmodium falciparum, involving comparative phosphoproteomics..."	TiO2 based enrichment	HCD	4-plex iTRAQ	20 ppm	0.2 Da	pASTY, PYRO_QC, HYDR_MW, DEAM_NQ, DHB_E	C
PXD009157	"Plasmodium falciparum phosphodiesterase β (PDEβ) hydrolyzes both cAMP and cGMP and is essential for blood stage viability..."	Titanium dioxide beads (5:1 [w/w] beads)	HCD	TMT6	10 ppm	0.2 Da	pASTY, HYDR_MW, DEAM_NQ	C
PXD009465	"To better understand PfPK7-regulated phosphorylation events, we performed isobaric tag-based quantitative comparative phosphoproteomics..."	IMAC	CID	TMT6	10 ppm	0.2 Da	pASTY, HYDR_MW, DEAM_NQ	C
PXD012143	"Investigated the role of cAMP in asexual blood stage development of Plasmodium falciparum..."	Sequential metal oxide affinity chromatography (SMOAC) strategy	MS2 HCD, MSA SPS MS3	TMT10	10 ppm	0.2 Da	pASTY, HYDR_MW, DEAM_NQ	C
PXD015093	"Global phosphoproteomic analysis of merozoites to identify signaling pathways activated during invasion..."	TiO2/ZrO2 NuTip	HCD	TMT6 & Unlabelled	20 ppm	0.2 Da	pASTY, PYRO_QC, HYDR_MW, ACET_Kn, FORM_n, DHB_E	C
PXD015833	"Reports species-specific phosphorylation of erythrocyte proteins by P. falciparum but not by Plasmodium knowlesi..."	TiO2 beads	MS2 HCD, CID MS3	TMT10	10 ppm	0.2 Da	pASTY, DEAM_NQ	C
PXD020381	"Identified a multipass membrane protein, ICM1, associated with PKG in both asexual blood stages and transmission stages..."	Sequential metal oxide affinity chromatography strategy	MS2 HCD, MSA SPS MS3	TMT10	10 ppm	0.2 Da	pASTY, HYDR_MW, DEAM_NQ	C
PXD026474	"Understand CDPKs' role in human parasite transmission from host to mosquito vector, investigating P. falciparum CDPK4's role..."	Metal affinity chromatography (IMAC) beads	HCD	Unlabelled	20 ppm	0.2 Da	pASTY, PYRO_QC, HYDR_MW, ACET_Kn, FORM_n, DHB_E	C

.......SD......	......DT.S.....	.......SND.....	....R..S.......
.......SE......	......DTD......	.......SNE.....	....R..S....E..
.......S...D...	....D..S.......	......NS.D.....	....R..S..N....
.......S...E...	....D.ES.......	......NS.E.....	....R..SN......
.......S..D....	....E..S.......	......NT.D.....	....R..T.......
.......S..E....	....E..T.E.....	.....N.S.D.....	....R..T.E.....
.......S.D.....	....E..TE......	.....N.S.E.....	....R.NS.......
.......S.E.....	...K...S.D.....	.....N.SD......	......RSF.D....
......ES.......	...K...S.E.....	......GSN......	.....R.S.......
......DS.......	.K.....SD......	......NS.......	.K..R..S.......
.......SE.E....	.K....DS.......	.......SN......	....KR.S.......
.....D.TE......	.......S.D...K.	.......SN.....K	......GS.......
.......SD.D....	....K..S.......	......NT.......	.......S.S.....
.......SD.E....	....K..S......K	....K.NS.......	....S..S.......
.......T...D...	....K..S.D.....	....K..SN......	....S..Y.......
.......T..DE...	....K..T.......	....H.NS.......	.......Y...S...
.......T..E....	....K..T.D.....	K.....NS.......	.......Y..S....
.......T.D.....	....K..T.E.....	...K...S.N.....	.......Y.S.....
.......T.DE....	....KS.S.......	....SN.T.......	.......YS......
.......T.E.....	K.....DS.......	.....N.SP......	.......Y.SD....
.......T.EE....	.......S.E...K.	.......SP......	.......Y..D....
.......T.S.....	.......S.E..K..	.......SP..K...	.......YSD.....
.......TD......	.......SD.....K	.......TP......	......SY.......
.......TD..D...	.......SD....K.	.......TP...E..	......SYE......
.......TD.E....	.......SD.E..K.	.......TP.K....	.....S.Y.......
.......TDD.....	....K..S.EN....	....K..TP......	...S...Y.......
......DT.......	....KK.S.......	....N..SP......	

Supp Figure 1 Full list of phosphorylation motifs, identified from motif analysis, using rmotifx, from a foreground of all phosphosites identified at 5% FLR, against a background of all STY residues in the database.

*Supp Table 2 Counts of P. falciparum kinases in different kinase groups *=(including 4 EIF2AK and assorted others - weak matches to mammalian homologs)*

**Kinase Group**	***Count of P. falciparum kinases***	**Potential Motif, based on knowledge from mammalian systems** [54]
AGC	6	pTX[R/K]
CAMK	20	R or K in positions -2 or -3
CK1	1	Enriched in nearby phosphosites, potential tyrosine activity as well as S/T
CMGC	18	Proline enriched at +1 site
FIKK	21	Apicomplexan specific family, potentially targeting an arginine-enriched motif in the minus positions, according to [30]
NEK	4	Favours pT, followed by M, Y, F, W or V at +1 position
Orphan*	20	EIF2AK have motif D/E enriched at -2 position, others unknown
STE	1	Only weak match between 3D7 kinase and human STE family, so motif cannot be inferred.

TKL	7	Unclear motif. These are annotated as tyrosine-kinase like, but are not orthologous with mammalian tyrosine kinases.
Total	98	

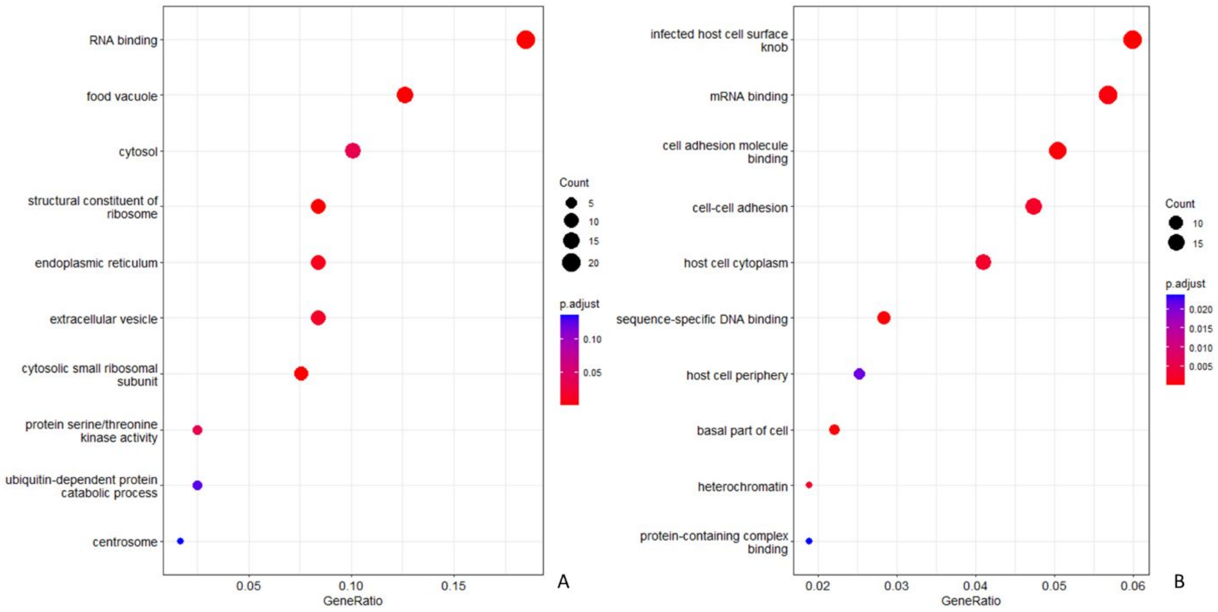

Supp Figure 2 A: Pathway analysis results for those proteins in 3 human transmissible species vs. 17 not transmissible to humans. The list of genes was generated from the proteins with fully conserved phosphosites for the 3 human transmissible species with respect to the reference but not for any one or more of the species in the no transmissible group. B: Enrichment analysis of those genes no conserved for PPRFG01 with respect to the reference PF3D7.

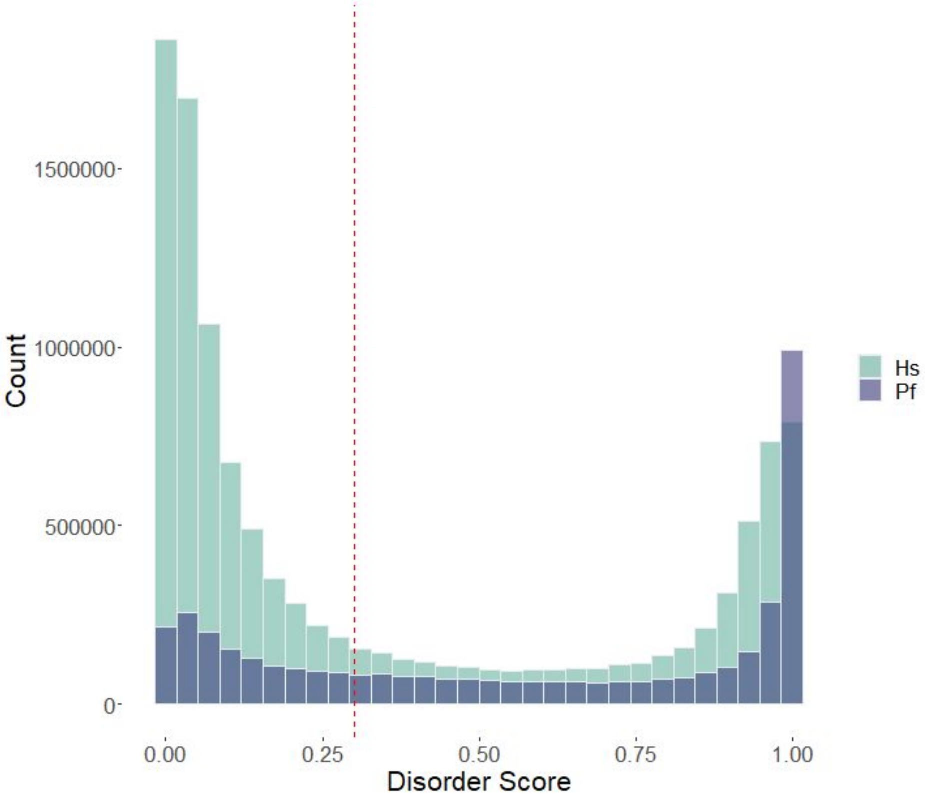

Supp Figure 3. Histogram of disorder scores for the *P. falciparum* proteome (Pf) and Human proteome (Hs).

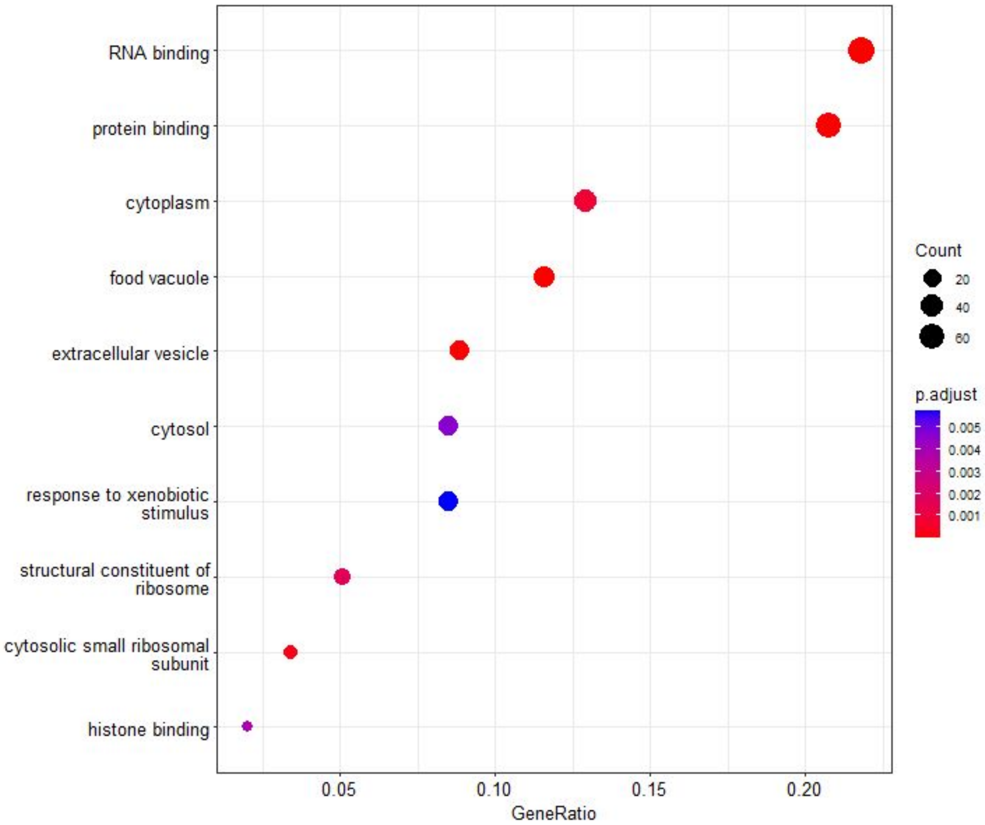

Supp Figure 4. Dotplot of the GO analysis of the 330 phosphoproteins, containing "Gold" standard sites in the ordered regions of *P. falciparum.*
