# Supplementary material for: Phosphorylation in the Plasmodium falciparum Proteome: A Meta-Analysis of Publicly Available Data Sets
Source: J Proteome Res. 2024 Oct 30;23(12):5326–41. doi: 10.1021/acs.jproteome.4c00418 (PMC11629380; doi:10.1021/acs.jproteome.4c00418)
Supplement: Supplementary file 10 — pr4c00418_si_010.pdf [file pr4c00418_si_010.pdf]

Protein (PlasmoDB)	Protein (UniProt)	Product Description	Site count	iCn3d view
PF3D7_1366900	Q8ID39	conserved protein, unknown function	21	iCn3D view
PF3D7_0914000	Q8I312	pseudouridylate synthase, putative	6	iCn3D view
PF3D7_1221000	Q8I5K3	histone-lysine N-methyltransferase, H3 lysine-4 specific	32	iCn3D view
PF3D7_0929400	C0H571	high molecular weight rhoptry protein 2	7	iCn3D view
PF3D7_1033100	Q8IJ77	S-adenosylmethionine decarboxylase/ornithine decarboxylase	11	iCn3D view
PF3D7_1216400	Q8I5P3	conserved Plasmodium membrane protein, unknown function	7	iCn3D view
PF3D7_1004200	Q8IJZ5	WD repeat-containing protein, putative	27	iCn3D view
PF3D7_0507800	Q8I425	conserved protein, unknown function	20	iCn3D view
PF3D7_1119900	Q8IIG8	protein transport protein SEC16, putative	27	iCn3D view
PF3D7_0930300	Q8I0U8	merozoite surface protein 1	15	iCn3D view
PF3D7_0519600	Q8I3R4	zinc finger protein, putative	9	iCn3D view
PF3D7_0709700	Q8IBZ2	prodrug activation and resistance esterase	12	iCn3D view
PF3D7_0311300	O77353	phosphatidylinositol 3- and 4-kinase, putative	22	iCn3D view
PF3D7_0905400	Q8I395	high molecular weight rhoptry protein 3	18	iCn3D view
PF3D7_0912800	Q8I324	tRNA (adenine(58)-N(1))-methyltransferase non-catalytic subunit TRM6, putative	7	iCn3D view
PF3D7_1107300	Q8IIS9	polyadenylate-binding protein-interacting protein 1, putative	99	iCn3D view
PF3D7_1411200	Q8ILY3	rhomboid protease ROM8	19	iCn3D view
PF3D7_1431500	Q8ILF0	mitogen-activated protein kinase 1	5	iCn3D view
PF3D7_0308300	O77324	PhIL1-interacting candidate PIC4	25	iCn3D view
PF3D7_0501800	Q8I482	chromatin assembly factor 1 subunit A	24	iCn3D view
PF3D7_0103100	Q8I2A9	vacuolar protein sorting-associated protein 51, putative	13	iCn3D view
PF3D7_0215600	O96235	Golgi to ER traffic protein 2	2	iCn3D view
PF3D7_0704600	C0H4K6	HECT-type E3 ubiquitin ligase UT	33	iCn3D view

PF3D7_0710200	Q8IBY8	conserved Plasmodium protein, unknown function	22	iCn3D view
PF3D7_0721000	Q8IBP1	conserved Plasmodium membrane protein, unknown function	10	iCn3D view
PF3D7_0615900	A0A5K1K895	protein phosphatase, putative	8	iCn3D view
PF3D7_1300600	Q8IEU6	rifin	1	iCn3D view
PF3D7_1035200	Q03400	S-antigen	7	iCn3D view
PF3D7_1036900	A0A143ZYL5	conserved Plasmodium protein, unknown function	67	iCn3D view
PF3D7_1468900	Q8IKE8	zinc finger protein, putative	13	iCn3D view
PF3D7_0603800	C6KSN6	NdP2 protein, putative	7	iCn3D view
PF3D7_0615600	C6KSZ7	zinc finger protein, putative	12	iCn3D view
PF3D7_1138500	Q8IHY0	protein phosphatase PPM2	19	iCn3D view
PF3D7_0202000	Q9TY99	knob-associated histidine-rich protein	45	iCn3D view
PF3D7_0711500	C0H4M6	regulator of chromosome condensation, putative	32	iCn3D view
PF3D7_1016300	Q8I6U8	GBP130 protein	14	iCn3D view
PF3D7_1031300	Q8IJ92	SAE2 domain-containing protein, putative	33	iCn3D view
PF3D7_1215400	Q8I5Q2	conserved Plasmodium protein, unknown function	9	iCn3D view
PF3D7_1440800	Q8IL62	major facilitator superfamily domain-containing protein, putative	9	iCn3D view
PF3D7_0214100	O96221	protein transport protein SEC31	29	iCn3D view
PF3D7_1203300	Q8I612	TBCC domain-containing protein, putative	4	iCn3D view
PF3D7_0936000	Q8I2G0	ring-exported protein 2	4	iCn3D view
PF3D7_1353400	Q8IDG6	Ran-binding protein, putative	19	iCn3D view
PF3D7_0415200	Q8I1T6	conserved Plasmodium protein, unknown function	17	iCn3D view
PF3D7_0802300	Q8IAM3	periodic tryptophan protein 2, putative	5	iCn3D view
PF3D7_0913600	Q8I316	conserved Plasmodium protein, unknown function	5	iCn3D view
PF3D7_1209400	Q8I5V5	cytosolic iron-sulfur protein assembly protein 1, putative	18	iCn3D view
PF3D7_1429400	Q8ILH0	rRNA (adenosine-2'-O-)-methyltransferase, putative	7	iCn3D view

PF3D7_0106000	B9ZSI3	conserved protein, unknown function	4	iCn3D view
PF3D7_0308100	O77322	zinc finger protein, putative	35	iCn3D view
PF3D7_0323700	O97303	U4/U6.U5 tri-snRNP-associated protein 1, putative	18	iCn3D view
PF3D7_0501400	Q8I486	interspersed repeat antigen	16	iCn3D view
PF3D7_0704300	A0A143ZVW7	basal complex protein BLEB	68	iCn3D view
PF3D7_0720900	A0A143ZWF7	tetratricopeptide repeat protein, putative	2	iCn3D view
PF3D7_1209500	Q8I5V4	cGMP-specific 3',5'-cyclic phosphodiesterase alpha	10	iCn3D view
PF3D7_1211900	A0A143ZZK9	non-SERCA-type Ca2+ -transporting P-ATPase	13	iCn3D view
PF3D7_1308400	C0H5A6	conserved Plasmodium protein, unknown function	60	iCn3D view
PF3D7_1207200	Q8I5X3	zinc finger protein, putative	3	iCn3D view
PF3D7_0317400	O77383	DNA replication complex GINS protein, putative	4	iCn3D view
PF3D7_0407800	Q9U0K8	protein CINCH	87	iCn3D view
PF3D7_0401800	Q8I207	Plasmodium exported protein (PHISTb), unknown function	31	iCn3D view
PF3D7_0613900	C6KSY1	myosin E, putative	47	iCn3D view
PF3D7_1343800	C0H5G6	VPS13 domain-containing protein, putative	108	iCn3D view
PF3D7_0715800	Q8IBU2	drug/metabolite transporter DMT1, putative	6	iCn3D view
PF3D7_0716100	Q8IBT9	protein SDA1, putative	6	iCn3D view
PF3D7_0730500	Q8IBF4	conserved Plasmodium protein, unknown function	64	iCn3D view
PF3D7_1323800	A0A5K1K8G8	vacuolar protein sorting-associated protein 52, putative	2	iCn3D view
PF3D7_1415600	Q8ILU0	conserved Plasmodium protein, unknown function	3	iCn3D view
PF3D7_1419400	Q8ILQ6	conserved Plasmodium membrane protein, unknown function	33	iCn3D view
PF3D7_0110100	Q8I243	selenocysteine-specific elongation factor, putative	5	iCn3D view
PF3D7_0413100	Q8I1V6	erythrocyte membrane protein 1, PfEMP1	1	iCn3D view
PF3D7_0704200	Q8IC28	RNA cytosine C(5)-methyltransferase, putative	7	iCn3D view
PF3D7_0305600	O97240	DNA-(apurinic or apyrimidinic site) endonuclease	13	iCn3D view

PF3D7_0412400	Q9U0G6	erythrocyte membrane protein 1, PfEMP1	2	iCn3D view
PF3D7_0803600	A0A146M1U1	GRAM domain-containing protein, putative	13	iCn3D view
PF3D7_1014900	Q8IJP6	protein KIC8	111	iCn3D view
PF3D7_1032900	Q8IJ79	RNA polymerase II-associated protein 1, putative	2	iCn3D view
PF3D7_1108000	Q8IIS2	IWS1-like protein, putative	4	iCn3D view
PF3D7_0113000	Q9U0N1	glutamic acid-rich protein GARP	10	iCn3D view
PF3D7_1237200	Q8I551	conserved Plasmodium protein, unknown function	10	iCn3D view
PF3D7_0104300	Q8I296	ubiquitin carboxyl-terminal hydrolase 1, putative	81	iCn3D view
PF3D7_0505700	Q8I445	conserved Plasmodium membrane protein, unknown function	8	iCn3D view
PF3D7_0706500	C0H4L6	conserved Plasmodium protein, unknown function	31	iCn3D view
PF3D7_0725000	Q8IBK1	exonuclease I, putative	23	iCn3D view
PF3D7_0801000	Q8IAK9	Plasmodium exported protein (PHISTc)	10	iCn3D view
PF3D7_0804500	A0A143ZZV5	conserved Plasmodium membrane protein, unknown function	71	iCn3D view
PF3D7_0819700	C0H4W1	conserved Plasmodium protein, unknown function	2	iCn3D view
PF3D7_1104900	Q8IIV3	calcium/calmodulin-dependent protein kinase, putative	11	iCn3D view
PF3D7_1237900	Q8I544	conserved Plasmodium protein, unknown function	59	iCn3D view
PF3D7_1319400	A0A5K1K826	conserved protein, unknown function	8	iCn3D view
PF3D7_1400200	Q8IM85	rifin	1	iCn3D view
PF3D7_0403200	Q8I1Z8	pre-mRNA splicing factor, putative	17	iCn3D view
PF3D7_0515300	Q8I3V5	phosphatidylinositol 3-kinase	14	iCn3D view
PF3D7_0531100	Q8I3F7	conserved Plasmodium protein, unknown function	17	iCn3D view
PF3D7_0726300	Q8IBJ3	DNA mismatch repair protein PMS1, putative	2	iCn3D view
PF3D7_1035000	Q8IJ59	U2 snRNA/tRNA pseudouridine synthase, putative	2	iCn3D view
PF3D7_1401200	Q8IM76	Plasmodium exported protein, unknown function	9	iCn3D view
PF3D7_0302500	O77310	cytoadherence linked asexual protein 3.1	15	iCn3D view

PF3D7_0420000	Q8I1N9	zinc finger protein, putative	29	iCn3D view
PF3D7_0610200	C6KSU9	RNA-binding protein 25, putative	9	iCn3D view
PF3D7_0707300	C0H4M0	rhoptry-associated membrane antigen	8	iCn3D view
PF3D7_0714600	Q8IBV1	conserved protein, unknown function	14	iCn3D view
PF3D7_0725100	Q8IBK0	conserved Plasmodium membrane protein, unknown function	3	iCn3D view
PF3D7_0822900	Q8IB63	PhIL1-interacting candidate PIC2	85	iCn3D view
PF3D7_1114900	Q8IIL3	filamin domain-containing protein, putative	15	iCn3D view
PF3D7_1122900	Q8IID4	dynein heavy chain, putative	10	iCn3D view
PF3D7_1218300	Q8I5M5	AP-2 complex subunit mu	9	iCn3D view
PF3D7_1240600	Q8I519	erythrocyte membrane protein 1, PfEMP1	1	iCn3D view
PF3D7_1306800	Q8IEP7	RED-like protein, putative	6	iCn3D view
PF3D7_0217600	O96256	conserved Plasmodium protein, unknown function	15	iCn3D view
PF3D7_0307700	O97255	apicomplexan kinetochore protein 10, putative	40	iCn3D view
PF3D7_0812100	Q8IAV6	proteasome activator complex subunit 4, putative	25	iCn3D view
PF3D7_0903300	C0H517	conserved protein, unknown function	9	iCn3D view
PF3D7_1201500	Q8I630	GPN-loop GTPase, putative	6	iCn3D view
PF3D7_1310400	Q8IEL1	conserved Plasmodium protein, unknown function	15	iCn3D view
PF3D7_1316100	Q8IEF3	inositol polyphosphate kinase, putative	22	iCn3D view
PF3D7_1335300	C0H5F4	reticulocyte binding protein 2 homologue b	2	iCn3D view
PF3D7_1335400	Q8IDX6	reticulocyte binding protein 2 homologue a	4	iCn3D view
PF3D7_1403100	C6S3H2	condensin complex subunit 1, putative	12	iCn3D view
PF3D7_0503300	Q8I468	serine/arginine-rich splicing factor 12	29	iCn3D view
PF3D7_0532300	Q8I3F1	Plasmodium exported protein (PHISTb), unknown function	6	iCn3D view
PF3D7_0606600	C6KSR4	WD repeat-containing protein, putative	36	iCn3D view
PF3D7_0830500	Q8IBD1	sporozoite and liver stage tryptophan-rich protein, putative	10	iCn3D view

PF3D7_0919800	Q8I2V5	TLD domain-containing protein	20	iCn3D view
PF3D7_1228400	Q8I5D4	conserved Plasmodium protein, unknown function	14	iCn3D view
PF3D7_1417200	Q8ILS4	CCR4-NOT transcription complex subunit NOT1, putative	36	iCn3D view
PF3D7_1443100	Q8IL40	apicomplexan kinetochore protein 9, putative	12	iCn3D view
PF3D7_0113200	B9ZSJ1	Plasmodium exported protein, unknown function	17	iCn3D view
PF3D7_0304100	O97229	inner membrane complex protein 1e, putative	13	iCn3D view
PF3D7_0621100	C6KT49	tetratricopeptide repeat protein, putative	9	iCn3D view
PF3D7_0730900	Q8IBF2	EMP1-trafficking protein	32	iCn3D view
PF3D7_0910200	Q8I350	conserved Plasmodium protein, unknown function	32	iCn3D view
PF3D7_1002100	Q8IK15	EMP1-trafficking protein	16	iCn3D view
PF3D7_1021800	A0A143ZXM2	schizont egress antigen-1	60	iCn3D view
PF3D7_1026600	Q8IJD6	conserved Plasmodium protein, unknown function	36	iCn3D view
PF3D7_1307900	Q8IEN6	tripartite motif protein, putative	20	iCn3D view
PF3D7_1359600	A0A5K1K8U0	conserved Plasmodium protein, unknown function	46	iCn3D view
PF3D7_1405100	Q8IM43	GTPase-activating protein, putative	21	iCn3D view
PF3D7_1433400	Q8ILC9	PHD finger protein PHD2, putative	68	iCn3D view
PF3D7_1446500	Q8IL08	nucleoporin NUP313, putative	44	iCn3D view
PF3D7_1448500	Q8IKY8	conserved Plasmodium protein, unknown function	22	iCn3D view
PF3D7_1458500	Q8IKP6	spindle assembly abnormal protein 4, putative	25	iCn3D view
PF3D7_1474200	Q8IK96	conserved Plasmodium membrane protein, unknown function	4	iCn3D view
PF3D7_0309000	O77334	dual specificity protein phosphatase	5	iCn3D view
PF3D7_0522400	C0H4F8	conserved Plasmodium protein, unknown function	123	iCn3D view
PF3D7_0717200	C0H4N2	conserved Plasmodium protein, unknown function	7	iCn3D view
PF3D7_0820000	C0H4W3	Snf2-related CBP activator, putative	28	iCn3D view
PF3D7_1209300	Q7KQK4	telomere repeat-binding zinc finger protein	37	iCn3D view

PF3D7_1317800	C0H5C2	40S ribosomal protein S19	5	iCn3D view
PF3D7_1318300	Q8IED3	conserved Plasmodium protein, unknown function	22	iCn3D view
PF3D7_1368200	Q8I6Z4	ABC transporter E family member 1, putative	3	iCn3D view
PF3D7_0201500	O96120	Plasmodium exported protein (hyp9), unknown function	4	iCn3D view
PF3D7_0212500	O96205	nucleoporin NUP434, putative	39	iCn3D view
PF3D7_0313000	O77363	conserved Plasmodium protein, unknown function	7	iCn3D view
PF3D7_0500800	Q8I492	mature parasite-infected erythrocyte surface antigen	51	iCn3D view
PF3D7_0510100	Q8I403	KH domain-containing protein, putative	44	iCn3D view
PF3D7_0616900	C6KT10	conserved Plasmodium protein, unknown function	13	iCn3D view
PF3D7_0703200	Q8IC38	conserved Plasmodium protein, unknown function	13	iCn3D view
PF3D7_0704800	Q8IC22	protein phosphatase PPM12, putative	48	iCn3D view
PF3D7_0831300	C0H4Z6	Plasmodium exported protein, unknown function	5	iCn3D view
PF3D7_0904900	Q8I3A0	copper-transporting ATPase	37	iCn3D view
PF3D7_0907700	Q8I374	proteasome activator 28	5	iCn3D view
PF3D7_1008000	Q8IJW3	histone deacetylase 2	42	iCn3D view
PF3D7_1126700	Q8II97	conserved Plasmodium protein, unknown function	13	iCn3D view
PF3D7_1232400	Q8I598	CWC16 domain-containing protein, putative	3	iCn3D view
PF3D7_1337900	A0A5K1K8U7	conserved Plasmodium protein, unknown function	3	iCn3D view
PF3D7_1449100	Q8IKY3	CLASP domain-containing protein, putative	19	iCn3D view
PF3D7_1467600	A0A144A2Q6	conserved Plasmodium protein, unknown function	43	iCn3D view
PF3D7_1468100	Q8IKF6	MORC family protein	83	iCn3D view
PF3D7_0110800	Q8I237	transcription initiation factor TFIIB, putative	2	iCn3D view
PF3D7_0201900	O96124	erythrocyte membrane protein 3	17	iCn3D view
PF3D7_0303100	O77314	CLP1 P-loop domain-containing protein, putative	19	iCn3D view
PF3D7_0305100	O97236	conserved Plasmodium protein, unknown function	11	iCn3D view

PF3D7_0411300	Q9U0H5	golgin subfamily A member 6-like protein, putative	10	iCn3D view
PF3D7_0424700	Q8IFL9	serine/threonine protein kinase, FIKK family	9	iCn3D view
PF3D7_0513600	Q8I0W8	deoxyribodipyrimidine photo-lyase, putative	11	iCn3D view
PF3D7_0530300	C0H4G8	PhIL1-interacting candidate PIC6	26	iCn3D view
PF3D7_0606000	C6KSQ8	protein KIC1	64	iCn3D view
PF3D7_0613300	C6KSX6	rhoptry protein ROP14	5	iCn3D view
PF3D7_0703900	A0A143ZY62	conserved Plasmodium membrane protein, unknown function	15	iCn3D view
PF3D7_0705100	Q8IC19	conserved Plasmodium protein, unknown function	12	iCn3D view
PF3D7_0705500	Q8IC15	inositol-phosphate phosphatase, putative	45	iCn3D view
PF3D7_0707200	C0H4L9	conserved Plasmodium protein, unknown function	20	iCn3D view
PF3D7_0723800	Q8IBL5	apicomplexan kinetochore protein 1, putative	71	iCn3D view
PF3D7_0815800	Q8IAZ5	vacuolar protein sorting-associated protein 9, putative	51	iCn3D view
PF3D7_0827300	Q8IBA5	conserved Plasmodium protein, unknown function	6	iCn3D view
PF3D7_1013900	Q8IJQ6	translation initiation factor eIF-2B subunit delta, putative	9	iCn3D view
PF3D7_1021500	Q8IJI8	ATP-dependent RNA helicase ROK1, putative	3	iCn3D view
PF3D7_1122500	Q8IIE1	protein KIC10	80	iCn3D view
PF3D7_1135300	Q8II12	plasma membrane resident transporter 1	2	iCn3D view
PF3D7_1214100	Q8I5R4	GPI ethanolamine phosphate transferase 3, putative	3	iCn3D view
PF3D7_1230000	A0A144A0A3	TBC domain-containing protein, putative	6	iCn3D view
PF3D7_1235300	Q8I569	CCR4-NOT transcription complex subunit 4, putative	18	iCn3D view
PF3D7_1241900	Q8I510	tetratricopeptide repeat protein, putative	17	iCn3D view
PF3D7_1248700	Q8I4U7	conserved protein, unknown function	39	iCn3D view
PF3D7_1320900	C0H5C7	RNA-binding protein, putative	2	iCn3D view
PF3D7_1325900	A0A5K1K8R5	conserved Plasmodium protein, unknown function	13	iCn3D view
PF3D7_1334500	Q8IDY3	MSP7-like protein	9	iCn3D view

PF3D7_1335700	Q8IDX3	conserved oligomeric Golgi complex subunit 3, putative	13	iCn3D view
PF3D7_1346400	C0H5H6	VPS13 domain-containing protein, putative	70	iCn3D view
PF3D7_1348800	A0A5K1K8L6	E1-E2 ATPase, putative	17	iCn3D view
PF3D7_1358200	C0H5I8	conserved Plasmodium protein, unknown function	12	iCn3D view
PF3D7_1364400	A0A5K1K8L4	conserved Plasmodium protein, unknown function	27	iCn3D view
PF3D7_1428200	Q8ILI3	major facilitator superfamily domain-containing protein, putative	12	iCn3D view
PF3D7_1441300	Q8IL57	serine/threonine protein kinase, putative	32	iCn3D view
PF3D7_1447800	Q8IKZ7	calponin homology domain-containing protein, putative	30	iCn3D view
PF3D7_1452400	Q8IKV2	conserved Plasmodium protein, unknown function	9	iCn3D view
PF3D7_0103300	B9ZSI0	conserved protein, unknown function	4	iCn3D view
PF3D7_0206000	O96154	DNA repair protein RAD2, putative	8	iCn3D view
PF3D7_0214300	O96223	conserved Plasmodium protein, unknown function	10	iCn3D view
PF3D7_0214400	O96224	protein LTV1, putative	3	iCn3D view
PF3D7_0214800	O96228	conserved Plasmodium membrane protein, unknown function	12	iCn3D view
PF3D7_0216800	O96246	TMEM121 domain-containing protein, putative	5	iCn3D view
PF3D7_0306100	O97243	conserved Plasmodium protein, unknown function	9	iCn3D view
PF3D7_0310500	O77360	ATP-dependent RNA helicase DHX57, putative	15	iCn3D view
PF3D7_0312500	O77343	multidrug-resistant modulator MFR3	9	iCn3D view
PF3D7_0317200	O77385	cdc2-related protein kinase 4	26	iCn3D view
PF3D7_0317700	O77380	CPSF (cleavage and polyadenylation specific factor), subunit A, putative	25	iCn3D view
PF3D7_0321800	O97292	WD repeat-containing protein, putative	21	iCn3D view
PF3D7_0403600	C0H488	conserved Plasmodium protein, unknown function	6	iCn3D view
PF3D7_0416300	Q8I1S4	DNA helicase MCM9, putative	6	iCn3D view
PF3D7_0418300	Q8I1Q4	conserved Plasmodium protein, unknown function	6	iCn3D view
PF3D7_0504000	Q8I461	cation transporting P-ATPase	37	iCn3D view

PF3D7_0504700	Q8I455	centrosomal protein CEP120, putative	5	iCn3D view
PF3D7_0508500	Q8I418	ND6 protein, putative	8	iCn3D view
PF3D7_0508700	Q8I416	pre-mRNA-processing ATP-dependent RNA helicase PRP5, putative	20	iCn3D view
PF3D7_0510500	Q8I3Z9	topoisomerase I	6	iCn3D view
PF3D7_0516700	Q8I3U1	ubiquitin carboxyl-terminal hydrolase 2, putative	7	iCn3D view
PF3D7_0524100	Q8I3M4	conserved Plasmodium protein, unknown function	6	iCn3D view
PF3D7_0527200	Q8I3J3	ubiquitin carboxyl-terminal hydrolase 14	9	iCn3D view
PF3D7_0527600	C0H4G4	conserved Plasmodium protein, unknown function	11	iCn3D view
PF3D7_0529800	Q8I3H0	conserved Plasmodium protein, unknown function	49	iCn3D view
PF3D7_0605800	C6KSQ6	DNA repair protein RAD50, putative	6	iCn3D view
PF3D7_0613800	C6KSY0	AP2 domain transcription factor, putative	76	iCn3D view
PF3D7_0615400	C6KSZ5	ribonuclease, putative	55	iCn3D view
PF3D7_0619300	C6KT33	conserved Plasmodium protein, unknown function	14	iCn3D view
PF3D7_0624600	C6KT82	ISWI chromatin-remodeling complex ATPase	55	iCn3D view
PF3D7_0629700	C6KTD2	SET domain protein, putative	72	iCn3D view
PF3D7_0704100	C0H4K4	basal complex transmembrane protein 2	91	iCn3D view
PF3D7_0704400	Q8IC26	phosphoinositide-binding protein, putative	11	iCn3D view
PF3D7_0719900	Q8IBQ3	conserved Plasmodium membrane protein, unknown function	43	iCn3D view
PF3D7_0723400	Q8IBL9	conserved Plasmodium protein, unknown function	28	iCn3D view
PF3D7_0728600	Q8IBH2	RING zinc finger protein, putative	22	iCn3D view
PF3D7_0729100	C0H4Q1	apicomplexan kinetochore protein 8, putative	71	iCn3D view
PF3D7_0801700	C0H4Y4	sentrin-specific protease 2, putative	26	iCn3D view
PF3D7_0810600	Q8IAU1	ATP-dependent RNA helicase DBP1, putative	12	iCn3D view
PF3D7_0813000	A0A5K1K7Z9	protein KIC7	24	iCn3D view
PF3D7_0813500	Q8IAX1	PUB domain-containing protein, putative	17	iCn3D view

PF3D7_0818000	C0H4V5	U4/U6.U5 small nuclear ribonucleoprotein, putative	26	iCn3D view
PF3D7_0826100	Q8IB94	HECT-like E3 ubiquitin ligase, putative	48	iCn3D view
PF3D7_0832200	A0A146M1Y0	Plasmodium exported protein (PHISTa-like), unknown function	2	iCn3D view
PF3D7_0906600	Q8I383	zinc finger protein, putative	4	iCn3D view
PF3D7_0914500	Q8I307	conserved Plasmodium protein, unknown function	23	iCn3D view
PF3D7_0916700	Q8I2Y5	RNA-binding protein musashi, putative	23	iCn3D view
PF3D7_0924500	C0H557	conserved Plasmodium membrane protein, unknown function	10	iCn3D view
PF3D7_0925400	Q8I2Q4	protein phosphatase-beta	3	iCn3D view
PF3D7_0926100	Q8I2P8	protein kinase, putative	37	iCn3D view
PF3D7_0927600	Q8I2N3	RNA-binding protein, putative	22	iCn3D view
PF3D7_1001400	Q8IK22	exported lipase 1	1	iCn3D view
PF3D7_1004300	Q8IJZ4	RING finger E3 ubiquitin-protein ligase	30	iCn3D view
PF3D7_1010200	Q8IJU3	DNA2/NAM7 helicase, putative	16	iCn3D view
PF3D7_1011700	Q8IJS8	DNA repair protein RAD23, putative	5	iCn3D view
PF3D7_1014600	Q8IJP9	transcriptional coactivator ADA2	37	iCn3D view
PF3D7_1019000	Q8IJL2	eukaryotic translation initiation factor subunit eIF2A, putative	21	iCn3D view
PF3D7_1025900	A0A143ZYA5	conserved protein, unknown function	33	iCn3D view
PF3D7_1033600	Q7KQL1	pre-mRNA-splicing factor CEF1, putative	10	iCn3D view
PF3D7_1103800	Q8IIW4	CCR4-NOT transcription complex subunit NOT1-G, putative	19	iCn3D view
PF3D7_1107800	Q8IIS4	AP2 domain transcription factor, putative	63	iCn3D view
PF3D7_1112100	Q8IIP2	protein kinase, putative	25	iCn3D view
PF3D7_1116100	C6S3F2	serine esterase, putative	36	iCn3D view
PF3D7_1126800	Q8II95	alternative splicing factor SR-MG, putative	13	iCn3D view
PF3D7_1130600	Q8II58	tRNA:m(4)X modification enzyme TRM13, putative	3	iCn3D view
PF3D7_1133200	Q8II32	conserved Plasmodium protein, unknown function	31	iCn3D view

PF3D7_1136600	Q8IHZ8	conserved Plasmodium protein, unknown function	4	iCn3D view
PF3D7_1138000	Q8IHY4	conserved Plasmodium protein, unknown function	62	iCn3D view
PF3D7_1141300	Q8IHV2	apical polar ring protein APR1, putative	23	iCn3D view
PF3D7_1142100	Q8IHU4	DnaJ domain-containing protein, putative	71	iCn3D view
PF3D7_1149000	Q8IHN4	antigen 332, DBL-like protein	102	iCn3D view
PF3D7_1200700	Q8I638	acyl-CoA synthetase	10	iCn3D view
PF3D7_1203400	Q8I611	major facilitator superfamily domain-containing protein, putative	16	iCn3D view
PF3D7_1206300	A0A143ZZM5	conserved Plasmodium protein, unknown function	40	iCn3D view
PF3D7_1207000	Q8I5X5	conserved Plasmodium protein, unknown function	89	iCn3D view
PF3D7_1210600	Q8I5U3	kelch protein, putative	25	iCn3D view
PF3D7_1212700	Q8I5S6	eukaryotic translation initiation factor 3 subunit A, putative	30	iCn3D view
PF3D7_1219000	Q8I5L7	formin 2	35	iCn3D view
PF3D7_1219600	Q8I5L4	phospholipid-transporting ATPase 2	24	iCn3D view
PF3D7_1220300	Q8I5L0	cell cycle associated protein, putative	27	iCn3D view
PF3D7_1224000	Q8I5H7	GTP cyclohydrolase 1	12	iCn3D view
PF3D7_1224700	Q8I5H0	conserved Plasmodium protein, unknown function	22	iCn3D view
PF3D7_1225400	Q8I5G3	rRNA-processing protein, putative	1	iCn3D view
PF3D7_1229100	Q8I5C7	multidrug resistance-associated protein 2	29	iCn3D view
PF3D7_1241800	Q8I511	ATP-dependent RNA helicase DBP9, putative	5	iCn3D view
PF3D7_1249300	Q8I4U1	protein phosphatase PPM4, putative	23	iCn3D view
PF3D7_1249800	Q8I4T6	THO complex subunit 2, putative	17	iCn3D view
PF3D7_1252100	Q8I4R5	rhoptry neck protein 3	10	iCn3D view
PF3D7_1303500	Q8IET0	sodium/hydrogen exchanger	50	iCn3D view
PF3D7_1304400	Q8IES1	PUB domain-containing protein, putative	6	iCn3D view
PF3D7_1306500	C0H5A3	MORN repeat protein, putative	7	iCn3D view

PF3D7_1312900	C0H5B1	eukaryotic translation initation factor 4 gamma	25	iCn3D view
PF3D7_1327300	A0A5K1K8E0	subpellicular microtubule protein 3	74	iCn3D view
PF3D7_1328000	A0A5K1K950	conserved Plasmodium protein, unknown function	28	iCn3D view
PF3D7_1333600	Q8IDZ2	U3 small nucleolar RNA-associated protein 4, putative	11	iCn3D view
PF3D7_1338700	A0A5K1K8M0	conserved protein, unknown function	16	iCn3D view
PF3D7_1345400	A0A5K1K9F6	conserved Plasmodium protein, unknown function	37	iCn3D view
PF3D7_1356800	Q8IDD4	serine/threonine protein kinase ARK3, putative	56	iCn3D view
PF3D7_1365800	A0A5K1K961	conserved Plasmodium protein, unknown function	7	iCn3D view
PF3D7_1366300	A0A5K1K933	conserved Plasmodium protein, unknown function	36	iCn3D view
PF3D7_1369400	C0H5L6	conserved Plasmodium protein, unknown function	4	iCn3D view
PF3D7_1408200	Q8IM14	AP2 domain transcription factor AP2-G2	10	iCn3D view
PF3D7_1414500	A0A144A1E4	atypical protein kinase, ABC-1 family, putative	45	iCn3D view
PF3D7_1416200	Q8ILT4	metacaspase-3	35	iCn3D view
PF3D7_1420600	Q8ILP4	pantothenate kinase 1	1	iCn3D view
PF3D7_1430300	Q8ILG1	acid phosphatase, putative	26	iCn3D view
PF3D7_1436200	Q8ILA2	basal complex protein BCP1	59	iCn3D view
PF3D7_1439100	Q8IL78	DEAD/DEAH box helicase, putative	3	iCn3D view
PF3D7_1451200	Q8IKW4	conserved Plasmodium protein, unknown function	30	iCn3D view
PF3D7_1453800	Q8IKU0	glucose-6-phosphate dehydrogenase-6-phosphogluconolactonase	14	iCn3D view
PF3D7_1455300	Q8IKS5	conserved protein, unknown function	25	iCn3D view
PF3D7_1456000	Q8IKR9	AP2 domain transcription factor AP2-HC	12	iCn3D view
PF3D7_1457700	Q8IKQ3	large ribosomal subunit nuclear export factor, putative	4	iCn3D view
PF3D7_1464500	Q8IKJ2	conserved Plasmodium membrane protein, unknown function	103	iCn3D view
PF3D7_1466200	Q8IKH4	homeodomain-like protein HDP1	4	iCn3D view
PF3D7_1468800	Q8IKE9	splicing factor U2AF large subunit, putative	30	iCn3D view

PF3D7_1476300	Q8IK74	Plasmodium exported protein (PHISTb), unknown function	9	iCn3D view
PF3D7_0102200	Q8I0U6	ring-infected erythrocyte surface antigen	15	iCn3D view
PF3D7_0102500	Q8I2B4	erythrocyte binding antigen-181	5	iCn3D view
PF3D7_0102600	B9ZSH8	serine/threonine protein kinase, FIKK family	2	iCn3D view
PF3D7_0102800	Q8I2B2	seipin domain-containing protein, putative	2	iCn3D view
PF3D7_0102900	Q8I2B1	aspartate--tRNA ligase	1	iCn3D view
PF3D7_0103000	B9ZSH9	vacuolar protein sorting-associated protein VTA1, putative	2	iCn3D view
PF3D7_0103200	Q8I2A8	nucleoside transporter 4	6	iCn3D view
PF3D7_0103500	B9ZSI1	conserved Plasmodium protein, unknown function	1	iCn3D view
PF3D7_0103900	Q8I2A1	parasite-infected erythrocyte surface protein	1	iCn3D view
PF3D7_0104100	Q8I299	protein E140, putative	1	iCn3D view
PF3D7_0104500	Q8I294	conserved protein, unknown function	1	iCn3D view
PF3D7_0104800	Q8I291	novel putative transporter 1, putative	14	iCn3D view
PF3D7_0105200	Q8I289	RAP protein RAP1	1	iCn3D view
PF3D7_0105600	Q8I285	FtsJ-like methyltransferase, putative	5	iCn3D view
PF3D7_0105700	Q8I284	asparagine-rich antigen Pfa35-2	27	iCn3D view
PF3D7_0105800	Q8I283	cyclin-dependent kinases regulatory subunit, putative	29	iCn3D view
PF3D7_0106100	Q8I280	V-type proton ATPase subunit C, putative	7	iCn3D view
PF3D7_0106300	Q76NN8	calcium-transporting ATPase	22	iCn3D view
PF3D7_0106400	Q8I278	pre-rRNA-processing protein TSR2, putative	1	iCn3D view
PF3D7_0106500	Q8I277	conserved Plasmodium protein, unknown function	6	iCn3D view
PF3D7_0106700	Q8I275	small ribosomal subunit assembling AARP2 protein	8	iCn3D view
PF3D7_0106800	Q8I274	ras-related protein Rab-5C	2	iCn3D view
PF3D7_0107000	Q8I272	centrin-1	2	iCn3D view
PF3D7_0107500	Q8I266	Niemann-Pick type C1-related protein	19	iCn3D view

PF3D7_0107800	A0A143ZUM0	double-strand break repair protein MRE11	7	iCn3D view
PF3D7_0108300	Q8I259	conserved Plasmodium protein, unknown function	50	iCn3D view
PF3D7_0108500	Q8I257	ELM2 domain-containing protein, putative	3	iCn3D view
PF3D7_0108700	Q8I255	secreted ookinete protein, putative	24	iCn3D view
PF3D7_0108800	Q8I254	mitochondrial carrier protein, putative	2	iCn3D view
PF3D7_0109000	Q8I253	photosensitized INA-labeled protein PHIL1	16	iCn3D view
PF3D7_0109400	Q8I250	tubulin-specific chaperone a, putative	2	iCn3D view
PF3D7_0109500	Q8I249	N-acetyltransferase, GNAT family, putative	1	iCn3D view
PF3D7_0109600	Q8I248	cold-shock protein, putative	2	iCn3D view
PF3D7_0109800	Q8I246	phenylalanine--tRNA ligase alpha subunit	1	iCn3D view
PF3D7_0110400	Q8I241	DNA-directed RNA polymerase II subunit RPB9, putative	6	iCn3D view
PF3D7_0110500	Q8I240	bromodomain protein 3, putative	26	iCn3D view
PF3D7_0110600	Q8I239	phosphatidylinositol-4-phosphate 5-kinase	37	iCn3D view
PF3D7_0110700	Q8I238	chromatin assembly factor 1 subunit C, putative	14	iCn3D view
PF3D7_0111000	Q8I235	kinesin-8B, putative	7	iCn3D view
PF3D7_0111400	B9ZSI9	conserved Plasmodium protein, unknown function	12	iCn3D view
PF3D7_0111500	Q8I231	UMP-CMP kinase, putative	3	iCn3D view
PF3D7_0111600	Q8I230	conserved Plasmodium protein, unknown function	1	iCn3D view
PF3D7_0111800	B9ZSJ0	eukaryotic translation initiation factor 4E, putative	5	iCn3D view
PF3D7_0111900	Q8I227	mitochondrial distribution and morphology protein, putative	1	iCn3D view
PF3D7_0112200	Q9U0N4	multidrug resistance-associated protein 1	38	iCn3D view
PF3D7_0113900	Q8I2D9	CX3CL1-binding protein 1	1	iCn3D view
PF3D7_0114000	B9ZSJ2	exported protein family 1	8	iCn3D view
PF3D7_0114100	B9ZSJ3	Pfmc-2TM Maurer's cleft two transmembrane protein	4	iCn3D view
PF3D7_0114500	Q8I2D5	Plasmodium exported protein (hyp10), unknown function	1	iCn3D view

PF3D7_0115000	Q8I2D1	surface-associated interspersed protein 1.3 (SURFIN 1.3)	1	iCn3D view
PF3D7_0201800	O96123	knob associated heat shock protein 40	3	iCn3D view
PF3D7_0202100	O96125	liver stage associated protein 2	1	iCn3D view
PF3D7_0202200	Q8I669	EMP1-trafficking protein	4	iCn3D view
PF3D7_0202400	O96127	translation-enhancing factor	25	iCn3D view
PF3D7_0202500	O96128	early transcribed membrane protein 2	5	iCn3D view
PF3D7_0202600	O96129	nucleic acid-binding protein, putative	19	iCn3D view
PF3D7_0203000	O96133	repetitive organellar protein, putative	16	iCn3D view
PF3D7_0203100	O96134	protein kinase, putative	13	iCn3D view
PF3D7_0203200	C6S3A8	conserved Plasmodium protein, unknown function	4	iCn3D view
PF3D7_0203300	O96136	ERCC1 nucleotide excision repair protein, putative	1	iCn3D view
PF3D7_0203400	Q8I668	conserved protein, unknown function	6	iCn3D view
PF3D7_0203600	O96137	conserved Plasmodium protein, unknown function	7	iCn3D view
PF3D7_0203700	A0A143ZWE8	protein MAK16, putative	2	iCn3D view
PF3D7_0204200	Q8I665	translocation protein SEC66, putative	5	iCn3D view
PF3D7_0204500	O96142	aspartate transaminase	1	iCn3D view
PF3D7_0204700	Q7KWJ5	hexose transporter	4	iCn3D view
PF3D7_0205100	C6S3B0	conserved Plasmodium protein, unknown function	4	iCn3D view
PF3D7_0205800	C6S3B1	PH domain-containing protein, putative	1	iCn3D view
PF3D7_0205900	O96153	26S proteasome regulatory subunit RPN1, putative	5	iCn3D view
PF3D7_0206200	O96156	pantothenate transporter	7	iCn3D view
PF3D7_0206700	Q7KWJ4	adenylosuccinate lyase	2	iCn3D view
PF3D7_0206800	P50498	merozoite surface protein 2	6	iCn3D view
PF3D7_0207000	Q7KWJ2	merozoite surface protein 4	1	iCn3D view
PF3D7_0207100	O96160	conserved Plasmodium protein, unknown function	5	iCn3D view

PF3D7_0207400	O96163	serine repeat antigen 7	2	iCn3D view
PF3D7_0207500	Q9TY96	serine repeat antigen 6	2	iCn3D view
PF3D7_0207600	Q9TY95	serine repeat antigen 5	10	iCn3D view
PF3D7_0208100	O96168	C2 domain-containing protein 5, putative	33	iCn3D view
PF3D7_0208200	O96169	KRR1 small subunit processome component, putative	1	iCn3D view
PF3D7_0208400	O96171	histidine phosphatase, putative	10	iCn3D view
PF3D7_0208700	Q8I662	SHS2 domain-containing protein, putative	2	iCn3D view
PF3D7_0208900	O96175	6-cysteine protein P230p	1	iCn3D view
PF3D7_0209000	P68874	6-cysteine protein P230	5	iCn3D view
PF3D7_0209100	O96176	patatin-like phospholipase 1	14	iCn3D view
PF3D7_0209200	O96177	exosome complex component MTR3, putative	1	iCn3D view
PF3D7_0209600	O96181	transporter, putative	10	iCn3D view
PF3D7_0209800	Q9TY94	ATP-dependent RNA helicase UAP56	16	iCn3D view
PF3D7_0210100	O96184	60S ribosomal protein L37ae, putative	1	iCn3D view
PF3D7_0210200	O96185	conserved Plasmodium protein, unknown function	9	iCn3D view
PF3D7_0210300	O96186	monocarboxylate transporter, putative	3	iCn3D view
PF3D7_0210600	O96188	protein CERLI1	9	iCn3D view
PF3D7_0210700	O96189	syntaxin, Qa-SNARE family	2	iCn3D view
PF3D7_0210900	O96191	conserved Plasmodium protein, unknown function	25	iCn3D view
PF3D7_0211200	O96193	ras-related protein Rab-5A	1	iCn3D view
PF3D7_0211700	O96197	tyrosine kinase-like protein, putative	4	iCn3D view
PF3D7_0211800	O96198	asparagine--tRNA ligase	3	iCn3D view
PF3D7_0212100	O96201	conserved Plasmodium protein, unknown function	21	iCn3D view
PF3D7_0212300	O96203	eukaryotic peptide chain release factor subunit 1, putative	9	iCn3D view
PF3D7_0212400	O96204	nucleoporin NUP390, putative	15	iCn3D view

PF3D7_0212700	O96208	SRR1-like protein	1	iCn3D view
PF3D7_0212800	O96209	multidrug efflux pump, putative	4	iCn3D view
PF3D7_0212900	O96210	arginyl-tRNA--protein transferase	5	iCn3D view
PF3D7_0213100	O96212	protein SIS1	3	iCn3D view
PF3D7_0213500	O96215	tetratricopeptide repeat protein, putative	4	iCn3D view
PF3D7_0213700	O96217	conserved protein, unknown function	2	iCn3D view
PF3D7_0214600	O96226	serine/threonine protein kinase STK2, putative	2	iCn3D view
PF3D7_0214700	O96227	conserved Plasmodium protein, unknown function	7	iCn3D view
PF3D7_0214900	O96229	rhoptry neck protein 6	2	iCn3D view
PF3D7_0215000	O96230	acyl-CoA synthetase	1	iCn3D view
PF3D7_0215100	Q8I660	RING zinc finger protein, putative	4	iCn3D view
PF3D7_0215300	O96232	acyl-CoA synthetase	3	iCn3D view
PF3D7_0215400	O96233	WD repeat-containing protein, putative	8	iCn3D view
PF3D7_0215500	O96234	conserved Plasmodium protein, unknown function	2	iCn3D view
PF3D7_0215700	O96236	DNA-directed RNA polymerase II subunit RPB2, putative	1	iCn3D view
PF3D7_0215800	O96237	origin recognition complex subunit 5	7	iCn3D view
PF3D7_0216000	O96239	DEAD/DEAH box helicase, putative	3	iCn3D view
PF3D7_0216400	O96243	vacuolar protein sorting-associated protein 45	4	iCn3D view
PF3D7_0216600	O96245	sugar transporter, putative	2	iCn3D view
PF3D7_0216700	Q8I659	autophagy-related protein 11, putative	10	iCn3D view
PF3D7_0216900	O96248	conserved Plasmodium protein, unknown function	1	iCn3D view
PF3D7_0217500	P62344	calcium-dependent protein kinase 1	16	iCn3D view
PF3D7_0217700	Q8I657	E2F-associated phosphoprotein, putative	1	iCn3D view
PF3D7_0217800	O96258	40S ribosomal protein S26	4	iCn3D view
PF3D7_0217900	O96259	thioesterase/thiol ester dehydrase-isomerase, putative	2	iCn3D view

PF3D7_0218100	O96261	conserved protein, unknown function	1	iCn3D view
PF3D7_0218200	C6S3B8	SUZ domain-containing protein, putative	6	iCn3D view
PF3D7_0218400	O96264	ATP-dependent RNA helicase DDX47, putative	1	iCn3D view
PF3D7_0218500	O96265	small nuclear ribonucleoprotein Sm D2, putative	2	iCn3D view
PF3D7_0218600	O96266	patatin-like phospholipase, putative	16	iCn3D view
PF3D7_0218700	O96267	pre-mRNA-processing protein 45, putative	7	iCn3D view
PF3D7_0219200	O96269	40S ribosomal protein S30	1	iCn3D view
PF3D7_0219400	Q8I655	ribosome associated membrane protein RAMP4, putative	3	iCn3D view
PF3D7_0219600	O96271	replication factor C subunit 1	28	iCn3D view
PF3D7_0219700	O96272	Plasmodium exported protein (PHISTc), unknown function	1	iCn3D view
PF3D7_0220000	O96275	liver stage antigen 3	11	iCn3D view
PF3D7_0220100	O96276	DnaJ protein, putative	1	iCn3D view
PF3D7_0220200	Q7KWI7	Plasmodium exported protein, unknown function	1	iCn3D view
PF3D7_0220600	O96278	Plasmodium exported protein (hyp9), unknown function	4	iCn3D view
PF3D7_0220800	O96279	cytoadherence linked asexual protein 2	3	iCn3D view
PF3D7_0221200	Q8I652	Plasmodium exported protein (hyp15), unknown function	3	iCn3D view
PF3D7_0301200	C0H466	serine/threonine protein kinase, FIKK family	1	iCn3D view
PF3D7_0301300	Q9Y013	epoxide hydrolase 1	2	iCn3D view
PF3D7_0301600	C0H468	Plasmodium exported protein (hyp1), unknown function	4	iCn3D view
PF3D7_0301700	O97336	EMP1 trafficking protein PTP7	3	iCn3D view
PF3D7_0301800	O97332	Plasmodium exported protein, unknown function	1	iCn3D view
PF3D7_0302000	O97334	pre-mRNA-splicing factor PRP46, putative	2	iCn3D view
PF3D7_0302100	O77306	serine/threonine protein kinase	27	iCn3D view
PF3D7_0302200	O77309	cytoadherence linked asexual protein 3.2	13	iCn3D view
PF3D7_0302600	O77308	ABC transporter B family member 4, putative	1	iCn3D view

PF3D7_0302800	O77316	RNA-binding protein, putative	1	iCn3D view
PF3D7_0302900	O77312	exportin-1, putative	8	iCn3D view
PF3D7_0303000	O77313	N-ethylmaleimide-sensitive fusion protein	8	iCn3D view
PF3D7_0303200	O77317	lipin, putative	44	iCn3D view
PF3D7_0303300	O77315	DNA-directed RNA polymerases I, II, and III subunit RPABC2, putative	3	iCn3D view
PF3D7_0303400	O97224	palmitoyltransferase DHHC1	4	iCn3D view
PF3D7_0303500	O97225	spindle pole body protein, putative	23	iCn3D view
PF3D7_0303700	O97227	lipoamide acyltransferase component of branched-chain alpha-keto acid dehydrogenase complex	1	iCn3D view
PF3D7_0303800	O97226	IBR domain protein, putative	2	iCn3D view
PF3D7_0304200	Q9NLB8	EH domain-containing protein	4	iCn3D view
PF3D7_0304300	O97230	conserved Plasmodium protein, unknown function	1	iCn3D view
PF3D7_0304400	O97231	60S ribosomal protein L44	1	iCn3D view
PF3D7_0304800	O97234	conserved Plasmodium membrane protein, unknown function	3	iCn3D view
PF3D7_0305300	O97238	transporter, putative	17	iCn3D view
PF3D7_0305500	O97239	protein dopey homolog, putative	55	iCn3D view
PF3D7_0305700	O97241	ubiquitin-conjugating enzyme MMS2, putative	3	iCn3D view
PF3D7_0306200	O97244	activator of Hsp90 ATPase	2	iCn3D view
PF3D7_0306700	Q9NLB0	ER membrane protein complex subunit 5, putative	1	iCn3D view
PF3D7_0306800	O97247	T-complex protein 1 subunit beta	6	iCn3D view
PF3D7_0306900	O97248	40S ribosomal protein S23, putative	5	iCn3D view
PF3D7_0307100	O97249	40S ribosomal protein S12, putative	5	iCn3D view
PF3D7_0307200	O97250	60S ribosomal protein L7, putative	1	iCn3D view
PF3D7_0307300	O97251	end-binding protein 1	4	iCn3D view
PF3D7_0307500	O97253	spindle and kinetochore-associated protein 2, putative	4	iCn3D view
PF3D7_0307600	O97254	conserved Plasmodium protein, unknown function	3	iCn3D view

PF3D7_0307900	O77320	conserved Plasmodium protein, unknown function	6	iCn3D view
PF3D7_0308000	O77321	DNA polymerase delta small subunit, putative	1	iCn3D view
PF3D7_0308200	O77323	T-complex protein 1 subunit eta	3	iCn3D view
PF3D7_0308500	O97256	activator of Hsp90 ATPase, putative	2	iCn3D view
PF3D7_0308600	O77325	pre-mRNA-processing factor 19, putative	3	iCn3D view
PF3D7_0308700	O77326	conserved protein, unknown function	6	iCn3D view
PF3D7_0308900	O77327	splicing factor 3B subunit 1, putative	15	iCn3D view
PF3D7_0309200	O77328	serine/threonine protein kinase ARK2, putative	15	iCn3D view
PF3D7_0309300	O77329	N2227-like protein, putative	6	iCn3D view
PF3D7_0309500	O77330	asparagine synthetase [glutamine-hydrolyzing], putative	8	iCn3D view
PF3D7_0309600	O00806	60S acidic ribosomal protein P2	1	iCn3D view
PF3D7_0310100	Q9NJU9	calcium-dependent protein kinase 3	1	iCn3D view
PF3D7_0310300	C0H469	phosphoglycerate mutase, putative	16	iCn3D view
PF3D7_0310400	O77361	parasite-infected erythrocyte surface protein	2	iCn3D view
PF3D7_0310700	O77358	trafficking protein particle complex subunit 4, putative	1	iCn3D view
PF3D7_0310800	O77359	conserved Plasmodium protein, unknown function	1	iCn3D view
PF3D7_0310900	O77357	conserved Plasmodium protein, unknown function	27	iCn3D view
PF3D7_0311100	O77355	pre-mRNA splicing factor, putative	15	iCn3D view
PF3D7_0311400	O77365	kinase-related protein PKRP, putative	4	iCn3D view
PF3D7_0312300	O77345	26S proteasome regulatory subunit RPN12, putative	1	iCn3D view
PF3D7_0312400	O77344	glycogen synthase kinase-3 beta	5	iCn3D view
PF3D7_0312800	O77364	60S ribosomal protein L26, putative	2	iCn3D view
PF3D7_0313400	O77339	conserved Plasmodium protein, unknown function	3	iCn3D view
PF3D7_0313600	O77337	conserved Plasmodium protein, unknown function	3	iCn3D view
PF3D7_0314100	A0A143ZY06	vesicle transport v-SNARE protein, putative	5	iCn3D view

PF3D7_0314300	O97275	DER1-like protein	1	iCn3D view
PF3D7_0314400	O97259	serine/threonine protein phosphatase 6, putative	1	iCn3D view
PF3D7_0314600	Q9Y011	conserved protein, unknown function	2	iCn3D view
PF3D7_0314700	O97260	RING finger protein RNF1	8	iCn3D view
PF3D7_0314800	C0H475	conserved Plasmodium protein, unknown function	10	iCn3D view
PF3D7_0314900	O97264	conserved Plasmodium membrane protein, unknown function	2	iCn3D view
PF3D7_0315000	O97265	zinc finger protein, putative	1	iCn3D view
PF3D7_0315100	O97266	eukaryotic translation initiation factor 4E	3	iCn3D view
PF3D7_0315600	O97271	male development protein MD3, putative	5	iCn3D view
PF3D7_0315700	O97272	conserved Plasmodium membrane protein, unknown function	1	iCn3D view
PF3D7_0315800	O97273	zinc finger protein, putative	2	iCn3D view
PF3D7_0316200	O77393	conserved Plasmodium protein, unknown function	3	iCn3D view
PF3D7_0316300	O77392	inorganic pyrophosphatase, putative	2	iCn3D view
PF3D7_0316500	O77390	kinetochore protein NUF2, putative	3	iCn3D view
PF3D7_0316600	O77389	formate-nitrite transporter	3	iCn3D view
PF3D7_0316700	O77388	protein YOP1, putative	5	iCn3D view
PF3D7_0316800	O77395	40S ribosomal protein S15A, putative	3	iCn3D view
PF3D7_0316900	O77387	E3 ubiquitin-protein ligase, putative	6	iCn3D view
PF3D7_0317300	O77384	nucleoporin NUP335, putative	43	iCn3D view
PF3D7_0317500	O77382	kinesin-5	7	iCn3D view
PF3D7_0317600	O77381	40S ribosomal protein S11, putative	3	iCn3D view
PF3D7_0317800	O77379	26S proteasome non-ATPase regulatory subunit 9, putative	2	iCn3D view
PF3D7_0318200	O77375	DNA-directed RNA polymerase II subunit RPB1	38	iCn3D view
PF3D7_0318300	O77374	conserved Plasmodium protein, unknown function	1	iCn3D view
PF3D7_0318500	O77372	conserved Plasmodium protein, unknown function	25	iCn3D view

PF3D7_0318600	O77371	cleavage and polyadenylation specificity factor, putative	1	iCn3D view
PF3D7_0318700	Q9NDU5	conserved Plasmodium protein, unknown function	6	iCn3D view
PF3D7_0319000	O77368	P4-type ATPase ATP7, putative	16	iCn3D view
PF3D7_0319100	O77367	E3 ubiquitin-protein ligase RBX1, putative	1	iCn3D view
PF3D7_0319200	O77366	CCR4 domain-containing protein 4, putative	1	iCn3D view
PF3D7_0319400	O97277	kinesin-8X	9	iCn3D view
PF3D7_0319500	O97318	RNA-binding protein, putative	8	iCn3D view
PF3D7_0319600	O97319	elongation factor 1-delta, putative	3	iCn3D view
PF3D7_0319700	O97278	ABC transporter I family member 1, putative	18	iCn3D view
PF3D7_0319900	O97279	conserved protein, unknown function	3	iCn3D view
PF3D7_0320000	C0H480	protein phosphatase inhibitor 2	10	iCn3D view
PF3D7_0320100	O97323	protein transport protein SEC22	1	iCn3D view
PF3D7_0320400	O97283	oocyst capsule protein Cap380	1	iCn3D view
PF3D7_0320700	Q9NFA0	signal peptidase complex subunit 2	1	iCn3D view
PF3D7_0320800	O97285	ATP-dependent RNA helicase DDX6	3	iCn3D view
PF3D7_0320900	O97320	histone H2A.Z	4	iCn3D view
PF3D7_0321100	O97287	conserved Plasmodium protein, unknown function	24	iCn3D view
PF3D7_0321500	O97289	peptidase, putative	5	iCn3D view
PF3D7_0321600	O97290	ATP-dependent RNA helicase DDX42, putative	9	iCn3D view
PF3D7_0321700	O97291	conserved Plasmodium protein, unknown function	11	iCn3D view
PF3D7_0321900	C0H483	cyclic amine resistance locus protein	9	iCn3D view
PF3D7_0322000	Q76NN7	peptidyl-prolyl cis-trans isomerase	5	iCn3D view
PF3D7_0322100	C0H484	mRNA-capping enzyme subunit beta	3	iCn3D view
PF3D7_0322300	O97295	diacylglycerol O-acyltransferase	1	iCn3D view
PF3D7_0322400	C0H485	regulator of initiation factor 2 (eIF2)	4	iCn3D view

PF3D7_0322600	O97322	conserved Plasmodium protein, unknown function	5	iCn3D view
PF3D7_0322700	O97298	conserved Plasmodium protein, unknown function	1	iCn3D view
PF3D7_0322900	O97313	40S ribosomal protein S3A, putative	12	iCn3D view
PF3D7_0323000	O97314	translation machinery-associated protein 7, putative	1	iCn3D view
PF3D7_0323100	O97299	conserved Plasmodium protein, unknown function	19	iCn3D view
PF3D7_0323500	O97304	survival motor neuron-like protein	2	iCn3D view
PF3D7_0323600	O97305	BSD domain-containing protein, putative	3	iCn3D view
PF3D7_0323800	O97306	conserved Plasmodium protein, unknown function	11	iCn3D view
PF3D7_0324100	O97307	Pfmc-2TM Maurer's cleft two transmembrane protein	3	iCn3D view
PF3D7_0401600	Q8I209	rifin	1	iCn3D view
PF3D7_0401900	Q8I0V8	acyl-CoA synthetase	1	iCn3D view
PF3D7_0402000	Q8I206	Plasmodium exported protein (PHISTa), unknown function	6	iCn3D view
PF3D7_0402100	Q8I205	Plasmodium exported protein (PHISTb), unknown function	10	iCn3D view
PF3D7_0402300	P86148	reticulocyte binding protein homologue 1	1	iCn3D view
PF3D7_0402400	Q8I202	Plasmodium exported protein, unknown function	6	iCn3D view
PF3D7_0402900	Q8I200	probable protein, unknown function	1	iCn3D view
PF3D7_0403400	C0H487	zinc finger protein, putative	3	iCn3D view
PF3D7_0403700	Q8I1Z2	pre-mRNA-splicing factor CLF1, putative	8	iCn3D view
PF3D7_0403800	Q8I1Z1	alpha/beta hydrolase, putative	14	iCn3D view
PF3D7_0403900	Q8I1Z0	SET domain protein, putative	9	iCn3D view
PF3D7_0404000	Q8I1Y9	conserved Plasmodium protein, unknown function	1	iCn3D view
PF3D7_0404100	Q8I1Y8	AP2 domain transcription factor AP2-SP2, putative	2	iCn3D view
PF3D7_0404600	Q8I1Y3	conserved Plasmodium membrane protein, unknown function	13	iCn3D view
PF3D7_0404700	Q8I1Y2	dipeptidyl aminopeptidase 3	1	iCn3D view
PF3D7_0404900	Q8I1Y0	6-cysteine protein P41	1	iCn3D view

PF3D7_0405100	C0H489	protein transport protein Sec24B, putative	6	iCn3D view
PF3D7_0405200	Q8I1X7	protein CERLI2	3	iCn3D view
PF3D7_0405400	Q8I1X5	pre-mRNA-processing-splicing factor 8, putative	19	iCn3D view
PF3D7_0405600	Q8I1X3	TMEM33 domain-containing protein, putative	1	iCn3D view
PF3D7_0405700	Q8I1X1	lysine decarboxylase, putative	12	iCn3D view
PF3D7_0405900	Q8I1W9	apical sushi protein	1	iCn3D view
PF3D7_0406000	Q8I1W8	conserved protein, unknown function	4	iCn3D view
PF3D7_0406100	Q6ZMA8	V-type proton ATPase subunit B	8	iCn3D view
PF3D7_0406200	Q6ZMA7	parasitophorous vacuole membrane protein S16	6	iCn3D view
PF3D7_0406400	C0H490	cytosolic glyoxalase II	1	iCn3D view
PF3D7_0406500	C0H491	NYN domain-containing protein, putative	14	iCn3D view
PF3D7_0406700	C0H492	KLRAQ domain-containing protein, putative	1	iCn3D view
PF3D7_0407200	Q9U0L4	peptidyl-tRNA hydrolase 2, putative	1	iCn3D view
PF3D7_0407300	Q9U0L3	transcription factor, putative	3	iCn3D view
PF3D7_0407700	Q9U0K9	TH1 domain-containing protein, putative	20	iCn3D view
PF3D7_0407900	Q9U0K7	AAA family ATPase, putative	4	iCn3D view
PF3D7_0408100	Q9U0K5	conserved Plasmodium protein, unknown function	4	iCn3D view
PF3D7_0408200	Q9U0K4	conserved Plasmodium protein, unknown function	3	iCn3D view
PF3D7_0408300	Q9U0K3	zinc finger Ran-binding domain-containing protein 2, putative	13	iCn3D view
PF3D7_0408500	Q7K734	flap endonuclease 1	16	iCn3D view
PF3D7_0408700	Q9U0J9	perforin-like protein 1	1	iCn3D view
PF3D7_0409100	C0H4A0	U4/U6 small nuclear ribonucleoprotein PRP31, putative	2	iCn3D view
PF3D7_0409200	Q9U0J4	protein SOF1, putative	1	iCn3D view
PF3D7_0409300	Q9U0J3	methyltransferase, putative	2	iCn3D view
PF3D7_0409400	Q9U0J2	chaperone protein DnaJ	1	iCn3D view

PF3D7_0409600	Q9U0J0	replication protein A1, large subunit	18	iCn3D view
PF3D7_0409800	Q9U0I8	zinc finger protein, putative	6	iCn3D view
PF3D7_0410300	Q9U0I5	protein phosphatase PPM1, putative	4	iCn3D view
PF3D7_0410500	Q9U0I3	conserved Plasmodium protein, unknown function	1	iCn3D view
PF3D7_0410600	Q9U0I2	armadillo-type repeat protein ATRP	4	iCn3D view
PF3D7_0410800	Q9U0I0	apicomplexan kinetochore protein 3, putative	14	iCn3D view
PF3D7_0410900	Q9U0H9	apicomplexan kinetochore protein 7, putative	11	iCn3D view
PF3D7_0411000	Q9U0H8	AP2 domain transcription factor AP2-Z, putative	1	iCn3D view
PF3D7_0411100	Q9U0H7	mediator of RNA polymerase II transcription subunit 8, putative	1	iCn3D view
PF3D7_0411800	Q9U0H2	conserved Plasmodium protein, unknown function	8	iCn3D view
PF3D7_0411900	Q9U0H1	DNA polymerase alpha catalytic subunit A	13	iCn3D view
PF3D7_0412000	Q9U0H0	LITAF-like zinc finger protein, putative	1	iCn3D view
PF3D7_0413600	Q8I1V1	26S protease regulatory subunit 6B, putative	4	iCn3D view
PF3D7_0413700	Q8I1V0	lysine decarboxylase-like protein, putative	4	iCn3D view
PF3D7_0413900	Q8I1U8	ubiquitin carboxyl-terminal hydrolase 13, putative	12	iCn3D view
PF3D7_0414000	Q8I1U7	structural maintenance of chromosomes protein 3	1	iCn3D view
PF3D7_0414600	Q8I1U2	acylated pleckstrin-homology domain-containing protein, putative	2	iCn3D view
PF3D7_0414900	C0H4A5	armadillo-domain containing rhoptry protein	9	iCn3D view
PF3D7_0415000	Q8I1T8	ATPase GET3	3	iCn3D view
PF3D7_0415300	Q8I1T4	cdc2-related protein kinase 3	19	iCn3D view
PF3D7_0415400	Q8I1T3	coatomer subunit zeta, putative	5	iCn3D view
PF3D7_0415500	Q8I1T2	nuclear cap-binding protein subunit 2, putative	3	iCn3D view
PF3D7_0415800	Q8I1S9	PhIL1-interacting candidate PIC3	11	iCn3D view
PF3D7_0415900	C0H4A6	60S ribosomal protein L15, putative	3	iCn3D view
PF3D7_0416000	Q8I1S7	RNA-binding protein, putative	13	iCn3D view

PF3D7_0416200	Q8I1S5	PRKR-interacting protein 1, putative	6	iCn3D view
PF3D7_0416400	C0H4A7	histone acetyltransferase, putative	29	iCn3D view
PF3D7_0416500	Q8I1S2	repressor of RNA polymerase III transcription MAF1	3	iCn3D view
PF3D7_0416800	Q8I1S0	small GTP-binding protein sar1	8	iCn3D view
PF3D7_0416900	C0H4A9	conserved Plasmodium protein, unknown function	6	iCn3D view
PF3D7_0417300	Q8I1R5	LETM1-like protein, putative	2	iCn3D view
PF3D7_0417400	C0H4B0	conserved Plasmodium protein, unknown function	17	iCn3D view
PF3D7_0417600	Q8I1R1	conserved Plasmodium protein, unknown function	7	iCn3D view
PF3D7_0417800	Q8I1Q9	cdc2-related protein kinase 1	8	iCn3D view
PF3D7_0418000	C0H4B3	conserved Plasmodium protein, unknown function	1	iCn3D view
PF3D7_0418100	Q8I1Q6	protein SOC1, putative	4	iCn3D view
PF3D7_0418500	Q8I1Q2	trafficking protein particle complex subunit 3, putative	1	iCn3D view
PF3D7_0418600	Q8I1Q1	regulator of chromosome condensation, putative	56	iCn3D view
PF3D7_0418700	Q8I1Q0	RNA-binding protein NOB1, putative	6	iCn3D view
PF3D7_0419500	Q8I1P2	conserved Plasmodium membrane protein, unknown function	7	iCn3D view
PF3D7_0419600	Q76NN6	ran-specific GTPase-activating protein 1, putative	8	iCn3D view
PF3D7_0419800	Q8I1P0	60S ribosomal protein L7ae/L30e, putative	4	iCn3D view
PF3D7_0419900	B9ZSJ6	phosphatidylinositol 4-kinase, putative	42	iCn3D view
PF3D7_0420300	Q8I1N6	AP2 domain transcription factor, putative	49	iCn3D view
PF3D7_0420600	A0A146LZZ4	apicomplexan kinetochore protein 6, putative	14	iCn3D view
PF3D7_0420700	Q8IFQ6	erythrocyte membrane protein 1, PfEMP1	2	iCn3D view
PF3D7_0420900	Q8IFQ5	erythrocyte membrane protein 1, PfEMP1	2	iCn3D view
PF3D7_0421300	Q8IFQ2	erythrocyte membrane protein 1, PfEMP1	2	iCn3D view
PF3D7_0422200	Q8IFP4	erythrocyte membrane-associated antigen	18	iCn3D view
PF3D7_0422300	Q8IFP3	alpha tubulin 2	8	iCn3D view

PF3D7_0422400	Q8IFP2	40S ribosomal protein S19	2	iCn3D view
PF3D7_0422500	Q8IFP1	pre-mRNA-splicing helicase BRR2, putative	10	iCn3D view
PF3D7_0422700	Q8IFN9	eukaryotic initiation factor 4A-III, putative	5	iCn3D view
PF3D7_0423000	Q8IFN6	conserved Plasmodium protein, unknown function	4	iCn3D view
PF3D7_0423500	Q8IFN1	glideosome associated protein with multiple membrane spans 2	8	iCn3D view
PF3D7_0423600	Q8IFN0	conserved Plasmodium protein, unknown function	12	iCn3D view
PF3D7_0423700	Q8IFM9	early transcribed membrane protein 4	5	iCn3D view
PF3D7_0424100	Q8IFM5	reticulocyte binding protein homologue 5	1	iCn3D view
PF3D7_0424200	C0H496	reticulocyte binding protein homologue 4	2	iCn3D view
PF3D7_0424400	Q8IFM2	surface-associated interspersed protein 4.2 (SURFIN 4.2)	1	iCn3D view
PF3D7_0424500	Q8IFM1	serine/threonine protein kinase, FIKK family	8	iCn3D view
PF3D7_0424600	Q8IFM0	Plasmodium exported protein (PHISTb)	8	iCn3D view
PF3D7_0424800	Q8IFL8	Plasmodium exported protein (PHISTb), unknown function	1	iCn3D view
PF3D7_0425100	Q8IFL4	Plasmodium exported protein (hyp6), unknown function	1	iCn3D view
PF3D7_0501000	Q8I490	Plasmodium exported protein, unknown function	3	iCn3D view
PF3D7_0501100	A0A143ZY16	heat shock protein 40, type II	1	iCn3D view
PF3D7_0501200	Q8I488	parasite-infected erythrocyte surface protein	12	iCn3D view
PF3D7_0501300	Q8I487	skeleton-binding protein 1	8	iCn3D view
PF3D7_0501500	Q8I485	rhoptry-associated protein 3	2	iCn3D view
PF3D7_0501600	Q8I484	rhoptry-associated protein 2	3	iCn3D view
PF3D7_0502000	Q8I480	vacuolar protein sorting-associated protein 11, putative	2	iCn3D view
PF3D7_0502100	Q8I479	HCNGP-like protein	4	iCn3D view
PF3D7_0503200	Q8I469	cell division control protein 6, putative	10	iCn3D view
PF3D7_0503400	Q8I467	actin-depolymerizing factor 1	1	iCn3D view
PF3D7_0503500	Q8I466	protein kinase, putative	4	iCn3D view

PF3D7_0503600	Q8I465	myosin B	1	iCn3D view
PF3D7_0503800	Q8I463	60S ribosomal protein L31	2	iCn3D view
PF3D7_0504100	Q8I460	AN1-type zinc finger protein, putative	3	iCn3D view
PF3D7_0504800	C0H4C4	conserved Plasmodium protein, unknown function	100	iCn3D view
PF3D7_0505000	C0H4C6	MMS19-like protein, putative	13	iCn3D view
PF3D7_0505100	Q8I451	trafficking protein particle complex subunit 8, putative	10	iCn3D view
PF3D7_0505200	Q8I450	actin-like protein, putative	1	iCn3D view
PF3D7_0505300	Q8I449	UDP-N-acetylglucosamine transporter, putative	1	iCn3D view
PF3D7_0505500	Q8I447	DNA mismatch repair protein MSH6, putative	16	iCn3D view
PF3D7_0505600	Q8I446	BCNT domain-containing protein, putative	4	iCn3D view
PF3D7_0505800	Q8I444	small ubiquitin-related modifier	4	iCn3D view
PF3D7_0505900	C0H4C7	mediator of RNA polymerase II transcription subunit 11, putative	3	iCn3D view
PF3D7_0506100	Q8I441	60S ribosomal subunit protein L24, putative	2	iCn3D view
PF3D7_0506500	C0H4C9	conserved Plasmodium protein, unknown function	4	iCn3D view
PF3D7_0506700	Q8I435	GTPase-activating protein, putative	3	iCn3D view
PF3D7_0506800	Q8I434	transcription factor 25, putative	4	iCn3D view
PF3D7_0506900	Q8I433	rhomboid protease ROM4	36	iCn3D view
PF3D7_0507100	Q8I431	60S ribosomal protein L4	12	iCn3D view
PF3D7_0507500	Q8I0V0	subtilisin-like protease 1	1	iCn3D view
PF3D7_0507600	Q8I427	protein CAF40, putative	9	iCn3D view
PF3D7_0507700	Q8I426	nuclear protein localization protein 4, putative	4	iCn3D view
PF3D7_0508000	Q8I423	6-cysteine protein P38	4	iCn3D view
PF3D7_0508100	Q8I422	SET domain protein, putative	8	iCn3D view
PF3D7_0508300	Q8I420	triose phosphate transporter	2	iCn3D view
PF3D7_0508400	Q8I419	CFA20 domain-containing protein, putative	1	iCn3D view

PF3D7_0508600	A0A5K1K8H8	conserved protein, unknown function	7	iCn3D view
PF3D7_0508900	Q8I414	protein AAP6	62	iCn3D view
PF3D7_0509000	Q8I0X0	alpha-soluble NSF attachment protein, putative	3	iCn3D view
PF3D7_0509100	Q8I413	structural maintenance of chromosomes protein 4, putative	10	iCn3D view
PF3D7_0509300	A0A5K1K9E3	conserved Plasmodium protein, unknown function	1	iCn3D view
PF3D7_0509400	Q8I410	RNA polymerase I	14	iCn3D view
PF3D7_0509500	Q8I409	ERCC4 domain-containing protein, putative	4	iCn3D view
PF3D7_0509800	Q8I406	phosphatidylinositol 4-kinase beta	18	iCn3D view
PF3D7_0509900	Q8I405	NSE4 domain-containing protein, putative	2	iCn3D view
PF3D7_0510200	Q8I402	peptidyl-prolyl cis-trans isomerase	5	iCn3D view
PF3D7_0510400	Q8I400	PDCD2 domain-containing protein, putative	3	iCn3D view
PF3D7_0511000	Q8I3Z5	translationally-controlled tumor protein homolog	3	iCn3D view
PF3D7_0511300	Q8I3Z3	MORN repeat protein, putative	3	iCn3D view
PF3D7_0511600	Q8I3Z0	apical rhoptry neck protein	1	iCn3D view
PF3D7_0511800	Q8I3Y8	inositol-3-phosphate synthase	5	iCn3D view
PF3D7_0512000	Q8I3Y6	prefoldin subunit 6	2	iCn3D view
PF3D7_0512200	Q8I3Y4	glutathione synthetase	2	iCn3D view
PF3D7_0512300	Q8I3Y3	CDK-activating kinase assembly factor MAT1	3	iCn3D view
PF3D7_0512600	A0A5K1K8H7	ras-related protein Rab-1B	6	iCn3D view
PF3D7_0513100	C0H4D5	conserved protein, unknown function	2	iCn3D view
PF3D7_0513200	Q8I3X5	CPSF domain-containing protein, putative	6	iCn3D view
PF3D7_0513300	Q8I3X4	purine nucleoside phosphorylase	5	iCn3D view
PF3D7_0513800	Q8I3W9	ras-related protein Rab-1A	4	iCn3D view
PF3D7_0514100	Q8I3W6	ATP-dependent DNA helicase UvrD	3	iCn3D view
PF3D7_0514900	Q8I3V9	conserved Plasmodium protein, unknown function	6	iCn3D view

PF3D7_0515000	Q8I3V8	pre-mRNA-splicing factor CWC2, putative	8	iCn3D view
PF3D7_0515400	C0H4E0	conserved protein, unknown function	7	iCn3D view
PF3D7_0515600	Q8I3V2	gamete egress protein GEP, putative	21	iCn3D view
PF3D7_0515700	Q8I3V1	glideosome-associated protein 40, putative	22	iCn3D view
PF3D7_0515900	Q8I3U9	NLI interacting factor-like phosphatase, putative	3	iCn3D view
PF3D7_0516100	Q8I3U7	cation-transporting ATPase 1	2	iCn3D view
PF3D7_0516200	A0A5K1K925	40S ribosomal protein S11	5	iCn3D view
PF3D7_0516300	Q8I3U5	tRNA pseudouridine synthase, putative	1	iCn3D view
PF3D7_0516400	Q8I3U4	phosducin-like protein 1, putative	11	iCn3D view
PF3D7_0516600	Q8I3U2	sporozoite surface antigen MB2	1	iCn3D view
PF3D7_0516800	Q8I3U0	AP2 domain transcription factor AP2-O2, putative	21	iCn3D view
PF3D7_0516900	Q8I3T9	60S ribosomal protein L2	7	iCn3D view
PF3D7_0517000	Q8I3T8	60S ribosomal protein L12, putative	3	iCn3D view
PF3D7_0517300	Q8I3T5	serine/arginine-rich splicing factor 1	21	iCn3D view
PF3D7_0517400	Q8I3T4	FACT complex subunit SPT16, putative	19	iCn3D view
PF3D7_0517700	Q8I3T1	eukaryotic translation initiation factor 3 subunit B, putative	10	iCn3D view
PF3D7_0517900	C0H4E5	zinc finger protein, putative	2	iCn3D view
PF3D7_0518000	C0H4E6	NOSIP domain-containing protein, putative	2	iCn3D view
PF3D7_0518200	C0H4E7	SWIB/MDM2 domain-containing protein	1	iCn3D view
PF3D7_0518500	A0A5K1K868	ATP-dependent RNA helicase DDX23, putative	15	iCn3D view
PF3D7_0518600	Q8I3S4	WD repeat-containing protein 26, putative	3	iCn3D view
PF3D7_0518700	Q8I3S3	mRNA-binding protein PUF1	2	iCn3D view
PF3D7_0519400	Q8I3R6	40S ribosomal protein S24	5	iCn3D view
PF3D7_0519500	Q8I3R5	CCR4 domain-containing protein 1, putative	7	iCn3D view
PF3D7_0519700	C0H4F1	FoP domain-containing protein, putative	11	iCn3D view

PF3D7_0519800	Q8I3R2	EELM2 domain-containing protein, putative	8	iCn3D view
PF3D7_0520000	Q8I3R0	40S ribosomal protein S9, putative	6	iCn3D view
PF3D7_0520100	C0H4F2	protein phosphatase PPM9, putative	4	iCn3D view
PF3D7_0520200	Q8I3Q8	mediator of RNA polymerase II transcription subunit 17, putative	8	iCn3D view
PF3D7_0520600	C0H4F3	bis(5'-nucleosyl)-tetraphosphatase [asymmetrical]	1	iCn3D view
PF3D7_0520700	Q8I3Q4	CDC73 domain-containing protein, putative	5	iCn3D view
PF3D7_0520900	P50250	adenosylhomocysteinase	7	iCn3D view
PF3D7_0521000	Q8I3Q2	conserved Plasmodium protein, unknown function	26	iCn3D view
PF3D7_0521200	C0H4F4	conserved protein, unknown function	3	iCn3D view
PF3D7_0521300	Q8I3P9	zinc finger protein, putative	1	iCn3D view
PF3D7_0521700	Q8I3P6	ATP-dependent RNA helicase DDX1, putative	4	iCn3D view
PF3D7_0521900	Q8I3P4	conserved Plasmodium protein, unknown function	7	iCn3D view
PF3D7_0522000	Q8I3P3	conserved Plasmodium protein, unknown function	1	iCn3D view
PF3D7_0522100	Q8I3P2	conserved Plasmodium protein, unknown function	15	iCn3D view
PF3D7_0522200	Q8I3P1	transcription initiation factor TFIID subunit 10, putative	1	iCn3D view
PF3D7_0522600	Q8I3N7	magnesium transporter NIPA, putative	10	iCn3D view
PF3D7_0522800	Q8I3N5	pre-mRNA-splicing factor BUD31, putative	1	iCn3D view
PF3D7_0522900	Q8I3N4	zinc finger protein, putative	12	iCn3D view
PF3D7_0523000	Q7K6A5	multidrug resistance protein 1	24	iCn3D view
PF3D7_0523400	Q8I3N0	DnaJ protein, putative	8	iCn3D view
PF3D7_0523800	Q8I3M7	food vacuole resident transporter 1	2	iCn3D view
PF3D7_0523900	C0H4G1	MerC domain-containing protein, putative	7	iCn3D view
PF3D7_0524000	Q8I3M5	karyopherin beta	12	iCn3D view
PF3D7_0524200	Q8I3M3	conserved Plasmodium membrane protein, unknown function	1	iCn3D view
PF3D7_0524400	A0A5K1K870	ribosome-interacting GTPase 1, putative	1	iCn3D view

PF3D7_0524500	Q8I3M0	conserved Plasmodium protein, unknown function	2	iCn3D view
PF3D7_0524800	Q8I3L7	ubiquitin fusion degradation protein 1, putative	1	iCn3D view
PF3D7_0525000	Q8I3L5	zinc finger protein, putative	3	iCn3D view
PF3D7_0525100	Q8I3L4	acyl-CoA synthetase	9	iCn3D view
PF3D7_0525200	Q8I3L3	structural maintenance of chromosomes protein 6, putative	4	iCn3D view
PF3D7_0525300	Q8I3L2	conserved protein, unknown function	1	iCn3D view
PF3D7_0525500	A0A5K1K8K0	WD repeat-containing protein, putative	1	iCn3D view
PF3D7_0525800	Q8I3K7	inner membrane complex protein 1g, putative	17	iCn3D view
PF3D7_0526200	C0H4G3	ADP-ribosylation factor GTPase-activating protein 2	17	iCn3D view
PF3D7_0526400	Q8I3K1	conserved Plasmodium protein, unknown function	1	iCn3D view
PF3D7_0526500	Q8I3K0	Suf domain-containing protein, putative	11	iCn3D view
PF3D7_0526600	Q8I3J9	conserved Plasmodium protein, unknown function	1	iCn3D view
PF3D7_0526800	Q8I3J7	conserved Plasmodium protein, unknown function	13	iCn3D view
PF3D7_0527000	Q8I3J5	DNA replication licensing factor MCM3, putative	15	iCn3D view
PF3D7_0527400	Q8I3J1	conserved Plasmodium protein, unknown function	2	iCn3D view
PF3D7_0527500	Q8I3J0	Hsc70-interacting protein	7	iCn3D view
PF3D7_0527900	A0A5K1K8X8	ATP-dependent RNA helicase DDX41, putative	6	iCn3D view
PF3D7_0528100	Q8I3I6	AP-1/2 complex subunit beta, putative	9	iCn3D view
PF3D7_0528200	Q8I3I5	eukaryotic translation initiation factor 3 subunit E, putative	4	iCn3D view
PF3D7_0528400	Q8I3I3	palmitoyltransferase DHHC7	16	iCn3D view
PF3D7_0528500	Q8I3I2	F-actin-capping protein subunit alpha, putative	1	iCn3D view
PF3D7_0528600	C0H4G5	conserved Plasmodium protein, unknown function	1	iCn3D view
PF3D7_0528700	Q8I3I0	peptidyl-prolyl cis-trans isomerase	1	iCn3D view
PF3D7_0528800	Q8I3H9	nuclear GTP-binding protein, putative	1	iCn3D view
PF3D7_0529000	Q8I3H7	T-cell immunomodulatory protein homolog, putative	1	iCn3D view

PF3D7_0529400	A0A143ZXE1	apicomplexan kinetochore protein 5, putative	50	iCn3D view
PF3D7_0529500	Q8I3H2	cell cycle regulator protein, putative	4	iCn3D view
PF3D7_0530000	Q8I3G7	conserved Plasmodium protein, unknown function	3	iCn3D view
PF3D7_0530100	Q8I3G6	SNARE protein, putative	2	iCn3D view
PF3D7_0530600	Q8I3G2	XAP-5 DNA binding protein, putative	9	iCn3D view
PF3D7_0530700	Q8I3G1	conserved Plasmodium protein, unknown function	1	iCn3D view
PF3D7_0530900	Q8I3F9	formin 1	14	iCn3D view
PF3D7_0531300	Q8I3F5	conserved Plasmodium protein, unknown function	1	iCn3D view
PF3D7_0532100	A0A5K1K7X4	early transcribed membrane protein 5	2	iCn3D view
PF3D7_0532400	Q8I3F0	lysine-rich membrane-associated PHISTb protein	3	iCn3D view
PF3D7_0532500	Q8I3E9	Plasmodium exported protein, unknown function	1	iCn3D view
PF3D7_0601500	C6KSL7	Plasmodium exported protein (PHISTb), unknown function	1	iCn3D view
PF3D7_0601600	C6KSL8	tetratricopeptide repeat protein, putative	4	iCn3D view
PF3D7_0601900	C6KSL9	conserved Plasmodium protein, unknown function	9	iCn3D view
PF3D7_0602000	C6KSM0	zinc finger protein, putative	9	iCn3D view
PF3D7_0602100	C6KSM1	ATP-dependent RNA helicase MTR4	4	iCn3D view
PF3D7_0602200	C6KSM2	MYND-type zinc finger protein, putative	2	iCn3D view
PF3D7_0602600	C6KSM6	SAC3 domain-containing protein, putative	9	iCn3D view
PF3D7_0602900	C6KSM9	conserved Plasmodium protein, unknown function	2	iCn3D view
PF3D7_0603000	C6KSN0	SDE2 domain-containing protein, putative	3	iCn3D view
PF3D7_0603100	C6KSN1	RNA-binding protein, putative	4	iCn3D view
PF3D7_0603400	Q6LFN2	trophozoite exported protein 1	26	iCn3D view
PF3D7_0603500	C6KSN3	cation/H+ antiporter	4	iCn3D view
PF3D7_0603600	C6KSN4	male development protein MD4, putative	4	iCn3D view
PF3D7_0603900	C6KSN7	conserved Plasmodium protein, unknown function	2	iCn3D view

PF3D7_0604000	C6KSN8	conserved Plasmodium protein, unknown function	5	iCn3D view
PF3D7_0604100	C6KSN9	AP2 domain transcription factor	17	iCn3D view
PF3D7_0604300	C6KSP1	conserved Plasmodium protein, unknown function	7	iCn3D view
PF3D7_0604500	C6KSP3	GYF domain-containing protein, putative	76	iCn3D view
PF3D7_0605100	C6KSP9	KH domain-containing protein, putative	9	iCn3D view
PF3D7_0605300	C6KSQ1	serine/threonine protein kinase ARK1	3	iCn3D view
PF3D7_0605500	C6KSQ3	cyclin dependent kinase binding protein, putative	7	iCn3D view
PF3D7_0605600	C6KSQ4	nucleoside diphosphate kinase, putative	4	iCn3D view
PF3D7_0605900	C6KSQ7	elongation of fatty acids protein, putative	2	iCn3D view
PF3D7_0606100	C6KSQ9	RNA-binding protein, putative	11	iCn3D view
PF3D7_0606200	C6KSR0	ubiquitin-conjugating enzyme E2 PEX4, putative	1	iCn3D view
PF3D7_0606500	C6KSR3	polypyrimidine tract-binding protein, putative	7	iCn3D view
PF3D7_0606700	C6KSR5	coatomer alpha subunit, putative	17	iCn3D view
PF3D7_0606800	C6KSR6	VFT protein	1	iCn3D view
PF3D7_0606900	C6KSR7	glutaredoxin-like protein	1	iCn3D view
PF3D7_0607000	C6KSR8	translation initiation factor IF-2, putative	9	iCn3D view
PF3D7_0607200	C6KSS0	RING zinc finger protein, putative	5	iCn3D view
PF3D7_0607400	C6KSS2	G-protein associated signal transduction protein, putative	1	iCn3D view
PF3D7_0607600	C6KSS4	spindle assembly abnormal protein 6, putative	9	iCn3D view
PF3D7_0607700	C6KSS5	conserved Plasmodium protein, unknown function	15	iCn3D view
PF3D7_0608000	C6KSS8	diphthine methyltransferase, putative	1	iCn3D view
PF3D7_0608100	C6KSS9	conserved Plasmodium protein, unknown function	1	iCn3D view
PF3D7_0608500	C6KST3	proteasome subunit alpha type-2, putative	2	iCn3D view
PF3D7_0608600	C6KST4	conserved Plasmodium protein, unknown function	14	iCn3D view
PF3D7_0608700	C6KST5	T-complex protein 1 subunit zeta	3	iCn3D view

PF3D7_0608800	Q6LFH8	ornithine aminotransferase	6	iCn3D view
PF3D7_0608900	C6KST6	conserved Plasmodium protein, unknown function	2	iCn3D view
PF3D7_0609000	C6KST7	nucleoporin NUP637, putative	24	iCn3D view
PF3D7_0609100	C6KST8	zinc transporter ZIP1, putative	2	iCn3D view
PF3D7_0609300	C6KSU0	conserved Plasmodium protein, unknown function	5	iCn3D view
PF3D7_0609700	C6KSU4	protein KIC6	40	iCn3D view
PF3D7_0609800	C6KSU5	palmitoyltransferase DHHC2, putative	4	iCn3D view
PF3D7_0609900	C6KSU6	tetratricopeptide repeat protein, putative	5	iCn3D view
PF3D7_0610400	C6KSV0	histone H3	5	iCn3D view
PF3D7_0610800	C6KSV3	transketolase	2	iCn3D view
PF3D7_0610900	C6KSV4	transcription elongation factor SPT5, putative	27	iCn3D view
PF3D7_0611400	C6KSV9	SWI/SNF-related matrix-associated actin-dependent regulator of chromatin	16	iCn3D view
PF3D7_0611600	C6KSW1	basal complex transmembrane protein 1	35	iCn3D view
PF3D7_0612000	C6KSW4	conserved Plasmodium protein, unknown function	2	iCn3D view
PF3D7_0612100	C6KSW5	eukaryotic translation initiation factor 3 subunit L, putative	1	iCn3D view
PF3D7_0612200	C6KSW6	leucine-rich repeat protein	25	iCn3D view
PF3D7_0612600	C6KSW9	cytoplasmic tRNA 2-thiolation protein 1, putative	1	iCn3D view
PF3D7_0612900	C6KSX2	nucleolar GTP-binding protein 1, putative	2	iCn3D view
PF3D7_0613100	C6KSX4	conserved Plasmodium protein, unknown function	1	iCn3D view
PF3D7_0613500	C6KSX7	AP-3 complex subunit beta, putative	11	iCn3D view
PF3D7_0613600	C6KSX8	conserved Plasmodium protein, unknown function	5	iCn3D view
PF3D7_0613700	C6KSX9	syntaxin-binding protein, putative	6	iCn3D view
PF3D7_0614100	C0H4H5	filamin domain-containing protein, putative	1	iCn3D view
PF3D7_0614200	C6KSY3	cytosolic Fe-S cluster assembly factor NAR1, putative	1	iCn3D view
PF3D7_0614300	C6KSY4	major facilitator superfamily-related transporter, putative	45	iCn3D view

PF3D7_0614400	C6KSY5	pre-mRNA-splicing factor CWF7, putative	1	iCn3D view
PF3D7_0614500	C6KSY6	60S ribosomal protein L19	6	iCn3D view
PF3D7_0614700	C6KSY8	F-box protein FBXO6, putative	3	iCn3D view
PF3D7_0614800	C6KSY9	endonuclease III-like protein 1, putative	1	iCn3D view
PF3D7_0614900	C6KSZ0	conserved Plasmodium membrane protein, unknown function	3	iCn3D view
PF3D7_0615000	C6KSZ1	conserved Plasmodium protein, unknown function	4	iCn3D view
PF3D7_0615500	C6KSZ6	cdc2-related protein kinase 5	3	iCn3D view
PF3D7_0615800	C6KSZ9	conserved oligomeric Golgi complex subunit 4, putative	5	iCn3D view
PF3D7_0616000	C6KT01	pyridoxal kinase	1	iCn3D view
PF3D7_0616100	C6KT02	conserved Plasmodium protein, unknown function	1	iCn3D view
PF3D7_0616200	C6KT03	kinetochore protein NDC80	12	iCn3D view
PF3D7_0616300	C6KT04	conserved Plasmodium protein, unknown function	1	iCn3D view
PF3D7_0616600	C6KT07	conserved protein, unknown function	5	iCn3D view
PF3D7_0617100	C6KT12	AP-2 complex subunit alpha, putative	10	iCn3D view
PF3D7_0617200	C6KT13	BFR1 domain-containing protein, putative	16	iCn3D view
PF3D7_0617300	C6KT14	RNA-binding protein, putative	2	iCn3D view
PF3D7_0617400	C6KT15	erythrocyte membrane protein 1, PfEMP1	1	iCn3D view
PF3D7_0617800	C6KT18	histone H2A	3	iCn3D view
PF3D7_0617900	C6KT19	histone H3 variant	6	iCn3D view
PF3D7_0618000	C6KT20	conserved Plasmodium membrane protein, unknown function	38	iCn3D view
PF3D7_0618200	C6KT22	conserved protein, unknown function	4	iCn3D view
PF3D7_0618300	C6KT23	60S ribosomal protein L27a, putative	4	iCn3D view
PF3D7_0618500	C6KT25	malate dehydrogenase	1	iCn3D view
PF3D7_0618600	C6KT26	rhomboid protease ROM10	1	iCn3D view
PF3D7_0618700	C6KT27	trafficking protein particle complex subunit 6A, putative	3	iCn3D view

PF3D7_0618900	A0A143ZZN8	phosphatidylinositol N-acetylglucosaminyltransferase subunit GPI1	5	iCn3D view
PF3D7_0619000	C6KT30	FPL domain-containing protein, putative	20	iCn3D view
PF3D7_0619200	C6KT32	conserved Plasmodium protein, unknown function	4	iCn3D view
PF3D7_0619400	C6KT34	cell division cycle protein 48 homologue, putative	19	iCn3D view
PF3D7_0619500	C6KT35	acyl-CoA synthetase	1	iCn3D view
PF3D7_0619700	C6KT37	F-box protein FBXO1, putative	5	iCn3D view
PF3D7_0619800	C6KT38	WD repeat-containing protein, putative	1	iCn3D view
PF3D7_0619900	C6KT39	splicing factor 3A subunit 2, putative	3	iCn3D view
PF3D7_0620300	C6KT43	conserved Plasmodium protein, unknown function	1	iCn3D view
PF3D7_0620500	C6KT45	cleavage stimulation factor subunit 1, putative	1	iCn3D view
PF3D7_0620700	C6KT47	DnaJ protein, putative	1	iCn3D view
PF3D7_0621200	C6KT50	pyridoxine biosynthesis protein PDX1	4	iCn3D view
PF3D7_0621400	C6KT52	Pf77 protein	1	iCn3D view
PF3D7_0621800	C6KT55	nascent polypeptide-associated complex subunit alpha, putative	5	iCn3D view
PF3D7_0621900	C6KT56	signal recognition particle subunit SRP68, putative	8	iCn3D view
PF3D7_0622100	C6KT58	conserved Plasmodium protein, unknown function	3	iCn3D view
PF3D7_0622200	C6KT59	tRNA-2-methylthio-N(6)-dimethylallyladenosine synthase	3	iCn3D view
PF3D7_0622300	C6KT60	vacuolar transporter chaperone, putative	4	iCn3D view
PF3D7_0622500	C6KT62	RNA methyltransferase, putative	1	iCn3D view
PF3D7_0622800	C6KT64	leucine--tRNA ligase, putative	7	iCn3D view
PF3D7_0622900	C6KT65	AP2 domain transcription factor AP2Tel	13	iCn3D view
PF3D7_0623000	C6KT66	chorismate synthase	2	iCn3D view
PF3D7_0623100	C6KT67	nuclear polyadenylated RNA-binding protein NAB2, putative	29	iCn3D view
PF3D7_0623600	C6KT72	splicing factor-like protein 1, putative	2	iCn3D view
PF3D7_0623800	C6KT74	tyrosine kinase-like protein, putative	24	iCn3D view

PF3D7_0623900	C6KT75	ribonuclease H2 subunit A, putative	1	iCn3D view
PF3D7_0624000	C6KT76	hexokinase	4	iCn3D view
PF3D7_0624200	C6KT78	SURP domain-containing protein, putative	4	iCn3D view
PF3D7_0624800	C6KT84	conserved Plasmodium protein, unknown function	1	iCn3D view
PF3D7_0624900	C6KT85	GAS8-like protein, putative	1	iCn3D view
PF3D7_0625000	C6KT86	sphingomyelin synthase 1, putative	2	iCn3D view
PF3D7_0625200	C6KT88	conserved Plasmodium protein, unknown function	9	iCn3D view
PF3D7_0625600	C6KT92	poly(A) polymerase PAP, putative	1	iCn3D view
PF3D7_0625800	C6KT94	conserved Plasmodium protein, unknown function	3	iCn3D view
PF3D7_0626000	C6KT96	conserved Plasmodium protein, unknown function	1	iCn3D view
PF3D7_0626400	C6KTA0	CRAL/TRIO domain-containing protein, putative	21	iCn3D view
PF3D7_0626500	C6KTA1	centrosomal protein CEP135, putative	2	iCn3D view
PF3D7_0626700	C6KTA3	ATPase	1	iCn3D view
PF3D7_0626800	C6KTA4	pyruvate kinase	9	iCn3D view
PF3D7_0627100	C6KTA7	ankyrin-repeat protein, putative	21	iCn3D view
PF3D7_0627300	C6KTA9	E3 ubiquitin-protein ligase RNF5, putative	4	iCn3D view
PF3D7_0627500	C6KTB1	protein DJ-1	4	iCn3D view
PF3D7_0627600	C6KTB2	conserved Plasmodium protein, unknown function	1	iCn3D view
PF3D7_0627700	C6KTB3	transportin	9	iCn3D view
PF3D7_0627800	C6KTB4	acetyl-CoA synthetase	16	iCn3D view
PF3D7_0628100	C6KTB7	HECT domain-containing protein 1, putative	140	iCn3D view
PF3D7_0628200	C6KTB8	eukaryotic translation initiation factor 2-alpha kinase	31	iCn3D view
PF3D7_0628300	C6KTB9	choline/ethanolaminephosphotransferase, putative	3	iCn3D view
PF3D7_0628600	C6KTC1	DNA methyltransferase 1-associated protein 1, putative	1	iCn3D view
PF3D7_0629100	C6KTC6	nicotinate phosphoribosyltransferase, putative	5	iCn3D view

PF3D7_0629200	C6KTC7	DnaJ protein, putative	1	iCn3D view
PF3D7_0629400	C6KTC9	polyadenylate-binding protein 3, putative	3	iCn3D view
PF3D7_0629500	C6KTD0	amino acid transporter AAT1	8	iCn3D view
PF3D7_0629800	C6KTD3	cullin-2, putative	4	iCn3D view
PF3D7_0629900	C6KTD4	CRAL/TRIO domain-containing protein, putative	1	iCn3D view
PF3D7_0630100	C6KTD6	alpha/beta hydrolase, putative	2	iCn3D view
PF3D7_0630300	C6KTD8	DNA polymerase epsilon catalytic subunit A, putative	11	iCn3D view
PF3D7_0630500	C6KTE0	ribosome biogenesis protein YTM1, putative	1	iCn3D view
PF3D7_0630600	C6KTE1	deubiquitinating enzyme MINDY, putative	10	iCn3D view
PF3D7_0630800	C6KTE3	TPH domain-containing protein, putative	3	iCn3D view
PF3D7_0630900	C6KTE4	ATP-dependent RNA helicase HAS1	3	iCn3D view
PF3D7_0631000	C6KTE5	tetratricopeptide repeat protein, putative	4	iCn3D view
PF3D7_0631100	C6KTE6	Plasmodium exported protein (PHISTb), unknown function	1	iCn3D view
PF3D7_0632500	C6KTF7	erythrocyte membrane protein 1, PfEMP1	1	iCn3D view
PF3D7_0632800	C6KTF9	erythrocyte membrane protein 1, PfEMP1	1	iCn3D view
PF3D7_0701900	Q8IC48	Plasmodium exported protein, unknown function	1	iCn3D view
PF3D7_0702000	C0H4K0	Plasmodium exported protein (hyp12), unknown function	1	iCn3D view
PF3D7_0702300	Q8IC44	sporozoite threonine and asparagine-rich protein	2	iCn3D view
PF3D7_0702400	Q8IC43	small exported membrane protein 1	5	iCn3D view
PF3D7_0702500	Q8IC42	Plasmodium exported protein, unknown function	28	iCn3D view
PF3D7_0702600	Q8I6S7	chitinase, fragment	1	iCn3D view
PF3D7_0703500	Q8IC35	erythrocyte membrane-associated antigen	34	iCn3D view
PF3D7_0703800	Q8IC32	conserved Plasmodium protein, unknown function	2	iCn3D view
PF3D7_0704000	C0H4K3	conserved Plasmodium membrane protein, unknown function	6	iCn3D view
PF3D7_0704500	C0H4K5	serine/threonine protein kinase, putative	8	iCn3D view

PF3D7_0704700	Q8IC23	phosphopantetheine adenylyltransferase, putative	6	iCn3D view
PF3D7_0704900	Q8IC21	peptide chain release factor 2	5	iCn3D view
PF3D7_0705000	Q8IC20	mRNA cap guanine-N7 methyltransferase, putative	4	iCn3D view
PF3D7_0705300	Q8IC17	origin recognition complex subunit 2	7	iCn3D view
PF3D7_0705400	Q8IC16	DNA replication licensing factor MCM7	6	iCn3D view
PF3D7_0706000	C0H4L1	importin-7, putative	15	iCn3D view
PF3D7_0706100	A0A143ZXJ2	EF hand domain-containing protein, putative	1	iCn3D view
PF3D7_0706300	C0H4L4	apicomplexan kinetochore protein 11, putative	2	iCn3D view
PF3D7_0706400	C0H4L5	60S ribosomal protein L37	4	iCn3D view
PF3D7_0707400	C0H4M1	ATPase family AAA domain-containing protein 3A, putative	2	iCn3D view
PF3D7_0707500	Q8IC14	conserved Plasmodium protein, unknown function	5	iCn3D view
PF3D7_0707600	Q8IC13	mediator of RNA polymerase II transcription subunit 10, putative	7	iCn3D view
PF3D7_0707700	Q8IC12	E3 ubiquitin-protein ligase, putative	13	iCn3D view
PF3D7_0708000	C0H4M2	cytoskeleton associated protein, putative	3	iCn3D view
PF3D7_0708300	Q8IC06	EKC/KEOPS complex subunit BUD32	1	iCn3D view
PF3D7_0708400	Q8IC05	heat shock protein 90	43	iCn3D view
PF3D7_0708500	Q8IC04	heat shock protein 86 family protein	27	iCn3D view
PF3D7_0708700	Q8IC02	cytochrome c oxidase subunit 4, putative	2	iCn3D view
PF3D7_0708800	Q8IC01	heat shock protein 110	18	iCn3D view
PF3D7_0709000	Q8IBZ9	chloroquine resistance transporter	5	iCn3D view
PF3D7_0709300	Q8IBZ6	mediator of RNA polymerase II transcription subunit 14, putative	8	iCn3D view
PF3D7_0709400	Q8IBZ5	Cg7 protein	12	iCn3D view
PF3D7_0709500	Q8IBZ4	nucleic acid-binding protein, putative	2	iCn3D view
PF3D7_0709600	C0H4M3	ribonucleases P/MRP protein subunit POP1, putative	4	iCn3D view
PF3D7_0709900	Q8IBZ1	hydrolase, putative	5	iCn3D view

PF3D7_0710000	Q8IBZ0	conserved Plasmodium protein, unknown function	6	iCn3D view
PF3D7_0710400	A0A143ZWE7	DNA repair protein RAD14, putative	2	iCn3D view
PF3D7_0710600	Q8IBY4	60S ribosomal protein L34	2	iCn3D view
PF3D7_0710700	Q8IBY3	conserved Plasmodium protein, unknown function	1	iCn3D view
PF3D7_0710800	Q8IBY2	protein transport protein USE1, putative	3	iCn3D view
PF3D7_0711000	P46468	AAA family ATPase, CDC48 subfamily	1	iCn3D view
PF3D7_0711100	Q8IBX9	conserved protein, unknown function	1	iCn3D view
PF3D7_0711300	Q8IBX7	conserved Plasmodium protein, unknown function	1	iCn3D view
PF3D7_0711400	Q8IBX6	histone deacetylase complex subunit SAP18, putative	25	iCn3D view
PF3D7_0712000	Q8IBX2	erythrocyte membrane protein 1, PfEMP1	2	iCn3D view
PF3D7_0712300	Q8IBX1	erythrocyte membrane protein 1, PfEMP1	2	iCn3D view
PF3D7_0712800	Q8IBW8	erythrocyte membrane protein 1, PfEMP1	1	iCn3D view
PF3D7_0712900	Q8IBW7	erythrocyte membrane protein 1, PfEMP1	1	iCn3D view
PF3D7_0713100	Q8IBW5	Pfmc-2TM Maurer's cleft two transmembrane protein	1	iCn3D view
PF3D7_0713400	Q8IBW3	GPCR-like receptor SR25	3	iCn3D view
PF3D7_0713500	Q8IBW2	conserved Plasmodium protein, unknown function	1	iCn3D view
PF3D7_0713800	Q8IBV9	negative elongation factor A, putative	1	iCn3D view
PF3D7_0714000	Q8IBV7	histone H2B variant	6	iCn3D view
PF3D7_0714200	Q8IBV5	conserved Plasmodium protein, unknown function	39	iCn3D view
PF3D7_0714300	Q8IBV4	palmitoyltransferase DHHC4, putative	5	iCn3D view
PF3D7_0714500	Q8IBV2	transcription elongation factor s-II, putative	6	iCn3D view
PF3D7_0715200	Q8IBU8	conserved Plasmodium protein, unknown function	23	iCn3D view
PF3D7_0715900	Q8IBU1	cation diffusion facilitator family protein, putative	22	iCn3D view
PF3D7_0716000	Q8IBU0	RNA-binding protein, putative	23	iCn3D view
PF3D7_0716200	Q8IBT8	PDCD2 domain-containing protein, putative	2	iCn3D view

PF3D7_0716300	Q8IBT7	conserved protein, unknown function	3	iCn3D view
PF3D7_0716400	A0A143ZY73	transcription initiation factor IIA subunit 1, putative	2	iCn3D view
PF3D7_0716800	Q8IBT2	eukaryotic translation initiation factor 3 subunit I, putative	11	iCn3D view
PF3D7_0717100	C0H4N1	CRAL/TRIO domain-containing protein, putative	10	iCn3D view
PF3D7_0717300	C0H4N3	transcription initiation factor IIE subunit alpha, putative	2	iCn3D view
PF3D7_0717400	Q8IBS6	queuine tRNA-ribosyltransferase, putative	2	iCn3D view
PF3D7_0717500	Q8IBS5	calcium-dependent protein kinase 4	5	iCn3D view
PF3D7_0717600	A0A143ZVJ7	inner membrane complex protein, putative	7	iCn3D view
PF3D7_0717700	Q8IBS3	serine--tRNA ligase, putative	11	iCn3D view
PF3D7_0718000	C0H4N5	dynein heavy chain, putative	2	iCn3D view
PF3D7_0718100	Q8IBR9	exported serine/threonine protein kinase	9	iCn3D view
PF3D7_0718500	Q8IBR6	prefoldin subunit 3, putative	1	iCn3D view
PF3D7_0718600	A0A143ZXK1	conserved Plasmodium protein, unknown function	1	iCn3D view
PF3D7_0719000	Q8IBR1	translation machinery-associated protein, putative	4	iCn3D view
PF3D7_0719300	Q8IBQ9	actin-related protein ARP6	17	iCn3D view
PF3D7_0719500	A0A143ZVJ8	cell division control protein CDC50A	7	iCn3D view
PF3D7_0719600	Q8IBQ6	60S ribosomal protein L11a, putative	1	iCn3D view
PF3D7_0719700	Q8IBQ5	40S ribosomal protein S10, putative	3	iCn3D view
PF3D7_0720700	Q8IBP4	phosphoinositide-binding protein PX1	23	iCn3D view
PF3D7_0721100	Q8IBP0	conserved protein, unknown function	1	iCn3D view
PF3D7_0721200	Q8IBN9	conserved Plasmodium protein, unknown function	3	iCn3D view
PF3D7_0721400	Q8IBN7	rhoptry protein, putative	1	iCn3D view
PF3D7_0721600	Q8IBN5	40S ribosomal protein S5, putative	4	iCn3D view
PF3D7_0721800	Q8IBN3	conserved Plasmodium protein, unknown function	2	iCn3D view
PF3D7_0722000	Q8IBN2	conserved Plasmodium protein, unknown function	3	iCn3D view

PF3D7_0722200	Q8IBN1	rhoptry-associated leucine zipper-like protein 1	7	iCn3D view
PF3D7_0722400	Q8IBM9	Obg-like ATPase 1, putative	4	iCn3D view
PF3D7_0722500	Q8IBM8	pre-mRNA-splicing factor CWC15, putative	7	iCn3D view
PF3D7_0722600	Q8IBM7	U3 small nucleolar RNA-associated protein 7, putative	6	iCn3D view
PF3D7_0722900	Q8IBM4	conserved protein, unknown function	1	iCn3D view
PF3D7_0723300	Q8IBM0	conserved protein, unknown function	12	iCn3D view
PF3D7_0723900	Q8IBL4	RNA-binding protein, putative	22	iCn3D view
PF3D7_0724000	C0H4P1	Rab GTPase activator and protein kinase, putative	3	iCn3D view
PF3D7_0724100	Q8IBL1	conserved Plasmodium protein, unknown function	33	iCn3D view
PF3D7_0724200	Q8IBL0	type 2A phosphatase-associated protein 42, putative	1	iCn3D view
PF3D7_0724600	Q8IBK6	protein kinase, putative	12	iCn3D view
PF3D7_0724700	C0H4P2	bromodomain protein 6, putative	27	iCn3D view
PF3D7_0724800	Q8IBK3	kelch domain-containing protein, putative	3	iCn3D view
PF3D7_0724900	Q8IBK2	kinesin-20, putative	1	iCn3D view
PF3D7_0725200	Q8IBJ9	mago nashi protein homologue, putative	2	iCn3D view
PF3D7_0725300	Q8IBJ8	WD repeat-containing protein, putative	3	iCn3D view
PF3D7_0726100	Q8IBJ5	Plasmodium exported protein, unknown function	3	iCn3D view
PF3D7_0726400	C0H4P4	conserved Plasmodium membrane protein, unknown function	31	iCn3D view
PF3D7_0726500	Q8IBJ1	ubiquitin carboxyl-terminal hydrolase, putative	52	iCn3D view
PF3D7_0726600	Q8IBJ0	conserved Plasmodium protein, unknown function	2	iCn3D view
PF3D7_0727000	Q8IBI7	vacuolar protein sorting-associated protein 53, putative	1	iCn3D view
PF3D7_0727400	Q8IBI3	proteasome subunit alpha type-5, putative	8	iCn3D view
PF3D7_0727700	Q8IBI0	conserved Plasmodium protein, unknown function	1	iCn3D view
PF3D7_0727800	Q8IBH9	cation transporting ATPase, putative	8	iCn3D view
PF3D7_0727900	Q8IBH8	conserved Plasmodium protein, unknown function	8	iCn3D view

PF3D7_0728000	Q8IBH7	eukaryotic translation initiation factor 2 subunit alpha	3	iCn3D view
PF3D7_0728100	Q8IBH6	anaphase-promoting complex subunit 1, putative	10	iCn3D view
PF3D7_0728900	C0H4P9	RNA-binding protein, putative	6	iCn3D view
PF3D7_0729300	Q8IBG6	60S ribosomal export protein NMD3, putative	9	iCn3D view
PF3D7_0729400	Q8IBG5	ribosome biogenesis protein BRX1, putative	4	iCn3D view
PF3D7_0729500	Q8I6Z2	N6-adenosine-methyltransferase MT-A70	5	iCn3D view
PF3D7_0729700	Q8IBG3	zinc finger protein, putative	1	iCn3D view
PF3D7_0729900	Q8IBG1	dynein heavy chain, putative	2	iCn3D view
PF3D7_0730200	Q8IBF8	AP-4 complex subunit beta, putative	2	iCn3D view
PF3D7_0730300	Q8IBF6	AP2 domain transcription factor AP2-L, putative	7	iCn3D view
PF3D7_0730400	Q8IBF5	IMP1-like protein, putative	4	iCn3D view
PF3D7_0730800	Q8IBF3	Plasmodium exported protein, unknown function	5	iCn3D view
PF3D7_0731100	Q8IBF1	EMP1-trafficking protein	9	iCn3D view
PF3D7_0731200	Q8IBF0	Plasmodium exported protein, unknown function	1	iCn3D view
PF3D7_0731300	Q8IBE9	Plasmodium exported protein (PHISTb), unknown function	5	iCn3D view
PF3D7_0731500	Q8IBE8	erythrocyte binding antigen-175	14	iCn3D view
PF3D7_0731600	Q8I6Z1	acyl-CoA synthetase	5	iCn3D view
PF3D7_0731800	C0H4Q4	alpha/beta hydrolase, putative	2	iCn3D view
PF3D7_0800700	A0A143ZWG8	surface-associated interspersed protein 8.3 (SURFIN 8.3)	1	iCn3D view
PF3D7_0801300	Q8IAL0	von Willebrand factor A domain-related protein	1	iCn3D view
PF3D7_0801400	A0A5K1K9E5	conserved Plasmodium protein, unknown function	3	iCn3D view
PF3D7_0801500	Q8IAL3	nucleolar protein 10, putative	2	iCn3D view
PF3D7_0801800	Q8IAL6	mannose-6-phosphate isomerase, putative	25	iCn3D view
PF3D7_0802000	Q8IAM0	glutamate dehydrogenase, putative	14	iCn3D view
PF3D7_0802100	Q8IAM1	AP2 domain transcription factor AP2-LT	15	iCn3D view

PF3D7_0802200	Q8IAM2	1-cys peroxiredoxin	2	iCn3D view
PF3D7_0802400	Q8IAM4	cyclin-like protein, putative	5	iCn3D view
PF3D7_0802500	C0H4Q9	inositol 5-phosphatase, putative	4	iCn3D view
PF3D7_0802600	C0H4R1	adenylyl cyclase beta	28	iCn3D view
PF3D7_0802800	Q8IAM8	serine/threonine protein phosphatase 2B catalytic subunit A	4	iCn3D view
PF3D7_0803000	Q8IAN0	peptidyl-prolyl cis-trans isomerase	6	iCn3D view
PF3D7_0803100	Q8IAN1	U3 small nucleolar RNA-associated protein 14, putative	9	iCn3D view
PF3D7_0803200	Q8IAN2	filament assembling protein, putative	15	iCn3D view
PF3D7_0803400	Q8IAN4	DNA repair and recombination protein RAD54, putative	11	iCn3D view
PF3D7_0803500	Q8IAN5	AAA family ATPase, putative	11	iCn3D view
PF3D7_0803700	Q8IAN7	tubulin gamma chain	1	iCn3D view
PF3D7_0804000	Q8IAN8	cactin homolog, putative	7	iCn3D view
PF3D7_0804300	Q8IAN9	zinc finger protein, putative	1	iCn3D view
PF3D7_0804600	C0H4R3	tRNA pseudouridine synthase, putative	2	iCn3D view
PF3D7_0804900	Q8IAP4	GTPase-activating protein, putative	10	iCn3D view
PF3D7_0805500	C0H4R6	conserved Plasmodium protein, unknown function	5	iCn3D view
PF3D7_0805600	A0A143ZY93	phosphatidic acid phosphatase 2	2	iCn3D view
PF3D7_0805700	C0H4R8	serine/threonine protein kinase, FIKK family	38	iCn3D view
PF3D7_0805900	A0A143ZVL3	conserved protein, unknown function	1	iCn3D view
PF3D7_0806000	C0H4S0	AAA family ATPase, putative	2	iCn3D view
PF3D7_0806100	A0A143ZXM1	conserved Plasmodium protein, unknown function	7	iCn3D view
PF3D7_0806300	C0H4S2	ferlin-like protein, putative	9	iCn3D view
PF3D7_0806500	Q8IAQ5	DnaJ protein, putative	6	iCn3D view
PF3D7_0806600	C0H4S3	kinesin-like protein, putative	1	iCn3D view
PF3D7_0806700	A0A143ZVL6	conserved Plasmodium membrane protein, unknown function	14	iCn3D view

PF3D7_0806800	Q8IAQ8	V-type proton ATPase subunit a, putative	18	iCn3D view
PF3D7_0807100	Q8IAR1	DNA helicase PSH3	2	iCn3D view
PF3D7_0807200	C0H4S6	conserved Plasmodium membrane protein, unknown function	2	iCn3D view
PF3D7_0807300	Q7K6B0	ras-related protein Rab-18	2	iCn3D view
PF3D7_0807600	C0H4S8	conserved Plasmodium protein, unknown function	28	iCn3D view
PF3D7_0807700	Q8IAR5	serine protease DegP	1	iCn3D view
PF3D7_0807800	Q8IAR6	26S proteasome regulatory subunit RPN10, putative	2	iCn3D view
PF3D7_0807900	Q8IAR7	tyrosine--tRNA ligase	3	iCn3D view
PF3D7_0808100	C0H4T0	AP-3 complex subunit delta, putative	9	iCn3D view
PF3D7_0808300	Q8IAS1	ubiquitin regulatory protein, putative	7	iCn3D view
PF3D7_0808600	Q8IAS3	erythrocyte membrane protein 1, PfEMP1	2	iCn3D view
PF3D7_0808700	Q8IAS4	erythrocyte membrane protein 1, PfEMP1	1	iCn3D view
PF3D7_0809100	Q8IAS7	erythrocyte membrane protein 1, PfEMP1	1	iCn3D view
PF3D7_0809200	Q8IAS8	asparagine-rich antigen Pfa55-14	2	iCn3D view
PF3D7_0809600	C0H4T3	peptidase family C50, putative	12	iCn3D view
PF3D7_0809700	Q8IAT2	RuvB-like helicase 1	1	iCn3D view
PF3D7_0809800	C0H4T4	conserved Plasmodium protein, unknown function	1	iCn3D view
PF3D7_0809900	Q8IAT4	JmjC domain-containing protein 1, putative	6	iCn3D view
PF3D7_0810000	Q8IAT5	acyl-CoA binding protein, putative	2	iCn3D view
PF3D7_0810300	C0H4T6	protein phosphatase PPM5, putative	12	iCn3D view
PF3D7_0810500	Q8IAU0	protein phosphatase PPM7, putative	3	iCn3D view
PF3D7_0810800	Q8IAU3	hydroxymethyldihydropterin pyrophosphokinase-dihydropteroate synthase	5	iCn3D view
PF3D7_0810900	Q8IAU4	conserved Plasmodium protein, unknown function	2	iCn3D view
PF3D7_0811000	Q8IAU5	cullin-1, putative	1	iCn3D view
PF3D7_0811200	Q8IAU7	ER membrane protein complex subunit 1, putative	1	iCn3D view

PF3D7_0811300	C0H4T9	CCR4-associated factor 1	13	iCn3D view
PF3D7_0811400	C0H4U0	conserved protein, unknown function	7	iCn3D view
PF3D7_0811500	A0A5K1K956	histone-arginine methyltransferase CARM1, putative	7	iCn3D view
PF3D7_0812400	Q8IAW0	karyopherin alpha	9	iCn3D view
PF3D7_0812500	Q8IAW1	RNA-binding protein, putative	62	iCn3D view
PF3D7_0812600	Q8IAW2	ubiquitin-conjugating enzyme E2, putative	1	iCn3D view
PF3D7_0812700	Q8IAW3	U1 small nuclear ribonucleoprotein C, putative	10	iCn3D view
PF3D7_0812800	C0H4U3	zinc finger protein, putative	1	iCn3D view
PF3D7_0812900	Q8IAW4	conserved Plasmodium protein, unknown function	2	iCn3D view
PF3D7_0813100	Q8IAW6	conserved Plasmodium protein, unknown function	28	iCn3D view
PF3D7_0813200	Q8IAW7	CS domain protein, putative	1	iCn3D view
PF3D7_0813300	C0H4U4	NPL domain-containing protein, putative	12	iCn3D view
PF3D7_0813400	C0H4U5	conserved protein, unknown function	8	iCn3D view
PF3D7_0813600	Q8IAX2	translation initiation factor SUI1, putative	6	iCn3D view
PF3D7_0813900	Q8IAX5	40S ribosomal protein S16, putative	2	iCn3D view
PF3D7_0814000	Q8IAX6	60S ribosomal protein L13-2, putative	8	iCn3D view
PF3D7_0814200	Q8IAX8	DNA/RNA-binding protein Alba 1	13	iCn3D view
PF3D7_0814400	Q8IAY0	phospholipase PLA1, putative	1	iCn3D view
PF3D7_0814500	Q8IAY2	conserved protein, unknown function	5	iCn3D view
PF3D7_0815100	Q8IAY8	heptatricopeptide repeat and RAP domain-containing protein, putative	2	iCn3D view
PF3D7_0815200	Q8IAY9	importin subunit beta, putative	6	iCn3D view
PF3D7_0815500	Q8IAZ2	conserved Plasmodium protein, unknown function	2	iCn3D view
PF3D7_0815600	Q8IAZ3	eukaryotic translation initiation factor 3 subunit G, putative	7	iCn3D view
PF3D7_0816000	Q8IAZ7	ribosome assembly protein RRB1, putative	1	iCn3D view
PF3D7_0816200	Q8IAZ9	vacuolar protein sorting-associated protein 2, putative	1	iCn3D view

PF3D7_0816400	C0H4U9	EF-hand calcium-binding domain-containing protein, putative	1	iCn3D view
PF3D7_0816900	Q8IB06	adenylate kinase 2	2	iCn3D view
PF3D7_0817300	Q8IB09	conserved Plasmodium protein, unknown function	10	iCn3D view
PF3D7_0817600	Q8IB11	conserved protein, unknown function	9	iCn3D view
PF3D7_0817700	C0H4V4	rhoptry neck protein 5	1	iCn3D view
PF3D7_0817900	Q8IB14	high mobility group protein B2	3	iCn3D view
PF3D7_0818100	Q8IB16	C3H1-type zinc finger protein CZIF2	5	iCn3D view
PF3D7_0818200	C0H4V6	14-3-3 protein	15	iCn3D view
PF3D7_0818500	Q8IB20	zinc finger protein, putative	4	iCn3D view
PF3D7_0818700	C0H4V8	DNA helicase, putative	11	iCn3D view
PF3D7_0818900	Q8IB24	heat shock protein 70	24	iCn3D view
PF3D7_0819000	Q8IB25	ribonuclease, putative	8	iCn3D view
PF3D7_0819500	C0H4W0	conserved protein, unknown function	2	iCn3D view
PF3D7_0819600	Q8IB31	ubiquitin-like protein, putative	6	iCn3D view
PF3D7_0819900	C0H4W2	U6 snRNA-associated Sm-like protein LSm3, putative	1	iCn3D view
PF3D7_0820200	Q8IB37	phosphatidylglycerophosphate synthase	5	iCn3D view
PF3D7_0820300	Q8IB38	conserved Plasmodium protein, unknown function	1	iCn3D view
PF3D7_0820500	Q8IB40	protein transport protein YIF1, putative	9	iCn3D view
PF3D7_0820900	Q8IB43	conserved Plasmodium protein, unknown function	14	iCn3D view
PF3D7_0821000	Q8IB44	conserved Plasmodium protein, unknown function	6	iCn3D view
PF3D7_0821100	C0H4W5	protein kinase 1	6	iCn3D view
PF3D7_0821300	Q8IB47	ATP-dependent RNA helicase DHX36, putative	5	iCn3D view
PF3D7_0821400	Q8IB48	conserved Plasmodium protein, unknown function	12	iCn3D view
PF3D7_0821500	Q8IB49	ribosomal RNA small subunit methyltransferase NEP1, putative	1	iCn3D view
PF3D7_0821600	Q8IB50	polyribonucleotide 5'-hydroxyl-kinase Clp1, putative	1	iCn3D view

PF3D7_0821800	C0H4W6	protein transport protein SEC61 subunit beta, putative	6	iCn3D view
PF3D7_0822400	C0H4W9	conserved Plasmodium protein, unknown function	1	iCn3D view
PF3D7_0822600	Q8IB60	protein transport protein SEC23	4	iCn3D view
PF3D7_0822800	C0H4X1	U5 small nuclear ribonucleoprotein 40 kDa protein, putative	1	iCn3D view
PF3D7_0823000	Q8IB64	serine/threonine protein kinase VPS15, putative	2	iCn3D view
PF3D7_0823100	C0H4X2	RWD domain-containing protein, putative	2	iCn3D view
PF3D7_0823200	Q8IB66	RNA-binding protein, putative	11	iCn3D view
PF3D7_0823300	Q8IB67	histone acetyltransferase GCN5	21	iCn3D view
PF3D7_0823500	Q8IB69	inner membrane complex protein 1i, putative	2	iCn3D view
PF3D7_0823800	Q8IB72	DnaJ protein, putative	9	iCn3D view
PF3D7_0824200	C0H4X4	conserved Plasmodium protein, unknown function	2	iCn3D view
PF3D7_0824400	Q8IB78	nucleoside transporter 2	23	iCn3D view
PF3D7_0824600	C0H4X5	Fe-S cluster assembly protein DRE2, putative	1	iCn3D view
PF3D7_0824800	Q8IB82	conserved Plasmodium membrane protein, unknown function	19	iCn3D view
PF3D7_0824900	Q8IB83	conserved Plasmodium protein, unknown function	5	iCn3D view
PF3D7_0825000	C0H4X7	conserved Plasmodium protein, unknown function	65	iCn3D view
PF3D7_0825300	Q8IB87	SAYSvFN domain-containing protein, putative	1	iCn3D view
PF3D7_0825500	Q8IB88	protein KRI1, putative	11	iCn3D view
PF3D7_0825600	Q8IB89	cytochrome c oxidase assembly factor 5, putative	1	iCn3D view
PF3D7_0825900	Q8IB92	conserved Plasmodium protein, unknown function	16	iCn3D view
PF3D7_0826000	Q8IB93	conserved Plasmodium protein, unknown function	1	iCn3D view
PF3D7_0826300	Q8IB96	SPRY domain, putative	7	iCn3D view
PF3D7_0826500	C0H4Y0	ubiquitin conjugation factor E4 B, putative	9	iCn3D view
PF3D7_0826600	Q8IB99	SNARE protein, putative	1	iCn3D view
PF3D7_0826700	Q8IBA0	receptor for activated c kinase	12	iCn3D view

PF3D7_0826900	C0H4Y1	conserved Plasmodium protein, unknown function	31	iCn3D view
PF3D7_0827000	Q8IBA2	ATP-dependent RNA helicase DBP10, putative	6	iCn3D view
PF3D7_0827100	Q8IBA3	translation initiation factor IF-2, putative	1	iCn3D view
PF3D7_0827800	Q8IBB0	SET domain protein, putative	22	iCn3D view
PF3D7_0827900	C0H4Y6	protein disulfide-isomerase	6	iCn3D view
PF3D7_0828000	A0A5K1K8S0	rhomboid protease ROM3	1	iCn3D view
PF3D7_0828300	Q8IBB4	conserved protein, unknown function	6	iCn3D view
PF3D7_0828500	Q8IBB6	translation initiation factor eIF-2B subunit alpha, putative	1	iCn3D view
PF3D7_0828600	Q8IBB7	folate transporter 1	2	iCn3D view
PF3D7_0828800	Q8IBB9	GPI-anchored micronemal antigen	1	iCn3D view
PF3D7_0828900	Q8IBC0	conserved protein, unknown function	1	iCn3D view
PF3D7_0829000	Q8IBC1	conserved Plasmodium membrane protein, unknown function	9	iCn3D view
PF3D7_0829400	C0H4Z0	prolyl 4-hydroxylase subunit alpha, putative	5	iCn3D view
PF3D7_0830800	Q8IBD4	surface-associated interspersed protein 8.2 (SURFIN 8.2)	1	iCn3D view
PF3D7_0831000	Q8IBD6	Plasmodium exported protein (PHISTb), unknown function	1	iCn3D view
PF3D7_0831400	C0H4Z7	Plasmodium exported protein, unknown function	1	iCn3D view
PF3D7_0831500	A0A143ZW02	Plasmodium exported protein (PHIST), unknown function	1	iCn3D view
PF3D7_0831600	A0A143ZVM1	cytoadherence linked asexual protein 8	6	iCn3D view
PF3D7_0831700	K7NTP5	heat shock protein 70	8	iCn3D view
PF3D7_0901800	C0H512	Plasmodium exported protein, unknown function	1	iCn3D view
PF3D7_0902100	Q8I3C7	serine/threonine protein kinase, FIKK family	1	iCn3D view
PF3D7_0902200	C0H514	serine/threonine protein kinase, FIKK family	2	iCn3D view
PF3D7_0902800	Q8I3C0	serine repeat antigen 9	1	iCn3D view
PF3D7_0903100	Q8I3B7	protein RER1, putative	2	iCn3D view
PF3D7_0903200	C0H516	ras-related protein RAB7	4	iCn3D view

PF3D7_0903400	Q8I3B4	ATP-dependent RNA helicase DDX60, putative	15	iCn3D view
PF3D7_0903500	Q8I3B3	nucleoporin NUP138, putative	30	iCn3D view
PF3D7_0903600	A0A143ZVB6	conserved protein, unknown function	6	iCn3D view
PF3D7_0903700	Q6ZLZ9	alpha tubulin 1	11	iCn3D view
PF3D7_0903900	Q8I3B0	60S ribosomal protein L32	1	iCn3D view
PF3D7_0904000	Q8I3A9	GTPase-activating protein, putative	2	iCn3D view
PF3D7_0904100	Q8I3A8	AP-4 complex subunit epsilon, putative	18	iCn3D view
PF3D7_0904600	Q8I3A3	ubiquitin specific protease, putative	24	iCn3D view
PF3D7_0904800	Q8I3A1	replication protein A1, small fragment	11	iCn3D view
PF3D7_0905100	Q8I398	nucleoporin NUP221, putative	9	iCn3D view
PF3D7_0905300	Q8I396	dynein heavy chain, putative	3	iCn3D view
PF3D7_0905600	Q8I393	WD repeat-containing protein 66, putative	1	iCn3D view
PF3D7_0905700	A0A143ZWJ2	autophagy-related protein 3, putative	3	iCn3D view
PF3D7_0905800	Q8I391	conserved Plasmodium protein, unknown function	15	iCn3D view
PF3D7_0905900	Q8I390	coatomer subunit beta, putative	3	iCn3D view
PF3D7_0906100	Q8I388	vacuolar protein sorting-associated protein 46, putative	3	iCn3D view
PF3D7_0906300	Q8I386	Maf-like protein, putative	3	iCn3D view
PF3D7_0906400	Q8I385	dynein intermediate light chain, putative	2	iCn3D view
PF3D7_0906500	Q8I384	arginase	2	iCn3D view
PF3D7_0906700	C0H521	leucine-rich repeat protein	2	iCn3D view
PF3D7_0906890	A0A146M145	conserved Plasmodium protein, unknown function	1	iCn3D view
PF3D7_0907100	Q8I380	conserved Plasmodium protein, unknown function	2	iCn3D view
PF3D7_0907200	Q8I379	GTPase-activating protein, putative	18	iCn3D view
PF3D7_0907300	Q8I378	prefoldin-like protein, putative	4	iCn3D view
PF3D7_0907600	Q8I375	translation initiation factor SUI1, putative	4	iCn3D view

PF3D7_0908200	Q8I369	conserved Plasmodium protein, unknown function	3	iCn3D view
PF3D7_0908300	C0H526	conserved protein, unknown function	3	iCn3D view
PF3D7_0908500	C0H527	conserved Plasmodium protein, unknown function	4	iCn3D view
PF3D7_0908600	Q8I365	ribosomal RNA methyltransferase, putative	4	iCn3D view
PF3D7_0908800	Q8I364	mitochondrial carrier protein, putative	1	iCn3D view
PF3D7_0909000	Q8I362	conserved Plasmodium protein, unknown function	3	iCn3D view
PF3D7_0909300	Q8I359	apoptosis-related protein	1	iCn3D view
PF3D7_0909400	Q8I358	exoribonuclease, putative	4	iCn3D view
PF3D7_0909700	Q8I355	FHA domain protein, putative	8	iCn3D view
PF3D7_0909900	Q8I353	helicase SKI2W, putative	4	iCn3D view
PF3D7_0910100	C0H530	exportin-7, putative	5	iCn3D view
PF3D7_0910300	Q8I349	conserved protein, unknown function	1	iCn3D view
PF3D7_0910400	Q8I348	selenide water dikinase, putative	3	iCn3D view
PF3D7_0910500	Q8I347	DNA repair protein REV1, putative	6	iCn3D view
PF3D7_0910600	Q8I346	SNARE protein	1	iCn3D view
PF3D7_0910800	Q8I344	cytosolic Fe-S cluster assembly factor NBP35, putative	6	iCn3D view
PF3D7_0910900	C0H531	DNA primase large subunit, putative	1	iCn3D view
PF3D7_0911100	Q8I341	START domain-containing protein, putative	41	iCn3D view
PF3D7_0911400	Q8I338	conserved Plasmodium protein, unknown function	2	iCn3D view
PF3D7_0911900	Q8I333	falstatin	3	iCn3D view
PF3D7_0912000	Q8I332	conserved Plasmodium protein, unknown function	11	iCn3D view
PF3D7_0912100	Q8I331	zinc finger protein, putative	3	iCn3D view
PF3D7_0912400	Q8I328	alkaline phosphatase, putative	1	iCn3D view
PF3D7_0912500	C0H535	SAP domain-containing protein, putative	13	iCn3D view
PF3D7_0912600	Q8I326	conserved Plasmodium protein, unknown function	1	iCn3D view

PF3D7_0912900	Q8I323	26S proteasome regulatory subunit RPN8, putative	2	iCn3D view
PF3D7_0913000	Q8I322	conserved protein, unknown function	1	iCn3D view
PF3D7_0913200	Q8I320	elongation factor 1-beta	12	iCn3D view
PF3D7_0913300	Q8I319	conserved protein, unknown function	8	iCn3D view
PF3D7_0913800	Q8I314	conserved Plasmodium protein, unknown function	1	iCn3D view
PF3D7_0913900	Q8I313	arginine--tRNA ligase, putative	4	iCn3D view
PF3D7_0914100	C0H536	conserved Plasmodium protein, unknown function	8	iCn3D view
PF3D7_0914200	Q8I310	phospholipid or glycerol acyltransferase, putative	2	iCn3D view
PF3D7_0914300	C0H537	met-10+ like protein, putative	4	iCn3D view
PF3D7_0914400	Q8I308	protein KIC3	16	iCn3D view
PF3D7_0914600	Q8I306	transcription elongation factor 1, putative	3	iCn3D view
PF3D7_0914700	Q8I305	major facilitator superfamily-related transporter, putative	14	iCn3D view
PF3D7_0914900	Q8I303	BSD-domain protein, putative	10	iCn3D view
PF3D7_0915200	Q8I300	ribonuclease H2 subunit C, putative	2	iCn3D view
PF3D7_0915400	Q8I2Z8	ATP-dependent 6-phosphofructokinase	21	iCn3D view
PF3D7_0916000	Q8I2Z2	major facilitator superfamily domain-containing protein, putative	22	iCn3D view
PF3D7_0916200	Q8I2Z0	mitochondrial ribonuclease P catalytic subunit, putative	2	iCn3D view
PF3D7_0916400	C0H539	conserved Plasmodium protein, unknown function	18	iCn3D view
PF3D7_0916600	Q8I2Y6	methyltransferase, putative	1	iCn3D view
PF3D7_0917000	Q8I2Y3	merozoite organizing protein	21	iCn3D view
PF3D7_0917100	Q8I2Y2	N-glycosylase/DNA lyase, putative	1	iCn3D view
PF3D7_0917600	Q8I2X7	pre-mRNA-splicing factor ATP-dependent RNA helicase PRP43, putative	5	iCn3D view
PF3D7_0917700	Q8I2X6	GPN-loop GTPase, putative	1	iCn3D view
PF3D7_0917800	Q8I2X5	conserved Plasmodium protein, unknown function	3	iCn3D view
PF3D7_0917900	Q8I2X4	heat shock protein 70	8	iCn3D view

PF3D7_0918000	Q8I2X3	glideosome-associated protein 50	1	iCn3D view
PF3D7_0918300	Q8I2X0	eukaryotic translation initiation factor 3 subunit F, putative	2	iCn3D view
PF3D7_0918400	C0H544	conserved Plasmodium protein, unknown function	1	iCn3D view
PF3D7_0918600	Q8I2W7	ATP-dependent DNA helicase Q1	4	iCn3D view
PF3D7_0918700	Q8I2W6	conserved Plasmodium protein, unknown function	27	iCn3D view
PF3D7_0918800	Q8I2W5	dihydrouridine synthase, putative	1	iCn3D view
PF3D7_0918900	Q8I2W4	gamma-glutamylcysteine synthetase	14	iCn3D view
PF3D7_0919000	Q8I2W3	nucleosome assembly protein	10	iCn3D view
PF3D7_0919100	Q8I2W2	DnaJ protein, putative	5	iCn3D view
PF3D7_0919400	Q8I2V9	protein disulfide-isomerase PDI-Trans	1	iCn3D view
PF3D7_0919700	Q8I2V6	pyridoxal phosphate homeostasis protein, putative	2	iCn3D view
PF3D7_0919900	Q8I2V4	regulator of chromosome condensation-PP1-interacting protein	27	iCn3D view
PF3D7_0920000	C0H545	elongation of fatty acids protein, putative	2	iCn3D view
PF3D7_0920200	Q8I2V1	CS domain protein, putative	1	iCn3D view
PF3D7_0920400	Q8I2U9	conserved Plasmodium protein, unknown function	7	iCn3D view
PF3D7_0920700	Q8I2U6	CRAL/TRIO domain-containing protein, putative	9	iCn3D view
PF3D7_0920800	Q8I2U5	inosine-5'-monophosphate dehydrogenase	3	iCn3D view
PF3D7_0920900	Q8I2U4	U4/U6 snRNA-associated-splicing factor, putative	4	iCn3D view
PF3D7_0921000	Q8I2U3	ubiquitin-conjugating enzyme E2, putative	1	iCn3D view
PF3D7_0921200	C0H548	conserved Plasmodium membrane protein, unknown function	8	iCn3D view
PF3D7_0921600	Q8I2T7	tetratricopeptide repeat protein, putative	9	iCn3D view
PF3D7_0921800	C0H549	ribosome production factor 1, putative	1	iCn3D view
PF3D7_0921900	Q8I2T4	conserved Plasmodium protein, unknown function	4	iCn3D view
PF3D7_0922000	Q8I2T3	dynein intermediate chain, putative	2	iCn3D view
PF3D7_0922100	Q8I2T2	ubiquitin-like protein, putative	49	iCn3D view

PF3D7_0922200	Q7K6A4	S-adenosylmethionine synthetase	17	iCn3D view
PF3D7_0922400	Q8I2T0	para-aminobenzoic acid synthetase	1	iCn3D view
PF3D7_0922500	P27362	phosphoglycerate kinase	19	iCn3D view
PF3D7_0922600	C0H551	glutamine synthetase, putative	6	iCn3D view
PF3D7_0922700	Q8I2S9	pre-mRNA-splicing factor 18, putative	4	iCn3D view
PF3D7_0922800	C0H552	conserved Plasmodium protein, unknown function	17	iCn3D view
PF3D7_0922900	Q8I2S7	3-oxoacyl-[acyl-carrier-protein] reductase	1	iCn3D view
PF3D7_0923000	Q8I2S6	DNA-directed RNA polymerase II subunit RPB3, putative	2	iCn3D view
PF3D7_0923400	Q8I2S2	conserved Plasmodium protein, unknown function	27	iCn3D view
PF3D7_0923500	Q8I2S1	cyclin-dependent kinases regulatory subunit, putative	1	iCn3D view
PF3D7_0923900	Q8I2R8	polyadenylate-binding protein 2, putative	6	iCn3D view
PF3D7_0924000	Q8I2R7	patatin-like phospholipase, putative	16	iCn3D view
PF3D7_0924100	Q8I2R6	conserved Plasmodium protein, unknown function	8	iCn3D view
PF3D7_0924200	Q8I2R5	heptatricopeptide repeat-containing protein, putative	1	iCn3D view
PF3D7_0924400	Q8I2R3	conserved Plasmodium protein, unknown function	29	iCn3D view
PF3D7_0924600	Q8I2R1	conserved Plasmodium protein, unknown function	11	iCn3D view
PF3D7_0924700	Q8I2R0	splicing factor 3A subunit 3, putative	1	iCn3D view
PF3D7_0925600	Q8I2Q2	zinc binding protein (Yippee), putative	2	iCn3D view
PF3D7_0925700	Q7K6A1	histone deacetylase 1	9	iCn3D view
PF3D7_0925800	Q8I2Q1	regulator of nonsense transcripts 2, putative	3	iCn3D view
PF3D7_0926000	Q8I2P9	protein kinase, putative	1	iCn3D view
PF3D7_0926400	C0H564	monocarboxylate transporter, putative	10	iCn3D view
PF3D7_0926500	Q8I2P4	tetratricopeptide repeat protein, putative	13	iCn3D view
PF3D7_0926700	Q8I2P2	glutamine-dependent NAD(+) synthetase, putative	2	iCn3D view
PF3D7_0926800	Q8I2P1	conserved Plasmodium protein, unknown function	14	iCn3D view

PF3D7_0926900	Q8I2P0	replication termination factor, putative	2	iCn3D view
PF3D7_0927200	C0H566	gametocyte development protein GD1, putative	15	iCn3D view
PF3D7_0928100	Q8I2M8	conserved Plasmodium protein, unknown function	4	iCn3D view
PF3D7_0928200	Q8I2M7	conserved Plasmodium protein, unknown function	3	iCn3D view
PF3D7_0928300	C0H569	conserved Plasmodium protein, unknown function	1	iCn3D view
PF3D7_0928400	Q8I2M5	conserved protein, unknown function	3	iCn3D view
PF3D7_0928500	Q8I2M4	conserved Plasmodium protein, unknown function	1	iCn3D view
PF3D7_0928800	Q8I0W2	serine/threonine protein kinase, putative	3	iCn3D view
PF3D7_0928900	Q8I2M1	guanylate kinase	2	iCn3D view
PF3D7_0929000	Q8I2M0	transcription initiation factor TFIID subunit 7, putative	2	iCn3D view
PF3D7_0929100	Q8I2L9	conserved protein, unknown function	1	iCn3D view
PF3D7_0929200	C0H570	RNA-binding protein, putative	3	iCn3D view
PF3D7_0929700	A0A143ZXG1	conserved Plasmodium protein, unknown function	1	iCn3D view
PF3D7_0930400	A0A143ZVC5	zinc finger protein, putative	6	iCn3D view
PF3D7_0930500	Q8I2K9	diacylglycerol kinase, putative	19	iCn3D view
PF3D7_0930600	Q8I2K8	peptidyl-prolyl cis-trans isomerase	11	iCn3D view
PF3D7_0930800	C0H577	regulator of chromosome condensation, putative	45	iCn3D view
PF3D7_0931100	Q8I2K4	nucleolar protein Nop52, putative	4	iCn3D view
PF3D7_0931400	C0H579	PUB domain-containing protein, putative	6	iCn3D view
PF3D7_0931500	C0H580	conserved Plasmodium protein, unknown function	1	iCn3D view
PF3D7_0932000	C0H583	conserved Plasmodium protein, unknown function	9	iCn3D view
PF3D7_0932100	C0H584	protein MAM3, putative	19	iCn3D view
PF3D7_0932200	Q8I2J4	profilin	5	iCn3D view
PF3D7_0932300	Q8I2J3	M18 aspartyl aminopeptidase	4	iCn3D view
PF3D7_0932800	Q8I2I8	importin alpha re-exporter, putative	5	iCn3D view

PF3D7_0933000	Q8I0W1	CSTF domain-containing protein, putative	6	iCn3D view
PF3D7_0933100	C0H585	conserved Plasmodium protein, unknown function	3	iCn3D view
PF3D7_0933200	C0H586	calcyclin-binding protein, putative	4	iCn3D view
PF3D7_0933500	Q8I2I3	gamma-tubulin complex component, putative	21	iCn3D view
PF3D7_0933700	C0H589	conserved Plasmodium protein, unknown function	1	iCn3D view
PF3D7_0933900	Q8I2H9	Fip1 domain-containing protein, putative	3	iCn3D view
PF3D7_0934100	Q8I2H7	TFIIH basal transcription factor complex helicase XPD subunit	4	iCn3D view
PF3D7_0934500	Q8I2H3	V-type proton ATPase subunit E, putative	2	iCn3D view
PF3D7_0934700	Q8I2H1	UBX domain-containing protein, putative	4	iCn3D view
PF3D7_0934800	Q7K6A0	cAMP-dependent protein kinase catalytic subunit	10	iCn3D view
PF3D7_0934900	Q8I2H0	conserved Plasmodium protein, unknown function	1	iCn3D view
PF3D7_0935000	Q8I2G9	U2 small nuclear ribonucleoprotein B", putative	1	iCn3D view
PF3D7_0935200	Q8I2G8	vacuolar protein sorting-associated protein 33, putative	16	iCn3D view
PF3D7_0935400	Q8I2G6	gametocyte development protein 1	1	iCn3D view
PF3D7_0935500	C0H591	Plasmodium exported protein, unknown function	2	iCn3D view
PF3D7_0935600	Q8I2G4	gametocytogenesis-implicated protein	10	iCn3D view
PF3D7_0935800	Q8I2G2	cytoadherence linked asexual protein 9	3	iCn3D view
PF3D7_0935900	Q8I2G1	ring-exported protein 1	11	iCn3D view
PF3D7_0936600	Q8I2F4	gametocyte exported protein 5	4	iCn3D view
PF3D7_0936800	Q8I2F2	Plasmodium exported protein (PHISTc), unknown function	6	iCn3D view
PF3D7_1001000	Q8IK27	chondroitin sulfate A ligand	3	iCn3D view
PF3D7_1001500	Q8IK21	early transcribed membrane protein 10.1	3	iCn3D view
PF3D7_1001600	Q8IK20	exported lipase 2	15	iCn3D view
PF3D7_1001700	Q8IK19	Plasmodium exported protein (PHISTc), unknown function	1	iCn3D view
PF3D7_1001800	Q8IK18	Plasmodium exported protein (PHISTc), unknown function	2	iCn3D view

PF3D7_1001900	Q8IK17	Plasmodium exported protein (hyp16), unknown function	1	iCn3D view
PF3D7_1002000	Q8IK16	Plasmodium exported protein (hyp2), unknown function	2	iCn3D view
PF3D7_1002200	Q8IK14	tryptophan-rich antigen 3	1	iCn3D view
PF3D7_1002300	Q8IK13	conserved Plasmodium protein, unknown function	1	iCn3D view
PF3D7_1002400	Q8IK12	transformer-2 protein homolog beta, putative	22	iCn3D view
PF3D7_1002700	Q8IK09	conserved Plasmodium protein, unknown function	10	iCn3D view
PF3D7_1002800	Q8IK08	DnaJ protein, putative	7	iCn3D view
PF3D7_1002900	Q8IK07	conserved Plasmodium protein, unknown function	7	iCn3D view
PF3D7_1003000	Q8IK06	guanylate cyclase organizer UGO, putative	29	iCn3D view
PF3D7_1003400	Q8IK03	conserved Plasmodium protein, unknown function	8	iCn3D view
PF3D7_1003500	Q8IK02	40S ribosomal protein S20e, putative	2	iCn3D view
PF3D7_1003600	Q8IK01	inner membrane complex protein 1c, putative	27	iCn3D view
PF3D7_1003700	Q8IK00	MKT1 domain-containing protein, putative	10	iCn3D view
PF3D7_1003800	Q8IJZ9	U5 small nuclear ribonucleoprotein component, putative	2	iCn3D view
PF3D7_1004000	Q8IJZ7	60S ribosomal protein L13, putative	6	iCn3D view
PF3D7_1004400	Q8IJZ3	RNA-binding protein, putative	57	iCn3D view
PF3D7_1005100	Q8IJY7	U3 small nucleolar RNA-associated protein 25, putative	1	iCn3D view
PF3D7_1005500	A0A143ZY29	regulator of nonsense transcripts 1, putative	11	iCn3D view
PF3D7_1005600	C6S3C2	DnaJ protein, putative	11	iCn3D view
PF3D7_1006100	A0A143ZX72	CCR4-NOT transcription complex subunit 5, putative	1	iCn3D view
PF3D7_1006200	Q8IJX8	DNA/RNA-binding protein Alba 3	10	iCn3D view
PF3D7_1006700	Q8IJX4	conserved Plasmodium protein, unknown function	3	iCn3D view
PF3D7_1006800	Q8IJX3	G-strand-binding protein 2	9	iCn3D view
PF3D7_1006900	Q8IJX2	PPPDE peptidase, putative	3	iCn3D view
PF3D7_1007000	Q8IJX1	transmembrane protein 147, putative	2	iCn3D view

PF3D7_1007200	Q8IJX0	rho GTPase-activating protein, putative	12	iCn3D view
PF3D7_1007400	Q8IJW8	conserved protein, unknown function	5	iCn3D view
PF3D7_1007500	C6S3C5	conserved Plasmodium protein, unknown function	1	iCn3D view
PF3D7_1007700	Q8IJW6	AP2 domain transcription factor AP2-I	21	iCn3D view
PF3D7_1007900	Q8IJW4	eukaryotic translation initiation factor 3 subunit D, putative	12	iCn3D view
PF3D7_1008100	Q8IJW2	PHD finger protein PHD1	55	iCn3D view
PF3D7_1008400	Q8IJW0	26S protease regulatory subunit 4, putative	6	iCn3D view
PF3D7_1008500	Q8IJV9	protein GPR89, putative	19	iCn3D view
PF3D7_1008700	Q7KQL5	tubulin beta chain	21	iCn3D view
PF3D7_1008800	Q8IJV7	nucleolar protein 5, putative	10	iCn3D view
PF3D7_1008900	Q8IJV6	adenylate kinase	6	iCn3D view
PF3D7_1009200	Q8IJV3	ribonuclease, putative	1	iCn3D view
PF3D7_1009400	Q8IJV1	zinc finger protein, putative	6	iCn3D view
PF3D7_1009500	Q8IJV0	metalloprotease, putative	2	iCn3D view
PF3D7_1010100	Q8IJU4	PI31 domain-containing protein, putative	1	iCn3D view
PF3D7_1010300	Q8IJU2	succinate dehydrogenase subunit 4, putative	2	iCn3D view
PF3D7_1010600	Q8IJT9	eukaryotic translation initiation factor 2 subunit beta	3	iCn3D view
PF3D7_1010700	Q8IJT8	dolichyl-phosphate-mannose--protein mannosyltransferase, putative	1	iCn3D view
PF3D7_1010900	Q8IJT6	conserved Plasmodium protein, unknown function	1	iCn3D view
PF3D7_1011000	Q8IJT5	inner membrane complex sub-compartment protein 1	2	iCn3D view
PF3D7_1011100	Q8IJT4	conserved Plasmodium protein, unknown function	2	iCn3D view
PF3D7_1011200	Q8IJT3	BET1-like protein, putative	4	iCn3D view
PF3D7_1011400	Q8IJT1	proteasome subunit beta type-5	1	iCn3D view
PF3D7_1011800	Q8IJS7	PRE-binding protein	42	iCn3D view
PF3D7_1012000	Q8IJS5	RING zinc finger protein, putative	14	iCn3D view

PF3D7_1012400	Q8IJS1	hypoxanthine-guanine phosphoribosyltransferase	5	iCn3D view
PF3D7_1012500	Q8IJS0	phosphoglucomutase, putative	1	iCn3D view
PF3D7_1012600	Q8IJR9	GMP synthase [glutamine-hydrolyzing]	3	iCn3D view
PF3D7_1012700	Q8IJR8	NLI interacting factor-like phosphatase, putative	20	iCn3D view
PF3D7_1012900	Q8IJR6	autophagy-related protein 18	5	iCn3D view
PF3D7_1013100	Q8IJR4	U3 small nucleolar RNA-associated protein 13, putative	5	iCn3D view
PF3D7_1013200	Q8IJR3	conserved Plasmodium protein, unknown function	24	iCn3D view
PF3D7_1013500	Q8IJR0	phosphoinositide-specific phospholipase C	16	iCn3D view
PF3D7_1013600	Q8IJQ9	conserved Plasmodium protein, unknown function	16	iCn3D view
PF3D7_1014100	Q8IJQ4	merozoite surface protein MSA180	4	iCn3D view
PF3D7_1014300	Q8IJQ2	SPRY domain-containing protein, putative	13	iCn3D view
PF3D7_1014400	Q8IJQ1	MO15-related protein kinase	1	iCn3D view
PF3D7_1014800	Q8IJP7	EF hand domain-containing protein, putative	1	iCn3D view
PF3D7_1015100	Q8IJP4	CWC16 domain-containing protein, putative	2	iCn3D view
PF3D7_1015300	Q8IJP2	methionine aminopeptidase 1b, putative	4	iCn3D view
PF3D7_1015400	Q8IJP1	RMI1 domain-containing protein, putative	18	iCn3D view
PF3D7_1015600	Q8IJN9	heat shock protein 60	4	iCn3D view
PF3D7_1015900	Q8IJN7	enolase	5	iCn3D view
PF3D7_1016000	Q8IJN6	conserved Plasmodium protein, unknown function	15	iCn3D view
PF3D7_1016200	Q8IJN4	Rab3 GTPase-activating protein non-catalytic subunit, putative	1	iCn3D view
PF3D7_1016400	Q8IJN3	serine/threonine protein kinase, FIKK family	2	iCn3D view
PF3D7_1016500	Q8IJN2	Plasmodium exported protein (PHISTc), unknown function	2	iCn3D view
PF3D7_1016700	Q8IJN1	Plasmodium exported protein (PHISTc), unknown function	6	iCn3D view
PF3D7_1016900	Q8IJM9	early transcribed membrane protein 10.3	3	iCn3D view
PF3D7_1017000	Q7KQL4	DNA polymerase delta catalytic subunit	1	iCn3D view

PF3D7_1017300	Q8IJM6	golgi re-assembly stacking protein 2	8	iCn3D view
PF3D7_1017500	Q8IJM4	myosin essential light chain ELC	1	iCn3D view
PF3D7_1017600	Q8IJM3	conserved Plasmodium protein, unknown function	20	iCn3D view
PF3D7_1017900	Q8IJM0	26S proteasome regulatory subunit p55, putative	1	iCn3D view
PF3D7_1018200	C6S3C9	serine/threonine protein phosphatase 8, putative	58	iCn3D view
PF3D7_1018600	Q8IJL6	tRNA wybutosine-synthesizing protein, putative	13	iCn3D view
PF3D7_1018800	Q8IJL4	conserved protein, unknown function	7	iCn3D view
PF3D7_1019100	Q8IJL1	conserved Plasmodium protein, unknown function	32	iCn3D view
PF3D7_1019300	A0A144A1F0	zinc finger protein, putative	11	iCn3D view
PF3D7_1019400	Q8IJK8	60S ribosomal protein L30e, putative	3	iCn3D view
PF3D7_1019500	A0A143ZZS2	TMEM94 domain-containing protein, putative	1	iCn3D view
PF3D7_1019600	Q8IJK6	conserved Plasmodium protein, unknown function	16	iCn3D view
PF3D7_1019700	Q8IJK5	conserved Plasmodium protein, unknown function	14	iCn3D view
PF3D7_1019900	Q8IJK2	autophagy-related protein 8	6	iCn3D view
PF3D7_1020000	Q8IJK1	RNA-binding protein 34, putative	3	iCn3D view
PF3D7_1020300	Q8IJJ9	cytoplasmic dynein intermediate chain, putative	4	iCn3D view
PF3D7_1020400	Q8IJJ8	rRNA (cytosine-C(5))-methyltransferase, putative	1	iCn3D view
PF3D7_1020600	Q8IJJ6	conserved Plasmodium membrane protein, unknown function	1	iCn3D view
PF3D7_1020700	Q8IJJ5	N-acetyltransferase, GNAT family, putative	8	iCn3D view
PF3D7_1020900	Q7KQL3	ADP-ribosylation factor 1	5	iCn3D view
PF3D7_1021100	Q8IJJ2	conserved Plasmodium protein, unknown function	5	iCn3D view
PF3D7_1021200	Q8IJJ1	conserved Plasmodium protein, unknown function	8	iCn3D view
PF3D7_1021400	A0A143ZZU2	endomembrane protein 70, putative	1	iCn3D view
PF3D7_1021900	Q8IJI4	PHAX domain-containing protein, putative	22	iCn3D view
PF3D7_1022000	Q8IJI3	RNA-binding protein UIS12, putative	6	iCn3D view

PF3D7_1022200	Q8IJI2	folate-biopterin transporter, putative	1	iCn3D view
PF3D7_1022300	Q8IJI1	ZIP domain-containing protein, putative	2	iCn3D view
PF3D7_1022400	Q8IJI0	serine/arginine-rich splicing factor 4	54	iCn3D view
PF3D7_1022600	Q8IJH9	kelch protein K10	5	iCn3D view
PF3D7_1022700	Q8IJH8	phospholipid scramblase	1	iCn3D view
PF3D7_1023000	Q8IJH5	conserved Plasmodium protein, unknown function	7	iCn3D view
PF3D7_1023100	Q8IJH4	dynein heavy chain, putative	3	iCn3D view
PF3D7_1023600	Q8IJG9	conserved protein, unknown function	1	iCn3D view
PF3D7_1023700	Q8IJG8	conserved Plasmodium protein, unknown function	1	iCn3D view
PF3D7_1023900	Q8IJG6	chromodomain-helicase-DNA-binding protein 1 homolog, putative	54	iCn3D view
PF3D7_1024800	Q8IJF6	exported protein 3	11	iCn3D view
PF3D7_1025000	Q8IJF4	Eps15-like protein	38	iCn3D view
PF3D7_1025400	Q8IJF0	conserved Plasmodium membrane protein, unknown function	6	iCn3D view
PF3D7_1025500	Q8IJE8	dynein, putative	5	iCn3D view
PF3D7_1025600	Q8IJE6	cytochrome c oxidase copper chaperone, putative	1	iCn3D view
PF3D7_1026000	Q8IJE1	conserved Plasmodium protein, unknown function	6	iCn3D view
PF3D7_1026300	Q8IJD8	conserved Plasmodium protein, unknown function	1	iCn3D view
PF3D7_1026800	Q8IJD4	40S ribosomal protein S2	5	iCn3D view
PF3D7_1027100	Q8IJD2	U3 small nucleolar ribonucleoprotein protein MPP10, putative	10	iCn3D view
PF3D7_1027300	Q8IJD0	peroxiredoxin	14	iCn3D view
PF3D7_1027700	A0A143ZXT2	centrin-3	10	iCn3D view
PF3D7_1027800	Q8IJC6	60S ribosomal protein L3	11	iCn3D view
PF3D7_1028000	Q8IJC4	methyltransferase, putative	1	iCn3D view
PF3D7_1028200	Q8IJC2	RING zinc finger protein, putative	1	iCn3D view
PF3D7_1028300	Q8IJC1	rRNA-processing protein EBP2, putative	2	iCn3D view

PF3D7_1028400	Q8IJC0	ribosome biogenesis protein RPF2, putative	2	iCn3D view
PF3D7_1028500	Q8IJB9	partial CSTF domain-containing protein, putative	3	iCn3D view
PF3D7_1028600	Q8IJB8	conserved Plasmodium protein, unknown function	8	iCn3D view
PF3D7_1028700	Q8IJB7	merozoite TRAP-like protein	2	iCn3D view
PF3D7_1029200	Q8IJB3	WD repeat-containing protein, putative	2	iCn3D view
PF3D7_1029300	Q8IJB2	conserved protein, unknown function	1	iCn3D view
PF3D7_1029400	Q8IJB1	cell division control protein CDC50C	12	iCn3D view
PF3D7_1029600	Q8IJA9	adenosine deaminase	2	iCn3D view
PF3D7_1029900	Q8IJA6	ORC3 domain-containing protein, putative	17	iCn3D view
PF3D7_1030000	Q8IJA5	transcription elongation factor SPT4, putative	6	iCn3D view
PF3D7_1030100	Q8IJA4	pre-mRNA-splicing factor ATP-dependent RNA helicase PRP22, putative	11	iCn3D view
PF3D7_1030200	Q8IJA3	claudin-like apicomplexan microneme protein, putative	7	iCn3D view
PF3D7_1030300	Q8IJA2	conserved Plasmodium protein, unknown function	4	iCn3D view
PF3D7_1030600	Q8IJ99	tRNA N6-adenosine threonylcarbamoyltransferase	2	iCn3D view
PF3D7_1030700	Q8IJ98	RNA methyltransferase, putative	1	iCn3D view
PF3D7_1031200	Q8IJ93	MORN repeat-containing protein 1	1	iCn3D view
PF3D7_1031400	A0A144A039	OTU-like cysteine protease	9	iCn3D view
PF3D7_1031500	Q8IJ90	ATP-dependent DNA helicase DDX3X	6	iCn3D view
PF3D7_1031600	Q8IJ89	protein GEXP15	9	iCn3D view
PF3D7_1031800	Q8IJ87	conserved protein, unknown function	4	iCn3D view
PF3D7_1032100	Q8IJ85	mRNA-decapping enzyme subunit 1, putative	22	iCn3D view
PF3D7_1032500	Q8IJ82	DER1-like protein, putative	2	iCn3D view
PF3D7_1032700	Q8IJ80	conserved Plasmodium protein, unknown function	8	iCn3D view
PF3D7_1032800	C6S3E1	leucine-rich repeat protein 1	5	iCn3D view
PF3D7_1033200	Q8IJ76	early transcribed membrane protein 10.2	19	iCn3D view

PF3D7_1033300	Q8IJ75	conserved protein, unknown function	3	iCn3D view
PF3D7_1033400	Q8IJ74	haloacid dehalogenase-like hydrolase	8	iCn3D view
PF3D7_1033500	Q8IJ73	WD repeat-containing protein 70, putative	8	iCn3D view
PF3D7_1033700	Q8IJ72	bromodomain protein 1	15	iCn3D view
PF3D7_1033900	Q8IJ70	ubiquitin-conjugating enzyme E2, putative	2	iCn3D view
PF3D7_1034000	Q8IJ69	Sec1 family protein, putative	3	iCn3D view
PF3D7_1034500	Q8IJ65	armadillo repeat protein, putative	2	iCn3D view
PF3D7_1034900	Q8IJ60	methionine--tRNA ligase	3	iCn3D view
PF3D7_1035100	Q8IJ58	probable protein, unknown function	2	iCn3D view
PF3D7_1035300	Q8IJ56	glutamate-rich protein GLURP	12	iCn3D view
PF3D7_1035400	Q8IJ55	merozoite surface protein 3	4	iCn3D view
PF3D7_1035500	Q8IJ54	merozoite surface protein 6	2	iCn3D view
PF3D7_1035700	Q8IJ52	duffy binding-like merozoite surface protein	2	iCn3D view
PF3D7_1035800	Q8IJ50	probable protein, unknown function	1	iCn3D view
PF3D7_1035900	Q8IJ49	merozoites-associated armadillo repeats protein	1	iCn3D view
PF3D7_1036700	A0A143ZZR4	phosducin-like protein 2, putative	3	iCn3D view
PF3D7_1036800	Q8IJ40	acetyl-CoA transporter 1, putative	4	iCn3D view
PF3D7_1037300	Q8IJ34	ADP,ATP carrier protein 1	1	iCn3D view
PF3D7_1037500	Q8IJ32	dynamin-like protein	5	iCn3D view
PF3D7_1037600	Q8IJ31	TFIIH basal transcription factor complex helicase XPB subunit, putative	4	iCn3D view
PF3D7_1038100	Q8IJ27	GDP dissociation inhibitor, putative	3	iCn3D view
PF3D7_1038800	Q8IJ23	RESA-like protein with PHIST and DnaJ domains	1	iCn3D view
PF3D7_1039000	Q8IJ21	serine/threonine protein kinase, FIKK family	8	iCn3D view
PF3D7_1102500	Q8IIX5	gametocyte exported protein 2	18	iCn3D view
PF3D7_1102800	Q8IIX3	early transcribed membrane protein 11.2	1	iCn3D view

PF3D7_1103100	Q8IIX0	60S acidic ribosomal protein P1, putative	2	iCn3D view
PF3D7_1103400	Q8IIW9	iron-sulfur cluster assembly protein SufD	1	iCn3D view
PF3D7_1103600	Q8IIW6	actin-like protein, putative	2	iCn3D view
PF3D7_1103700	Q8IIW5	casein kinase II beta chain	9	iCn3D view
PF3D7_1104000	Q8IIW2	phenylalanine--tRNA ligase beta subunit	6	iCn3D view
PF3D7_1104100	Q8IIW1	syntaxin, Qa-SNARE family	17	iCn3D view
PF3D7_1104200	Q8IIW0	chromatin remodeling protein	29	iCn3D view
PF3D7_1104300	Q8IIV9	conserved Plasmodium protein, unknown function	13	iCn3D view
PF3D7_1104400	Q8IIV8	thioredoxin-like mero protein	1	iCn3D view
PF3D7_1104500	Q8IIV7	WD repeat-containing protein, putative	3	iCn3D view
PF3D7_1105000	Q8IIV2	histone H4	4	iCn3D view
PF3D7_1105100	Q8IIV1	histone H2B	12	iCn3D view
PF3D7_1105300	Q8IIU9	conserved Plasmodium protein, unknown function	3	iCn3D view
PF3D7_1105400	Q8IIU8	40S ribosomal protein S4, putative	6	iCn3D view
PF3D7_1105500	Q8I714	centrin-4	5	iCn3D view
PF3D7_1105800	Q8IIU5	conserved Apicomplexan protein, unknown function	3	iCn3D view
PF3D7_1106000	Q8IIU3	RuvB-like helicase 2	1	iCn3D view
PF3D7_1106200	Q8IIU1	conserved Plasmodium protein, unknown function	1	iCn3D view
PF3D7_1106500	Q8IIT8	conserved Plasmodium protein, unknown function	2	iCn3D view
PF3D7_1106700	Q8IIT6	DNA replication ATP-dependent helicase/nuclease DNA2, putative	6	iCn3D view
PF3D7_1106800	Q8IIT5	pseudo-tyrosine kinase-like protein	9	iCn3D view
PF3D7_1107100	Q8IIT2	nucleic acid binding protein, putative	7	iCn3D view
PF3D7_1107200	Q8IIT1	vacuolar fusion protein CCZ1, putative	4	iCn3D view
PF3D7_1107600	Q8IIS6	coiled-coil domain-containing protein 40, putative	4	iCn3D view
PF3D7_1107700	Q8IIS5	pescadillo homolog	1	iCn3D view

PF3D7_1107900	Q8IIS3	mechanosensitive ion channel protein, putative	4	iCn3D view
PF3D7_1108100	Q8IIS1	conserved Plasmodium protein, unknown function	10	iCn3D view
PF3D7_1108400	Q8IIR9	casein kinase 2, alpha subunit	2	iCn3D view
PF3D7_1108600	Q8IIR7	endoplasmic reticulum-resident calcium binding protein	10	iCn3D view
PF3D7_1108700	Q8IIR6	heat shock protein J2	1	iCn3D view
PF3D7_1109400	Q8IIR2	essential nuclear protein 1, putative	2	iCn3D view
PF3D7_1109900	Q8I713	60S ribosomal protein L36	5	iCn3D view
PF3D7_1110100	A0A143ZYA8	C2 domain-containing membrane protein	4	iCn3D view
PF3D7_1110200	Q8IIR0	pre-mRNA-processing factor 6, putative	28	iCn3D view
PF3D7_1110300	Q8IIQ9	G-patch domain-containing protein, putative	3	iCn3D view
PF3D7_1110400	Q8IIQ7	RNA-binding protein, putative	71	iCn3D view
PF3D7_1110500	Q8IIQ6	vacuolar protein sorting-associated protein 35, putative	1	iCn3D view
PF3D7_1110700	A0A143ZXR3	actin-like protein, putative	3	iCn3D view
PF3D7_1110900	Q8IIQ3	ES2 protein, putative	13	iCn3D view
PF3D7_1111100	Q8IIQ1	replication factor C subunit 5, putative	1	iCn3D view
PF3D7_1111200	Q8IIQ0	transcription elongation regulator 1-like protein, putative	6	iCn3D view
PF3D7_1111300	Q8IIP9	protein transport protein BOS1, putative	2	iCn3D view
PF3D7_1111500	C6S3F0	acylphosphatase, putative	2	iCn3D view
PF3D7_1111600	A0A143ZZC6	endonuclease/exonuclease/phosphatase family protein, putative	4	iCn3D view
PF3D7_1111700	Q8IIP6	conserved Plasmodium protein, unknown function	2	iCn3D view
PF3D7_1111800	Q8IIP5	peptidyl-prolyl cis-trans isomerase, putative	1	iCn3D view
PF3D7_1112400	Q8IIN9	nucleic acid binding protein, putative	1	iCn3D view
PF3D7_1112500	Q8IIN8	RNA transcription, translation and transport factor protein, putative	1	iCn3D view
PF3D7_1112600	Q8IIN7	ATPase, putative	8	iCn3D view
PF3D7_1112700	Q8IIN6	conserved Plasmodium protein, unknown function	1	iCn3D view

PF3D7_1113100	Q8IIN1	protein tyrosine phosphatase	1	iCn3D view
PF3D7_1113300	Q8IIM9	UDP-galactose transporter, putative	1	iCn3D view
PF3D7_1113400	Q8IIM8	ubiquitin domain-containing protein DSK2, putative	3	iCn3D view
PF3D7_1113600	Q8IIM6	conserved Plasmodium protein, unknown function	1	iCn3D view
PF3D7_1113800	Q8IIM4	conserved Plasmodium membrane protein, unknown function	4	iCn3D view
PF3D7_1113900	Q7KQK7	mitogen-activated protein kinase 2	3	iCn3D view
PF3D7_1114100	Q8IIM2	rhomboid protease ROM1	2	iCn3D view
PF3D7_1114400	Q8IIL8	RAP protein, putative	1	iCn3D view
PF3D7_1114500	Q8IIL7	conserved Plasmodium protein, unknown function	3	iCn3D view
PF3D7_1114700	Q8IIL5	cyclin-dependent-like kinase CLK3	5	iCn3D view
PF3D7_1115200	Q8IIL1	histone-lysine N-methyltransferase SET7	7	iCn3D view
PF3D7_1115400	Q8IIL0	cysteine proteinase falcipain 3	3	iCn3D view
PF3D7_1115800	Q8IIK7	conserved Plasmodium protein, unknown function	16	iCn3D view
PF3D7_1116000	Q8IIK5	rhoptry neck protein 4	2	iCn3D view
PF3D7_1116300	Q8IIK3	peptidyl-prolyl cis-trans isomerase	2	iCn3D view
PF3D7_1116400	Q8IIK2	guanine nucleotide-exchange factor SEC12	3	iCn3D view
PF3D7_1116500	Q8IIK1	folate transporter 2	4	iCn3D view
PF3D7_1116700	Q8IIJ9	dipeptidyl aminopeptidase 1	1	iCn3D view
PF3D7_1116800	Q8IIJ8	heat shock protein 101	2	iCn3D view
PF3D7_1116900	A0A144A0T9	conserved protein, unknown function	1	iCn3D view
PF3D7_1117400	Q8IIJ3	conserved Plasmodium protein, unknown function	1	iCn3D view
PF3D7_1117700	Q7KQK6	GTP-binding nuclear protein RAN/TC4	7	iCn3D view
PF3D7_1117800	Q8IIJ0	DNA mismatch repair protein MLH	1	iCn3D view
PF3D7_1117900	Q8III9	conserved Plasmodium protein, unknown function	21	iCn3D view
PF3D7_1118200	Q8III6	heat shock protein 90, putative	1	iCn3D view

PF3D7_1118300	Q8III5	insulinase, putative	4	iCn3D view
PF3D7_1118500	Q8III3	nucleolar protein 56, putative	12	iCn3D view
PF3D7_1118600	Q8III2	histone acetyltransferase MYST	5	iCn3D view
PF3D7_1118700	Q8III1	myosin light chain B	4	iCn3D view
PF3D7_1119000	Q8IIH7	acyl-CoA-binding protein, putative	5	iCn3D view
PF3D7_1119100	Q8IIH6	tRNA m(1)G methyltransferase, putative	2	iCn3D view
PF3D7_1119200	A0A143ZYQ2	conserved protein, unknown function	2	iCn3D view
PF3D7_1119300	Q8IIH4	splicing factor U2AF small subunit, putative	10	iCn3D view
PF3D7_1119400	Q8IIH3	ubiquitin-protein ligase, putative	2	iCn3D view
PF3D7_1119500	Q8IIH2	AP-4 complex subunit mu, putative	1	iCn3D view
PF3D7_1119700	Q8IIH0	conserved Plasmodium protein, unknown function	3	iCn3D view
PF3D7_1119800	Q8IIG9	alternative splicing factor ASF-1, putative	3	iCn3D view
PF3D7_1120000	Q8IIG7	conserved protein, unknown function	14	iCn3D view
PF3D7_1120100	Q8IIG6	phosphoglycerate mutase, putative	8	iCn3D view
PF3D7_1120400	Q8IIG3	alpha/beta hydrolase fold domain containing protein, putative	3	iCn3D view
PF3D7_1120500	Q8IIG2	tRNA nucleotidyltransferase, putative	1	iCn3D view
PF3D7_1120900	Q8IIF8	heat shock factor-binding protein 1	1	iCn3D view
PF3D7_1121000	Q8IIF7	palmitoyltransferase DHHC3	8	iCn3D view
PF3D7_1121100	Q8IIF6	conserved protein, unknown function	33	iCn3D view
PF3D7_1121300	Q8IIF4	tyrosine kinase-like protein	5	iCn3D view
PF3D7_1121400	Q8IIF2	WD repeat-containing protein, putative	3	iCn3D view
PF3D7_1121600	Q8IIF0	exported protein 1	5	iCn3D view
PF3D7_1121700	Q8IIE9	protein GCN20	7	iCn3D view
PF3D7_1121900	Q8IIE7	3-phosphoinositide-dependent protein kinase 1	4	iCn3D view
PF3D7_1122200	Q8IIE4	JmjC domain-containing protein 3	2	iCn3D view

PF3D7_1122300	Q8IIE3	conserved Plasmodium protein, unknown function	1	iCn3D view
PF3D7_1122400	Q8IIE2	conserved Plasmodium protein, unknown function	14	iCn3D view
PF3D7_1122800	Q8IID5	calcium-dependent protein kinase 6	7	iCn3D view
PF3D7_1123000	Q8IID3	conserved Plasmodium protein, unknown function	12	iCn3D view
PF3D7_1123100	Q8IID2	calcium-dependent protein kinase 7	27	iCn3D view
PF3D7_1123200	Q8IID1	leucine-rich repeat protein	2	iCn3D view
PF3D7_1123300	Q8IID0	RING zinc finger protein, putative	3	iCn3D view
PF3D7_1123400	Q8IIC9	eukaryotic peptide chain release factor GTP-binding subunit, putative	5	iCn3D view
PF3D7_1123500	Q8IIC8	golgi protein 2	2	iCn3D view
PF3D7_1123800	Q8IIC5	structural maintenance of chromosomes protein 5, putative	2	iCn3D view
PF3D7_1123900	Q8IIC4	13 kDa ribonucleoprotein-associated protein, putative	8	iCn3D view
PF3D7_1124100	Q8IIC2	BEACH domain-containing protein, putative	5	iCn3D view
PF3D7_1124200	A0A143ZYU5	thioredoxin-like protein, putative	2	iCn3D view
PF3D7_1124300	Q8IIC0	bromodomain protein 7	8	iCn3D view
PF3D7_1124400	Q8IIB9	U6 snRNA-associated Sm-like protein LSm1, putative	1	iCn3D view
PF3D7_1124600	Q8IIB7	ethanolamine kinase	9	iCn3D view
PF3D7_1124800	Q8IIB5	ribosome biogenesis regulatory protein, putative	2	iCn3D view
PF3D7_1125100	Q8IIB2	ATP synthase F0 subunit b-like protein, putative	1	iCn3D view
PF3D7_1125200	A0A143ZYV5	ubiquitin-like domain-containing protein, putative	1	iCn3D view
PF3D7_1125500	Q8IIA8	small nuclear ribonucleoprotein Sm D1, putative	2	iCn3D view
PF3D7_1125700	Q8IIA7	kelch domain-containing protein, putative	4	iCn3D view
PF3D7_1125800	Q8IIA6	kelch domain-containing protein, putative	9	iCn3D view
PF3D7_1126000	Q8IIA4	threonine--tRNA ligase	6	iCn3D view
PF3D7_1126100	Q8IIA3	autophagy-related protein 7, putative	2	iCn3D view
PF3D7_1126200	Q8IIA2	40S ribosomal protein S18, putative	4	iCn3D view

PF3D7_1126300	A0A143ZZE4	DnaJ protein, putative	4	iCn3D view
PF3D7_1126500	Q8II99	WD repeat-containing protein, putative	5	iCn3D view
PF3D7_1126900	Q8II94	small nuclear ribonucleoprotein F, putative	1	iCn3D view
PF3D7_1127000	Q8II93	protein phosphatase, putative	2	iCn3D view
PF3D7_1127300	Q8II90	tRNA (guanine-N(7)-)-methyltransferase, putative	1	iCn3D view
PF3D7_1127600	Q8II87	CRAL/TRIO domain-containing protein, putative	1	iCn3D view
PF3D7_1127700	Q8II86	conserved Plasmodium protein, unknown function	1	iCn3D view
PF3D7_1127800	Q8II85	TFIIS central domain-containing protein, putative	12	iCn3D view
PF3D7_1127900	Q8II84	conserved Plasmodium protein, unknown function	2	iCn3D view
PF3D7_1128000	Q8II83	conserved Plasmodium protein, unknown function	8	iCn3D view
PF3D7_1128100	Q8II82	prefoldin subunit 5, putative	9	iCn3D view
PF3D7_1128200	Q8II81	multiprotein-bridging factor 1, putative	1	iCn3D view
PF3D7_1128400	Q8II79	bifunctional farnesyl/geranylgeranyl diphosphate synthase	1	iCn3D view
PF3D7_1128500	Q8II78	Fe-S cluster assembly factor HCF101, putative	2	iCn3D view
PF3D7_1128600	Q8II77	CCR4-NOT transcription complex subunit 2, putative	2	iCn3D view
PF3D7_1128900	A0A143ZYW1	conserved protein, unknown function	5	iCn3D view
PF3D7_1129000	Q8II73	spermidine synthase	2	iCn3D view
PF3D7_1129100	Q8II72	parasitophorous vacuolar protein 1	6	iCn3D view
PF3D7_1129200	Q8II71	26S proteasome regulatory subunit RPN7, putative	1	iCn3D view
PF3D7_1129300	Q8II70	conserved Plasmodium protein, unknown function	9	iCn3D view
PF3D7_1129400	Q8II69	RNA cytosine C(5)-methyltransferase, putative	4	iCn3D view
PF3D7_1129500	A0A143ZYC4	A/G-specific adenine glycosylase, putative	2	iCn3D view
PF3D7_1129600	Q8II67	phosphatidylinositol-4-phosphate 5-kinase, putative	3	iCn3D view
PF3D7_1129800	Q8II65	conserved Plasmodium protein, unknown function	1	iCn3D view
PF3D7_1129900	Q8II64	major facilitator superfamily-related transporter, putative	23	iCn3D view

PF3D7_1130000	Q8II63	phosphoacetylglucosamine mutase, putative	3	iCn3D view
PF3D7_1130100	Q8II62	60S ribosomal protein L38	1	iCn3D view
PF3D7_1130200	Q8II61	60S ribosomal protein P0	7	iCn3D view
PF3D7_1130400	Q8II60	26S protease regulatory subunit 6A, putative	14	iCn3D view
PF3D7_1130500	Q8II59	conserved oligomeric Golgi complex subunit 2, putative	2	iCn3D view
PF3D7_1130700	Q8II57	structural maintenance of chromosomes protein 1, putative	11	iCn3D view
PF3D7_1131000	Q8II54	RNA-binding protein s1, putative	1	iCn3D view
PF3D7_1131300	Q8II51	conserved Plasmodium protein, unknown function	1	iCn3D view
PF3D7_1131600	Q8II48	kelch domain-containing protein, putative	4	iCn3D view
PF3D7_1131800	Q8II47	oxysterol-binding protein, putative	12	iCn3D view
PF3D7_1132000	Q8II45	ubiquitin-like protein, putative	6	iCn3D view
PF3D7_1132200	Q8II43	T-complex protein 1 subunit alpha	2	iCn3D view
PF3D7_1132300	Q8II42	nucleic acid binding protein, putative	6	iCn3D view
PF3D7_1132400	Q8II41	conserved Plasmodium membrane protein, unknown function	9	iCn3D view
PF3D7_1132600	Q8II38	pre-mRNA-splicing factor 38A, putative	9	iCn3D view
PF3D7_1133100	Q8II33	conserved Plasmodium protein, unknown function	2	iCn3D view
PF3D7_1133400	Q7KQK5	apical membrane antigen 1	3	iCn3D view
PF3D7_1133700	Q8II28	FHA domain-containing protein, putative	2	iCn3D view
PF3D7_1133800	Q8II27	RNA (uracil-5-)methyltransferase	13	iCn3D view
PF3D7_1134000	Q8II24	heat shock protein 70	1	iCn3D view
PF3D7_1134200	Q8II22	conserved Plasmodium protein, unknown function	2	iCn3D view
PF3D7_1134300	Q8II21	erythrocyte membrane and merozoite antigen EMMA1	52	iCn3D view
PF3D7_1134500	Q8II19	alpha/beta hydrolase, putative	2	iCn3D view
PF3D7_1134600	Q8II18	CCCH-type zinc finger protein ZNF4	4	iCn3D view
PF3D7_1134700	Q8II17	DNA-directed RNA polymerase I subunit RPA2, putative	2	iCn3D view

PF3D7_1134800	Q8II16	coatomer subunit delta	5	iCn3D view
PF3D7_1135100	Q8II13	protein phosphatase PPM8, putative	2	iCn3D view
PF3D7_1135600	Q8II07	condensin-2 complex subunit D3, putative	2	iCn3D view
PF3D7_1135900	Q8II05	3-oxo-5-alpha-steroid 4-dehydrogenase, putative	1	iCn3D view
PF3D7_1136000	Q8II04	conserved Plasmodium protein, unknown function	15	iCn3D view
PF3D7_1136300	Q8II01	tudor staphylococcal nuclease	11	iCn3D view
PF3D7_1136400	Q8II00	signal recognition particle subunit SRP72, putative	4	iCn3D view
PF3D7_1136500	Q8IHZ9	casein kinase 1	6	iCn3D view
PF3D7_1136700	Q8IHZ7	armadillo-interacting protein AIP	5	iCn3D view
PF3D7_1136800	Q8IHZ6	DnaJ protein, putative	2	iCn3D view
PF3D7_1136900	Q8IHZ5	subtilisin-like protease 2	5	iCn3D view
PF3D7_1137200	Q8IHZ3	apical exonemal protein	1	iCn3D view
PF3D7_1137700	Q8IHY7	calcium-binding protein, putative	2	iCn3D view
PF3D7_1137900	Q8IHY6	conserved Plasmodium protein, unknown function	2	iCn3D view
PF3D7_1138400	Q8IHY1	guanylyl cyclase alpha	84	iCn3D view
PF3D7_1138600	Q8IHX9	beta-catenin-like protein 1, putative	2	iCn3D view
PF3D7_1138700	Q8IHX8	protein KIC5	39	iCn3D view
PF3D7_1138800	Q8IHX6	WD repeat-containing protein, putative	18	iCn3D view
PF3D7_1139100	Q8IHX4	RNA-binding protein, putative	7	iCn3D view
PF3D7_1139300	Q8IHX2	AP2 domain transcription factor AP2-G5	13	iCn3D view
PF3D7_1139800	Q8IHW8	conserved Plasmodium protein, unknown function	5	iCn3D view
PF3D7_1140100	Q8IHW4	V-type proton ATPase subunit F, putative	1	iCn3D view
PF3D7_1140200	Q8IHW3	conserved Plasmodium protein, unknown function	13	iCn3D view
PF3D7_1140400	Q8IHW1	conserved Plasmodium protein, unknown function	4	iCn3D view
PF3D7_1140500	Q8IHW0	myosin K, putative	3	iCn3D view

PF3D7_1140600	Q8IHV9	conserved Plasmodium protein, unknown function	6	iCn3D view
PF3D7_1140700	Q8IHV8	chromo domain-containing protein CDP	12	iCn3D view
PF3D7_1140800	Q8IHV7	CID domain-containing protein, putative	5	iCn3D view
PF3D7_1140900	Q8IHV6	NdP1 protein, putative	4	iCn3D view
PF3D7_1141000	Q8IHV5	conserved Plasmodium protein, unknown function	1	iCn3D view
PF3D7_1141400	Q8IHV1	phosphatidylinositol N-acetylglucosaminyltransferase subunit H, putative	2	iCn3D view
PF3D7_1141700	Q8IHU8	OTU domain-containing protein, putative	2	iCn3D view
PF3D7_1141800	Q8IHU7	EELM2 domain-containing protein, putative	9	iCn3D view
PF3D7_1142300	Q8IHU2	conserved Plasmodium membrane protein, unknown function	20	iCn3D view
PF3D7_1142400	Q8IHU1	coproporphyrinogen-III oxidase	2	iCn3D view
PF3D7_1142500	Q8IHU0	60S ribosomal protein L28	8	iCn3D view
PF3D7_1142600	Q8IHT9	60S ribosomal protein L35ae, putative	2	iCn3D view
PF3D7_1142700	Q8IHT8	methyltransferase, putative	1	iCn3D view
PF3D7_1142800	Q8IHT7	ATP synthase-associated protein, putative	3	iCn3D view
PF3D7_1142900	Q8IHT6	conserved Plasmodium protein, unknown function	1	iCn3D view
PF3D7_1143200	Q8IHT4	DnaJ protein, putative	1	iCn3D view
PF3D7_1143300	Q8IHT3	DNA-directed RNA polymerases I and III subunit RPAC1, putative	1	iCn3D view
PF3D7_1143400	Q8IHT2	translation initiation factor eIF-1A, putative	5	iCn3D view
PF3D7_1143600	Q8IHT0	conserved Plasmodium protein, unknown function	1	iCn3D view
PF3D7_1143800	Q8IHS7	oocyst capsule protein Cap93, putative	2	iCn3D view
PF3D7_1143900	Q8IHS6	ATP-dependent (S)-NAD(P)H-hydrate dehydratase, putative	1	iCn3D view
PF3D7_1144000	Q8IHS5	40S ribosomal protein S21	2	iCn3D view
PF3D7_1144200	Q8IHS4	conserved Plasmodium protein, unknown function	1	iCn3D view
PF3D7_1144600	Q8IHS1	transcription initiation factor IIF subunit beta, putative	7	iCn3D view
PF3D7_1144900	Q8IHR8	ras-related protein Rab-6	5	iCn3D view

PF3D7_1145100	Q8IHR6	coatomer subunit gamma, putative	5	iCn3D view
PF3D7_1145200	Q8IHR5	serine/threonine protein kinase, putative	20	iCn3D view
PF3D7_1145300	C6S3G5	cysteine-rich PDZ-binding protein, putative	1	iCn3D view
PF3D7_1145400	Q8IHR4	dynamin-like protein	11	iCn3D view
PF3D7_1145800	Q8IHR0	conserved Plasmodium protein, unknown function	3	iCn3D view
PF3D7_1146000	Q8IHQ8	ribosome assembly protein 4, putative	4	iCn3D view
PF3D7_1146200	Q8IHQ6	conserved Plasmodium protein, unknown function	3	iCn3D view
PF3D7_1146600	Q8IHQ2	oocyst rupture protein 1, putative	38	iCn3D view
PF3D7_1146700	Q8IHQ1	kinesin-X4, putative	1	iCn3D view
PF3D7_1147300	Q8IHP7	conserved Plasmodium protein, unknown function	14	iCn3D view
PF3D7_1147500	Q8IHP6	protein farnesyltransferase subunit beta	1	iCn3D view
PF3D7_1147600	Q8IHP5	conserved Plasmodium protein, unknown function	2	iCn3D view
PF3D7_1147800	A0A144A3C7	membrane associated erythrocyte binding-like protein	1	iCn3D view
PF3D7_1147900	Q8IHP2	peroxisome assembly protein 22, putative	2	iCn3D view
PF3D7_1148000	Q8IHP1	serine/threonine protein kinase, putative	12	iCn3D view
PF3D7_1148900	Q8IHN5	Plasmodium exported protein, unknown function	2	iCn3D view
PF3D7_1149100	Q8IHN2	Plasmodium exported protein, unknown function	4	iCn3D view
PF3D7_1149200	Q8IHN1	ring-infected erythrocyte surface antigen	6	iCn3D view
PF3D7_1149400	Q8IHM9	Plasmodium exported protein, unknown function	1	iCn3D view
PF3D7_1149600	Q8IHM7	DnaJ protein, putative	2	iCn3D view
PF3D7_1200500	Q8I0E0	rifin	1	iCn3D view
PF3D7_1200900	Q8I636	Plasmodium exported protein (PHISTc), unknown function	2	iCn3D view
PF3D7_1201000	Q8I635	Plasmodium exported protein (PHISTb), unknown function	8	iCn3D view
PF3D7_1201100	Q8I634	RESA-like protein with PHIST and DnaJ domains	1	iCn3D view
PF3D7_1201900	Q8I626	conserved protein, unknown function	5	iCn3D view

PF3D7_1202100	Q8I624	SRAP domain-containing protein, putative	6	iCn3D view
PF3D7_1202300	A0A144A1B4	dynein heavy chain, putative	1	iCn3D view
PF3D7_1202500	A0A144A060	trimethylguanosine synthase, putative	2	iCn3D view
PF3D7_1202600	A0A144A0J9	nuclear export mediator factor NEMF, putative	36	iCn3D view
PF3D7_1202700	Q8I618	AATF domain-containing protein, putative	3	iCn3D view
PF3D7_1202900	Q8I616	high mobility group protein B1	5	iCn3D view
PF3D7_1203000	Q8I615	origin recognition complex subunit 1	4	iCn3D view
PF3D7_1203100	Q8I614	conserved Plasmodium protein, unknown function	4	iCn3D view
PF3D7_1203500	Q8I610	threonylcarbamoyl-AMP synthase, putative	2	iCn3D view
PF3D7_1203700	Q8I608	nucleosome assembly protein	10	iCn3D view
PF3D7_1203900	Q8I607	ubiquitin-conjugating enzyme E2	5	iCn3D view
PF3D7_1204100	Q8I605	conserved Plasmodium protein, unknown function	7	iCn3D view
PF3D7_1204200	Q8I604	conserved Plasmodium protein, unknown function	2	iCn3D view
PF3D7_1204300	Q8I603	eukaryotic translation initiation factor 5A	6	iCn3D view
PF3D7_1204900	Q8I5Z6	conserved Plasmodium protein, unknown function	2	iCn3D view
PF3D7_1205000	Q8I5Z5	conserved protein, unknown function	4	iCn3D view
PF3D7_1205100	Q8I5Z4	O-phosphoseryl-tRNA(Sec) selenium transferase, putative	1	iCn3D view
PF3D7_1205200	Q8I5Z3	HAD domain ookinete protein, putative	1	iCn3D view
PF3D7_1205400	Q8I5Z1	kelch domain-containing protein, putative	9	iCn3D view
PF3D7_1205500	Q8I5Z0	zinc finger protein, putative	21	iCn3D view
PF3D7_1205600	Q8I5Y9	tetratricopeptide repeat protein, putative	9	iCn3D view
PF3D7_1205800	Q8I5Y7	high mobility group protein B3, putative	9	iCn3D view
PF3D7_1205900	Q8I5Y6	conserved protein, unknown function	15	iCn3D view
PF3D7_1206200	Q8I5Y3	eukaryotic translation initiation factor 3 subunit C, putative	15	iCn3D view
PF3D7_1206700	Q8I5X8	eukaryotic translation initiation factor 5, putative	16	iCn3D view

PF3D7_1206800	Q8I5X7	conserved Plasmodium protein, unknown function	3	iCn3D view
PF3D7_1206900	A0A143ZZF4	conserved Plasmodium protein, unknown function	5	iCn3D view
PF3D7_1207100	Q8I5X4	pre-rRNA-processing protein ESF1, putative	15	iCn3D view
PF3D7_1207400	Q8I5X1	conserved Plasmodium protein, unknown function	1	iCn3D view
PF3D7_1207600	Q8I0X4	tRNA dimethylallyltransferase, putative	2	iCn3D view
PF3D7_1207800	Q8I5W9	conserved Plasmodium protein, unknown function	2	iCn3D view
PF3D7_1208100	Q8I5W6	conserved Plasmodium protein, unknown function	5	iCn3D view
PF3D7_1208200	A0A5K1K8N7	cysteine repeat modular protein 3	1	iCn3D view
PF3D7_1208400	Q8I5W4	amino acid transporter, putative	20	iCn3D view
PF3D7_1208500	Q8I5W3	conserved Plasmodium protein, unknown function	2	iCn3D view
PF3D7_1208800	Q8I5W0	zinc finger protein, putative	4	iCn3D view
PF3D7_1208900	Q8I5V9	protein phosphatase PPM11, putative	43	iCn3D view
PF3D7_1209000	Q8I5V8	mago-binding protein, putative	3	iCn3D view
PF3D7_1209200	Q8I5V6	U6 snRNA-associated Sm-like protein LSm7, putative	1	iCn3D view
PF3D7_1210100	Q8I5U8	syntaxin, Qa-SNARE family	2	iCn3D view
PF3D7_1211200	Q8I5T8	conserved Plasmodium protein, unknown function	14	iCn3D view
PF3D7_1211300	Q8I5T7	DNA helicase MCM8, putative	3	iCn3D view
PF3D7_1211400	Q7KQK3	heat shock protein DNAJ homologue Pfj4	3	iCn3D view
PF3D7_1211600	Q8I5T5	lysine-specific histone demethylase 1, putative	6	iCn3D view
PF3D7_1211700	Q8I5T4	DNA replication licensing factor MCM5, putative	3	iCn3D view
PF3D7_1211800	Q7KQK2	polyubiquitin	4	iCn3D view
PF3D7_1212100	Q8I5T1	peripheral plastid protein 1, putative	1	iCn3D view
PF3D7_1212300	Q8I5S9	WD repeat-containing protein, putative	3	iCn3D view
PF3D7_1212400	Q8I5S8	tetratricopeptide repeat protein, putative	3	iCn3D view
PF3D7_1212500	Q8I5S7	glycerol-3-phosphate 1-O-acyltransferase	1	iCn3D view

PF3D7_1212900	Q8I5S4	bromodomain protein 2, putative	33	iCn3D view
PF3D7_1213200	Q8I5S3	mediator of RNA polymerase II transcription subunit 18, putative	1	iCn3D view
PF3D7_1213300	Q8I5S2	conserved Plasmodium protein, unknown function	1	iCn3D view
PF3D7_1213400	Q8I5S1	kelch domain-containing protein, putative	1	iCn3D view
PF3D7_1213800	Q8I5R7	proline--tRNA ligase	7	iCn3D view
PF3D7_1213900	A0A144A0U7	W2 domain-containing protein, putative	18	iCn3D view
PF3D7_1214500	A0A144A024	conserved Plasmodium protein, unknown function	2	iCn3D view
PF3D7_1214900	Q8I5Q7	conserved protein, unknown function	2	iCn3D view
PF3D7_1215100	Q8I5Q5	SUN domain-containing protein, putative	2	iCn3D view
PF3D7_1215300	Q8I5Q3	10 kDa chaperonin	1	iCn3D view
PF3D7_1215700	Q8I5Q0	conserved Plasmodium protein, unknown function	3	iCn3D view
PF3D7_1215900	Q8I5P8	serpentine receptor 10	2	iCn3D view
PF3D7_1216000	Q8I5P7	serine--tRNA ligase, putative	1	iCn3D view
PF3D7_1216300	Q8I5P4	signal recognition particle subunit SRP19	1	iCn3D view
PF3D7_1216500	Q8I5P2	male development gene 1	2	iCn3D view
PF3D7_1216900	Q8I5N9	DNA-binding chaperone, putative	6	iCn3D view
PF3D7_1217200	A0A144A1F1	multiple RNA-binding domain-containing protein 1, putative	5	iCn3D view
PF3D7_1217400	Q8I5N4	conserved protein, unknown function	1	iCn3D view
PF3D7_1217500	Q8I5N3	conserved Plasmodium protein, unknown function	11	iCn3D view
PF3D7_1217600	Q8I5N2	anaphase-promoting complex subunit 10, putative	1	iCn3D view
PF3D7_1217900	Q8I5M9	PPPDE peptidase domain-containing protein, putative	5	iCn3D view
PF3D7_1218000	Q8I5M8	thrombospondin-related apical membrane protein	1	iCn3D view
PF3D7_1218200	Q8I5M6	symplekin domain-containing protein, putative	5	iCn3D view
PF3D7_1218400	Q8I5M4	triose or hexose phosphate/phosphate translocator, putative	6	iCn3D view
PF3D7_1218500	Q8I5M3	dynamin-like protein, putative	13	iCn3D view

PF3D7_1218900	Q8I5L8	WD repeat-containing protein, putative	4	iCn3D view
PF3D7_1219100	Q8I5L6	clathrin heavy chain, putative	17	iCn3D view
PF3D7_1219300	Q8I5L5	erythrocyte membrane protein 1, PfEMP1	1	iCn3D view
PF3D7_1220000	Q8I5L2	female development protein FD4, putative	10	iCn3D view
PF3D7_1220100	Q8I5L1	pre-mRNA-processing factor 17, putative	4	iCn3D view
PF3D7_1220400	Q8I5K9	debranching enzyme-associated ribonuclease, putative	3	iCn3D view
PF3D7_1220500	Q8I5K8	ribosome biogenesis protein TSR3, putative	1	iCn3D view
PF3D7_1220700	A0A144A2C9	conserved Plasmodium protein, unknown function	3	iCn3D view
PF3D7_1220900	Q8I5K4	heterochromatin protein 1	18	iCn3D view
PF3D7_1221300	Q8I5K0	EF hand domain-containing protein, putative	7	iCn3D view
PF3D7_1221700	Q8I5J6	FbpA domain protein, putative	1	iCn3D view
PF3D7_1221900	Q8I5J4	conserved Plasmodium membrane protein, unknown function	5	iCn3D view
PF3D7_1222300	Q8I0V4	endoplasmin, putative	16	iCn3D view
PF3D7_1222400	Q8I5J1	AP2 domain transcription factor	4	iCn3D view
PF3D7_1222600	Q8I5I9	AP2 domain transcription factor AP2-G	11	iCn3D view
PF3D7_1222700	Q8I5I8	glideosome-associated protein 45	16	iCn3D view
PF3D7_1223000	Q8I5I5	conserved Plasmodium protein, unknown function	1	iCn3D view
PF3D7_1223100	Q7KQK0	cAMP-dependent protein kinase regulatory subunit	21	iCn3D view
PF3D7_1223200	Q8I5I4	nucleus export protein BRR6, putative	6	iCn3D view
PF3D7_1223400	Q8I5I3	phospholipid-transporting ATPase, putative	23	iCn3D view
PF3D7_1223500	A0A144A125	conserved Plasmodium protein, unknown function	13	iCn3D view
PF3D7_1223600	Q8I5I1	conserved Plasmodium protein, unknown function	1	iCn3D view
PF3D7_1223700	Q8I5I0	vacuolar iron transporter	4	iCn3D view
PF3D7_1224100	Q8I5H6	conserved protein, unknown function	2	iCn3D view
PF3D7_1224200	Q8I5H5	BRO1 domain-containing protein, putative	2	iCn3D view

PF3D7_1224300	Q8I5H4	polyadenylate-binding protein 1, putative	24	iCn3D view
PF3D7_1224400	Q8I5H3	WD repeat-containing protein, putative	10	iCn3D view
PF3D7_1224500	Q8I5H2	histone chaperone ASF1	2	iCn3D view
PF3D7_1225000	Q8I5G7	conserved Plasmodium protein, unknown function	1	iCn3D view
PF3D7_1225200	Q8I5G5	DNA-binding protein, putative	9	iCn3D view
PF3D7_1225600	Q8I5G1	conserved Plasmodium protein, unknown function	15	iCn3D view
PF3D7_1225700	Q8I5G0	VAC14 domain-containing protein, putative	20	iCn3D view
PF3D7_1225800	Q8I5F9	ubiquitin-activating enzyme E1	12	iCn3D view
PF3D7_1226300	Q8I5F4	haloacid dehalogenase-like hydrolase, putative	2	iCn3D view
PF3D7_1226400	Q8I5F3	regulator of MON1-CCZ1 complex, putative	2	iCn3D view
PF3D7_1226600	Q7KQJ9	proliferating cell nuclear antigen 2	7	iCn3D view
PF3D7_1226700	Q8I5F1	U3 small nucleolar RNA-interacting protein 2, putative	3	iCn3D view
PF3D7_1226800	Q8I5F0	ataxin-3, putative	5	iCn3D view
PF3D7_1226900	Q8I5E9	parasitophorous vacuolar protein 2	1	iCn3D view
PF3D7_1227200	Q8I5E6	potassium channel K1	40	iCn3D view
PF3D7_1227300	Q8I5E5	conserved Plasmodium protein, unknown function	2	iCn3D view
PF3D7_1227400	Q8I5E4	conserved Plasmodium protein, unknown function	3	iCn3D view
PF3D7_1227500	Q8I5E3	protein SOC2, putative	5	iCn3D view
PF3D7_1227600	Q8I5E2	kinetochore protein SPC24, putative	40	iCn3D view
PF3D7_1227700	Q8I5E1	protein KIC2	45	iCn3D view
PF3D7_1227800	Q8I5E0	elongator complex protein 3, putative	7	iCn3D view
PF3D7_1228300	Q8I5D5	NIMA related kinase 1	13	iCn3D view
PF3D7_1228600	Q8I5D2	merozoite surface protein 9	6	iCn3D view
PF3D7_1228700	Q8I5D1	conserved Plasmodium protein, unknown function	1	iCn3D view
PF3D7_1228800	Q8I5D0	WD repeat-containing protein, putative	56	iCn3D view

PF3D7_1229000	Q8I5C8	conserved Plasmodium membrane protein, unknown function	3	iCn3D view
PF3D7_1229300	Q8I5C6	PhIL1-interacting candidate PIC1	7	iCn3D view
PF3D7_1229400	Q8I5C5	macrophage migration inhibitory factor	2	iCn3D view
PF3D7_1229500	Q8I5C4	T-complex protein 1 subunit gamma	10	iCn3D view
PF3D7_1229800	A0A144A488	myosin J, putative	11	iCn3D view
PF3D7_1230500	Q8I5B5	WD repeat-containing protein, putative	1	iCn3D view
PF3D7_1230700	Q8I5B3	protein transport protein SEC13	4	iCn3D view
PF3D7_1230800	Q8I5B2	pre-mRNA-splicing regulator WTAP, putative	26	iCn3D view
PF3D7_1230900	A0A144A2N6	serine/threonine protein kinase RIO1, putative	5	iCn3D view
PF3D7_1231000	A0A144A0B4	conserved Plasmodium protein, unknown function	1	iCn3D view
PF3D7_1231100	Q8I5A9	ras-related protein Rab-2	6	iCn3D view
PF3D7_1231200	Q8I5A8	conserved Plasmodium protein, unknown function	2	iCn3D view
PF3D7_1231300	Q8I5A7	conserved Plasmodium protein, unknown function	4	iCn3D view
PF3D7_1231400	Q8I5A6	membrane protein ICM1	15	iCn3D view
PF3D7_1231500	Q8I5A5	mitosis protein dim1, putative	1	iCn3D view
PF3D7_1231600	Q8I5A4	pre-mRNA-splicing factor ATP-dependent RNA helicase PRP2, putative	18	iCn3D view
PF3D7_1231800	Q8I5A3	asparagine-rich protein, putative	6	iCn3D view
PF3D7_1231900	Q8I5A2	WD repeat-containing protein, putative	2	iCn3D view
PF3D7_1232300	Q8I599	cytochrome b5, putative	1	iCn3D view
PF3D7_1232500	A0A144A1B9	CG2-related protein, putative	5	iCn3D view
PF3D7_1233100	Q8I591	conserved protein, unknown function	1	iCn3D view
PF3D7_1233200	A0A144A255	male development protein MD2, putative	3	iCn3D view
PF3D7_1233300	Q8I589	pentatricopeptide repeat domain-containing protein 2, putative	1	iCn3D view
PF3D7_1233600	Q8I586	asparagine and aspartate rich protein 1	35	iCn3D view
PF3D7_1233700	Q8I585	homocysteine S-methyltransferase, putative	1	iCn3D view

PF3D7_1233900	Q8I583	sentrin-specific protease 1	21	iCn3D view
PF3D7_1234100	Q8I581	bromodomain protein 5	52	iCn3D view
PF3D7_1234400	Q8I578	microgamete surface protein MiGS, putative	1	iCn3D view
PF3D7_1234800	Q8I574	splicing factor 3B subunit 3, putative	7	iCn3D view
PF3D7_1234900	Q8I573	CHCH domain-containing protein, putative	2	iCn3D view
PF3D7_1235100	Q8I571	conserved protein, unknown function	3	iCn3D view
PF3D7_1235200	Q8I570	V-type K+-independent H+-translocating inorganic pyrophosphatase	12	iCn3D view
PF3D7_1235500	Q8I567	N6-adenosine-methyltransferase, putative	6	iCn3D view
PF3D7_1235600	Q8I566	serine hydroxymethyltransferase	11	iCn3D view
PF3D7_1235700	Q8I0V2	ATP synthase subunit beta, mitochondrial	1	iCn3D view
PF3D7_1235800	Q8I565	peptidase, putative	1	iCn3D view
PF3D7_1235900	A0A144A2S8	pre-mRNA-splicing factor SYF1, putative	4	iCn3D view
PF3D7_1236000	Q8I563	vesicle transport v-SNARE protein VTI1, putative	8	iCn3D view
PF3D7_1236100	Q8I562	clustered-asparagine-rich protein	5	iCn3D view
PF3D7_1236900	Q8I554	conserved Plasmodium protein, unknown function	1	iCn3D view
PF3D7_1237000	Q8I553	SUMO-activating enzyme subunit 2	6	iCn3D view
PF3D7_1237100	Q8I552	conserved Plasmodium protein, unknown function	1	iCn3D view
PF3D7_1237400	Q8I549	hydrolase, putative	1	iCn3D view
PF3D7_1237500	Q8I548	conserved Plasmodium protein, unknown function	38	iCn3D view
PF3D7_1237600	A0A144A0C0	periodic tryptophan protein 1, putative	1	iCn3D view
PF3D7_1237700	Q8I546	conserved protein, unknown function	2	iCn3D view
PF3D7_1238100	Q8I542	calcyclin binding protein, putative	5	iCn3D view
PF3D7_1238300	Q8I540	pre-mRNA-splicing factor CWC22, putative	8	iCn3D view
PF3D7_1238500	Q8I538	conserved Plasmodium protein, unknown function	33	iCn3D view
PF3D7_1238600	Q8I537	sphingomyelin phosphodiesterase	13	iCn3D view

PF3D7_1238700	A0A144A4I8	BTB/POZ domain-containing protein, putative	5	iCn3D view
PF3D7_1238800	Q8I535	acyl-CoA synthetase	6	iCn3D view
PF3D7_1238900	Q8I534	protein kinase 2	10	iCn3D view
PF3D7_1239000	Q8I533	HD superfamily phosphohydrolase protein, putative	3	iCn3D view
PF3D7_1239200	Q8I531	AP2 domain transcription factor, putative	48	iCn3D view
PF3D7_1239500	Q8I528	DNA gyrase subunit B	1	iCn3D view
PF3D7_1239700	Q8I526	ATP-dependent zinc metalloprotease FTSH 1	2	iCn3D view
PF3D7_1239800	Q8I525	conserved Plasmodium protein, unknown function	76	iCn3D view
PF3D7_1239900	Q8I524	vacuolar protein sorting-associated protein 16, putative	2	iCn3D view
PF3D7_1240000	Q8I523	3-hydroxyisobutyryl-CoA hydrolase, putative	1	iCn3D view
PF3D7_1240300	Q8I521	erythrocyte membrane protein 1, PfEMP1	1	iCn3D view
PF3D7_1240400	Q8I520	erythrocyte membrane protein 1, PfEMP1	1	iCn3D view
PF3D7_1240900	A0A144A1K0	erythrocyte membrane protein 1, PfEMP1	1	iCn3D view
PF3D7_1241200	Q8I517	nucleoporin NUP269, putative	35	iCn3D view
PF3D7_1241400	Q8I515	female development protein FD1, putative	5	iCn3D view
PF3D7_1241500	Q8I514	conserved Plasmodium protein, unknown function	10	iCn3D view
PF3D7_1241700	Q8I512	replication factor C subunit 4, putative	4	iCn3D view
PF3D7_1242200	Q8I507	queuine tRNA-ribosyltransferase, putative	1	iCn3D view
PF3D7_1242300	Q8I506	conserved Plasmodium membrane protein, unknown function	2	iCn3D view
PF3D7_1242400	Q8I505	GAS8-like protein, putative	1	iCn3D view
PF3D7_1242700	Q8I502	40S ribosomal protein S17, putative	4	iCn3D view
PF3D7_1242800	Q8I501	rab specific GDP dissociation inhibitor	1	iCn3D view
PF3D7_1243000	Q8I4Z9	syntaxin-16, putative	4	iCn3D view
PF3D7_1243100	Q8I4Z8	zinc finger protein, putative	3	iCn3D view
PF3D7_1243400	Q8I4Z6	conserved Plasmodium protein, unknown function	10	iCn3D view

PF3D7_1243500	A0A144A0N0	vacuolar-sorting protein SNF7, putative	5	iCn3D view
PF3D7_1243600	Q8I4Z4	translation initiation factor SUI1, putative	1	iCn3D view
PF3D7_1243700	Q8I4Z3	ubiquitin-conjugating enzyme E2, putative	3	iCn3D view
PF3D7_1243900	Q8I4Z1	double C2-like domain-containing protein	21	iCn3D view
PF3D7_1244100	Q8I4Y9	N-alpha-acetyltransferase 15, NatA auxiliary subunit, putative	11	iCn3D view
PF3D7_1244200	Q8I4Y8	RNA polymerase II transcription factor B subunit 2, putative	7	iCn3D view
PF3D7_1244600	Q8I4Y5	ADP-ribosylation factor GTPase-activating protein 1	7	iCn3D view
PF3D7_1245100	Q8I4Y0	kinesin-13, putative	25	iCn3D view
PF3D7_1245500	A0A144A322	conserved Plasmodium protein, unknown function	5	iCn3D view
PF3D7_1245600	Q8I4X5	kinesin-15, putative	8	iCn3D view
PF3D7_1245800	Q8I4X4	epsin-like protein, putative	4	iCn3D view
PF3D7_1246200	Q8I4X0	actin I	30	iCn3D view
PF3D7_1246300	Q8I4W9	protein KIC4	10	iCn3D view
PF3D7_1246400	Q8I4W8	myosin A-tail interacting protein	9	iCn3D view
PF3D7_1246600	Q8I4W6	pre-mRNA-splicing factor CWC26, putative	12	iCn3D view
PF3D7_1246700	Q8I4W5	conserved Plasmodium protein, unknown function	1	iCn3D view
PF3D7_1246800	Q8I4W4	signal recognition particle receptor subunit beta, putative	4	iCn3D view
PF3D7_1246900	Q8I4W3	RAC-beta serine/threonine protein kinase	1	iCn3D view
PF3D7_1247200	Q8I4W0	conserved protein, unknown function	2	iCn3D view
PF3D7_1247400	Q8I4V8	peptidyl-prolyl cis-trans isomerase FKBP35	4	iCn3D view
PF3D7_1247500	Q8I4V7	serine/threonine protein kinase, putative	16	iCn3D view
PF3D7_1247800	Q8I0V1	dipeptidyl aminopeptidase 2	1	iCn3D view
PF3D7_1248000	A0A144A1E9	tRNA-splicing endonuclease, putative	2	iCn3D view
PF3D7_1248200	Q8I4V2	pre-mRNA-splicing factor RBM22, putative	4	iCn3D view
PF3D7_1248400	Q8I4V0	conserved Plasmodium protein, unknown function	5	iCn3D view

PF3D7_1248500	Q8I4U9	bax inhibitor 1, putative	4	iCn3D view
PF3D7_1248900	Q8I4U5	26S protease regulatory subunit 8, putative	3	iCn3D view
PF3D7_1249100	Q8I4U3	THUMP domain-containing protein, putative	13	iCn3D view
PF3D7_1249600	A0A144A0U1	leucine-rich repeat protein	1	iCn3D view
PF3D7_1250200	Q8I4T2	CSC1-like protein, putative	24	iCn3D view
PF3D7_1250300	Q8I4T1	vacuolar protein sorting-associated protein 26, putative	1	iCn3D view
PF3D7_1250500	A0A144A372	AP-3 complex subunit sigma, putative	1	iCn3D view
PF3D7_1250600	Q8I4S8	translation initiation factor eIF-2B subunit beta, putative	5	iCn3D view
PF3D7_1250800	Q8I4S6	DNA repair protein rhp16, putative	7	iCn3D view
PF3D7_1250900	Q8I4S5	conserved protein, unknown function	2	iCn3D view
PF3D7_1251100	Q8I4S3	conserved Plasmodium protein, unknown function	1	iCn3D view
PF3D7_1251200	Q8I4S2	coronin	35	iCn3D view
PF3D7_1251500	A0A144A382	ATP-dependent RNA helicase DRS1, putative	1	iCn3D view
PF3D7_1251600	Q8I4R8	conserved Plasmodium protein, unknown function	9	iCn3D view
PF3D7_1251800	Q8I4R6	conserved Plasmodium protein, unknown function	7	iCn3D view
PF3D7_1252600	Q8I4R0	esterase, putative	1	iCn3D view
PF3D7_1252700	Q8I4Q9	Plasmodium exported protein (PHISTb), unknown function	2	iCn3D view
PF3D7_1252800	Q8I4Q8	Plasmodium exported protein (PHISTb), unknown function	1	iCn3D view
PF3D7_1253100	Q8I4Q5	Plasmodium exported protein (PHISTa), unknown function	4	iCn3D view
PF3D7_1253400	Q8I0X2	acyl-CoA synthetase	1	iCn3D view
PF3D7_1301200	Q8IEU3	GBPH2 protein	1	iCn3D view
PF3D7_1301400	Q8IEJ2	Plasmodium exported protein (hyp12), unknown function	1	iCn3D view
PF3D7_1301600	Q76NM5	erythrocyte binding antigen-140	9	iCn3D view
PF3D7_1301700	Q8IEJ0	CX3CL1-binding protein 2	2	iCn3D view
PF3D7_1302000	Q8IEI6	EMP1-trafficking protein	1	iCn3D view

PF3D7_1302100	Q8IEU2	gamete antigen 27/25	5	iCn3D view
PF3D7_1302300	C0H595	Plasmodium exported protein, unknown function	1	iCn3D view
PF3D7_1302500	Q8IET9	AAA domain-containing protein, putative	7	iCn3D view
PF3D7_1302600	Q8I701	deoxyhypusine hydroxylase	1	iCn3D view
PF3D7_1302700	Q8IET8	ATP-dependent RNA helicase DHR1, putative	9	iCn3D view
PF3D7_1302800	Q8IET7	40S ribosomal protein S7, putative	10	iCn3D view
PF3D7_1303200	Q8IET3	SNARE protein, putative	3	iCn3D view
PF3D7_1303300	Q8IET2	conserved Plasmodium protein, unknown function	1	iCn3D view
PF3D7_1303400	Q8IET1	LisH domain-containing protein, putative	28	iCn3D view
PF3D7_1303700	Q8IES8	tetratricopeptide repeat protein, putative	3	iCn3D view
PF3D7_1303800	Q8IES7	conserved Plasmodium protein, unknown function	34	iCn3D view
PF3D7_1304000	C0H598	condensin complex subunit 2, putative	13	iCn3D view
PF3D7_1304100	Q8IES4	DNA ligase I	10	iCn3D view
PF3D7_1304200	C0H599	CorA-like Mg2+ transporter protein, putative	12	iCn3D view
PF3D7_1304500	Q8IES0	small heat shock protein, putative	10	iCn3D view
PF3D7_1304900	Q8IER7	DNA-directed RNA polymerase II subunit RPB11, putative	3	iCn3D view
PF3D7_1305000	Q8IER6	MCL1 domain-containing protein, putative	27	iCn3D view
PF3D7_1305300	C0H5A0	translational activator GCN1, putative	10	iCn3D view
PF3D7_1305400	Q8IER1	AAR2 protein, putative	1	iCn3D view
PF3D7_1305500	Q8IER0	mitogen-activated protein kinase phosphatase 1, putative	1	iCn3D view
PF3D7_1305600	Q8IEQ9	site-2 protease S2P, putative	2	iCn3D view
PF3D7_1305900	Q8IEQ6	conserved Plasmodium protein, unknown function	13	iCn3D view
PF3D7_1306000	Q8IEQ5	conserved Plasmodium protein, unknown function	3	iCn3D view
PF3D7_1306400	Q8IEQ1	26S protease regulatory subunit 10B, putative	2	iCn3D view
PF3D7_1306700	Q8IEP8	conserved Plasmodium protein, unknown function	3	iCn3D view

PF3D7_1307100	Q8IEP4	U3 small nucleolar RNA-associated protein 6, putative	3	iCn3D view
PF3D7_1307200	Q8IEP3	DnaJ protein, putative	3	iCn3D view
PF3D7_1307500	Q8IEP0	conserved Plasmodium protein, unknown function	3	iCn3D view
PF3D7_1307700	Q8IEN8	TOM1-like protein, putative	23	iCn3D view
PF3D7_1307800	Q8IEN7	centrosomal protein CEP170, putative	19	iCn3D view
PF3D7_1308000	C0H5A5	conserved Plasmodium membrane protein, unknown function	20	iCn3D view
PF3D7_1308100	Q8IEN4	CCAAT-binding transcription factor, putative	1	iCn3D view
PF3D7_1308200	Q8IEN3	carbamoyl phosphate synthetase	24	iCn3D view
PF3D7_1308300	Q8IEN2	40S ribosomal protein S27	2	iCn3D view
PF3D7_1308500	Q8IEN0	conserved Plasmodium protein, unknown function	2	iCn3D view
PF3D7_1308600	C0H5A7	conserved Plasmodium protein, unknown function	1	iCn3D view
PF3D7_1308700	Q8IEM7	conserved Plasmodium protein, unknown function	1	iCn3D view
PF3D7_1308900	Q8IEM5	mRNA-decapping enzyme 2, putative	22	iCn3D view
PF3D7_1309100	Q8IEM3	60S ribosomal protein L24, putative	6	iCn3D view
PF3D7_1309200	Q8IEM2	protein phosphatase PPM6, putative	16	iCn3D view
PF3D7_1309300	Q8IEM1	U4/U6 small nuclear ribonucleoprotein PRP3, putative	3	iCn3D view
PF3D7_1309400	Q8IEM0	HORMA domain protein, putative	1	iCn3D view
PF3D7_1309500	Q8I700	H/ACA ribonucleoprotein complex subunit 1, putative	6	iCn3D view
PF3D7_1309700	Q8IEL8	vacuolar protein sorting-associated protein 18, putative	7	iCn3D view
PF3D7_1310300	Q8IEL2	zinc finger protein, putative	1	iCn3D view
PF3D7_1310700	Q8IEK9	PhIL1-interacting candidate PIC5	7	iCn3D view
PF3D7_1310800	Q8IEK8	tetratricopeptide repeat protein, putative	3	iCn3D view
PF3D7_1311100	Q8IEK6	meiosis-specific nuclear structural protein 1, putative	2	iCn3D view
PF3D7_1311200	Q8IEK5	transducin beta-like protein TBL1, putative	1	iCn3D view
PF3D7_1311400	Q8IEK4	AP-1 complex subunit mu-1	2	iCn3D view

PF3D7_1311500	Q8IEK3	26S protease regulatory subunit 7, putative	3	iCn3D view
PF3D7_1311800	O96935	M1-family alanyl aminopeptidase	8	iCn3D view
PF3D7_1311900	Q76NM6	V-type proton ATPase catalytic subunit A	9	iCn3D view
PF3D7_1312100	Q8IEK0	GYF domain-containing protein, putative	17	iCn3D view
PF3D7_1312600	Q8IEJ6	2-oxoisovalerate dehydrogenase subunit alpha, mitochondrial, putative	1	iCn3D view
PF3D7_1312800	Q8IEJ4	protein AAP2	86	iCn3D view
PF3D7_1313000	Q8IEI4	ubiquitin-like protein Nedd8	1	iCn3D view
PF3D7_1313500	Q8IEH8	TMEM238 domain-containing protein, putative	11	iCn3D view
PF3D7_1313600	Q8IEH7	Clu domain-containing protein, putative	8	iCn3D view
PF3D7_1313700	Q8IEH6	Maf-like protein, putative	3	iCn3D view
PF3D7_1313800	C0H5B3	conserved Plasmodium membrane protein, unknown function	24	iCn3D view
PF3D7_1314200	Q8IEH2	telomerase reverse transcriptase	1	iCn3D view
PF3D7_1314700	Q8IEG8	pinin/SDK/MemA domain-containing protein, putative	26	iCn3D view
PF3D7_1315300	Q8IEG2	conserved protein, unknown function	5	iCn3D view
PF3D7_1315400	A0A5K1K8E3	zinc finger (CCCH type) protein, putative	2	iCn3D view
PF3D7_1315500	Q8IEF9	conserved protein, unknown function	7	iCn3D view
PF3D7_1315700	Q8IEF7	tRNA (adenine(58)-N(1))-methyltransferase catalytic subunit TRM61, putative	1	iCn3D view
PF3D7_1315900	A0A5K1K8Q1	exportin-T, putative	2	iCn3D view
PF3D7_1316500	A0A5K1K8A8	pre-mRNA-processing factor 40, putative	7	iCn3D view
PF3D7_1316600	Q8IEE9	choline-phosphate cytidylyltransferase	26	iCn3D view
PF3D7_1316800	A0A5K1K9E7	protein transport protein SEC20, putative	3	iCn3D view
PF3D7_1316900	C0H5C0	conserved protein, unknown function	7	iCn3D view
PF3D7_1317000	Q8I6Z8	U4/U6.U5 tri-snRNP-associated protein 2, putative	8	iCn3D view
PF3D7_1317100	Q8IEE5	DNA replication licensing factor MCM4	21	iCn3D view
PF3D7_1317200	Q8IEE4	AP2 domain transcription factor AP2-FG, putative	1	iCn3D view

PF3D7_1317400	A0A5K1K928	zinc finger protein, putative	7	iCn3D view
PF3D7_1317600	C0H5C1	conserved Plasmodium protein, unknown function	4	iCn3D view
PF3D7_1317900	Q8IED7	nucleolar complex protein 4, putative	7	iCn3D view
PF3D7_1318000	Q8IED6	conserved protein, unknown function	4	iCn3D view
PF3D7_1318400	Q8IED2	structural maintenance of chromosomes protein 2, putative	1	iCn3D view
PF3D7_1318700	Q8IEC9	protein AAP4	14	iCn3D view
PF3D7_1318800	Q8IEC8	translocation protein SEC63, putative	9	iCn3D view
PF3D7_1319000	Q8IEC6	conserved Plasmodium protein, unknown function	23	iCn3D view
PF3D7_1319100	A0A5K1K970	ubiquitin carboxyl-terminal hydrolase MINDY, putative	14	iCn3D view
PF3D7_1319200	Q8IEC4	tetratricopeptide repeat protein, putative	1	iCn3D view
PF3D7_1319300	Q8IEC3	tRNA (guanine(26)-N(2))-dimethyltransferase, putative	15	iCn3D view
PF3D7_1319600	A0A5K1K8F4	female development protein FD3, putative	2	iCn3D view
PF3D7_1319700	Q8IEB9	protein phosphatase PP2A regulatory subunit A, putative	1	iCn3D view
PF3D7_1319800	Q8IEB8	conserved Plasmodium protein, unknown function	1	iCn3D view
PF3D7_1320500	C0H5C6	SNARE protein, putative	4	iCn3D view
PF3D7_1320600	Q76NM4	ras-related protein Rab-11A	5	iCn3D view
PF3D7_1320700	Q8IEA7	conserved protein, unknown function	22	iCn3D view
PF3D7_1321100	C0H5C8	protein kinase domain-containing protein, putative	11	iCn3D view
PF3D7_1321200	Q8IEA2	zinc finger protein, putative	1	iCn3D view
PF3D7_1321300	Q8IEA1	conserved Plasmodium membrane protein, unknown function	8	iCn3D view
PF3D7_1321400	Q8IEA0	palmitoyltransferase DHHC8, putative	1	iCn3D view
PF3D7_1321500	Q8I6Z7	3',5'-cyclic nucleotide phosphodiesterase beta	36	iCn3D view
PF3D7_1321600	B3FEM7	cGMP-specific 3',5'-cyclic phosphodiesterase gamma, putative	1	iCn3D view
PF3D7_1321700	Q8IE99	splicing factor 1	16	iCn3D view
PF3D7_1321800	Q8IE98	protein transport protein SFT2, putative	1	iCn3D view

PF3D7_1321900	Q8IE97	conserved protein, unknown function	2	iCn3D view
PF3D7_1322100	Q8IE95	histone-lysine N-methyltransferase SET2	5	iCn3D view
PF3D7_1322200	A0A5K1K9E8	protein STU2, putative	28	iCn3D view
PF3D7_1322300	A0A5K1K8R9	BRCT domain-containing protein, putative	4	iCn3D view
PF3D7_1322600	A0A5K1K973	conserved Plasmodium protein, unknown function	2	iCn3D view
PF3D7_1323100	Q8IE85	60S ribosomal protein L6, putative	1	iCn3D view
PF3D7_1323200	Q8IE84	V-type proton ATPase subunit G, putative	1	iCn3D view
PF3D7_1323300	Q8IE83	N-acetyltransferase, GNAT family, putative	5	iCn3D view
PF3D7_1323400	Q8IE82	60S ribosomal protein L23	1	iCn3D view
PF3D7_1323700	Q8IE80	glideosome associated protein with multiple membrane spans 1	3	iCn3D view
PF3D7_1323900	A0A5K1K8R6	protein BCP1, putative	2	iCn3D view
PF3D7_1324000	Q8IE77	conserved Plasmodium protein, unknown function	1	iCn3D view
PF3D7_1324100	Q8IE76	cyclin-like protein, putative	1	iCn3D view
PF3D7_1324300	Q8IE74	conserved Plasmodium membrane protein, unknown function	4	iCn3D view
PF3D7_1324500	Q8IE72	DEAD box helicase, putative	5	iCn3D view
PF3D7_1324600	Q8IE71	conserved Plasmodium protein, unknown function	9	iCn3D view
PF3D7_1324700	C0H5D3	SNARE protein, putative	1	iCn3D view
PF3D7_1324800	Q8IE69	dihydrofolate synthase/folylpolyglutamate synthase	2	iCn3D view
PF3D7_1324900	Q76NM3	L-lactate dehydrogenase	3	iCn3D view
PF3D7_1325100	Q8IE67	phosphoribosylpyrophosphate synthetase	2	iCn3D view
PF3D7_1326300	A0A5K1K940	RNA-binding protein, putative	8	iCn3D view
PF3D7_1326400	A0A5K1K977	translation initiation factor eIF-2B subunit gamma, putative	2	iCn3D view
PF3D7_1326500	A0A5K1K847	conserved Plasmodium protein, unknown function	10	iCn3D view
PF3D7_1326600	A0A5K1K8I1	conserved Plasmodium protein, unknown function	10	iCn3D view
PF3D7_1326700	C0H5E0	ubiquitin-activating enzyme, putative	14	iCn3D view

PF3D7_1326800	C0H5E1	syntaxin, Qa-SNARE family	2	iCn3D view
PF3D7_1326900	C0H5E2	conserved Plasmodium membrane protein, unknown function	3	iCn3D view
PF3D7_1327200	Q8IE34	ribonuclease P protein subunit RPR2, putative	2	iCn3D view
PF3D7_1327400	A0A5K1K8S4	conserved Plasmodium protein, unknown function	1	iCn3D view
PF3D7_1327500	A0A5K1K9F0	conserved protein, unknown function	4	iCn3D view
PF3D7_1327600	Q8IE38	nicotinamide/nicotinic acid mononucleotide adenylyltransferase	3	iCn3D view
PF3D7_1327800	Q8IE40	ribose-phosphate pyrophosphokinase, putative	1	iCn3D view
PF3D7_1328100	Q8I6T3	proteasome subunit beta type-7, putative	1	iCn3D view
PF3D7_1328200	A0A5K1K982	BRCA2 protein, putative	9	iCn3D view
PF3D7_1328300	Q8IE43	conserved protein, unknown function	10	iCn3D view
PF3D7_1328500	A0A5K1K859	alpha/beta-hydrolase, putative	21	iCn3D view
PF3D7_1329000	Q8IE49	DNA-directed RNA polymerase III subunit RPC1, putative	4	iCn3D view
PF3D7_1329100	Q8IE50	myosin F, putative	15	iCn3D view
PF3D7_1329300	Q8IE52	chromatin assembly factor 1 subunit B, putative	9	iCn3D view
PF3D7_1329500	A0A5K1K8J1	conserved protein, unknown function	7	iCn3D view
PF3D7_1329600	A0A5K1K8T4	conserved Plasmodium protein, unknown function	2	iCn3D view
PF3D7_1330000	Q8IE26	PITH domain-containing protein, putative	3	iCn3D view
PF3D7_1330400	Q8IE22	ER lumen protein retaining receptor 1, putative	1	iCn3D view
PF3D7_1330500	Q8IE21	CTLH domain-containing protein, putative	2	iCn3D view
PF3D7_1330800	Q8IE18	RNA-binding protein, putative	9	iCn3D view
PF3D7_1331100	C0H5F0	DNA polymerase theta, putative	2	iCn3D view
PF3D7_1331200	A0A5K1K984	conserved Plasmodium protein, unknown function	3	iCn3D view
PF3D7_1331300	Q8IE14	signal peptidase complex catalytic subunit SEC11	1	iCn3D view
PF3D7_1331500	A0A5K1K8J9	ATF7-int domain-containing protein, putative	2	iCn3D view
PF3D7_1331700	Q8IE10	glutamine--tRNA ligase, putative	6	iCn3D view

PF3D7_1331800	Q8IE09	60S ribosomal protein L23, putative	2	iCn3D view
PF3D7_1332000	Q8IE08	syntaxin, Qa-SNARE family	5	iCn3D view
PF3D7_1332200	A0A5K1K8U2	conserved protein, unknown function	14	iCn3D view
PF3D7_1332400	A0A5K1K8X2	nucleotidyltransferase, putative	8	iCn3D view
PF3D7_1332500	Q8IE03	SAM-dependent RNA methyltransferase, putative	1	iCn3D view
PF3D7_1332600	Q8IE02	DNA-(apurinic or apyrimidinic site) lyase 1	5	iCn3D view
PF3D7_1332800	Q8IE00	eukaryotic translation initiation factor 6, putative	3	iCn3D view
PF3D7_1332900	Q8IDZ9	isoleucine--tRNA ligase, putative	5	iCn3D view
PF3D7_1333100	A0A5K1K8G1	conserved Plasmodium protein, unknown function	1	iCn3D view
PF3D7_1333700	Q8IDZ1	histone H3-like centromeric protein CSE4	1	iCn3D view
PF3D7_1333800	A0A5K1K8U1	Voldacs domain-containing protein, putative	6	iCn3D view
PF3D7_1334000	Q8IDY8	RING zinc finger protein, putative	9	iCn3D view
PF3D7_1334100	A0A5K1K8V1	origin recognition complex subunit 4, putative	14	iCn3D view
PF3D7_1334200	A0A5K1K963	chaperone binding protein, putative	4	iCn3D view
PF3D7_1334300	Q8IDY5	MSP7-like protein	1	iCn3D view
PF3D7_1335000	Q8IDX9	MSP7-like protein	1	iCn3D view
PF3D7_1335100	Q8IDX8	merozoite surface protein 7	4	iCn3D view
PF3D7_1335600	Q8IDX4	WD repeat-containing protein, putative	3	iCn3D view
PF3D7_1335800	Q8IDX2	conserved Plasmodium protein, unknown function	7	iCn3D view
PF3D7_1336000	Q8IDX1	conserved Plasmodium protein, unknown function	1	iCn3D view
PF3D7_1336200	Q8IDW9	conserved Plasmodium protein, unknown function	12	iCn3D view
PF3D7_1336300	Q8IDW8	conserved Plasmodium membrane protein, unknown function	3	iCn3D view
PF3D7_1336400	Q8IDW7	RanBPM and CLTH-like protein, putative	3	iCn3D view
PF3D7_1336800	Q8IDW4	nuclear movement protein, putative	4	iCn3D view
PF3D7_1336900	Q8IDW3	tryptophan--tRNA ligase	2	iCn3D view

PF3D7_1337100	Q8IDW1	protein kinase 6	1	iCn3D view
PF3D7_1337200	Q8IDW0	1-deoxy-D-xylulose 5-phosphate synthase	1	iCn3D view
PF3D7_1337300	A0A5K1K8K9	exoribonuclease, putative	1	iCn3D view
PF3D7_1337400	A0A5K1K8V2	zinc finger protein, putative	3	iCn3D view
PF3D7_1337500	A0A5K1K8Y0	conserved Plasmodium protein, unknown function	9	iCn3D view
PF3D7_1337700	Q8IDV6	phosphoinositide-binding protein PH2	1	iCn3D view
PF3D7_1337800	A0A5K1K8H0	calcium-dependent protein kinase 5	4	iCn3D view
PF3D7_1338000	C0H5F8	LMBR1 domain-containing protein, putative	1	iCn3D view
PF3D7_1338100	A0A5K1K9F3	26S proteasome regulatory subunit RPN3, putative	1	iCn3D view
PF3D7_1338200	A0A5K1K8V8	60S ribosomal protein L6, putative	6	iCn3D view
PF3D7_1338300	A0A5K1K967	elongation factor 1-gamma, putative	7	iCn3D view
PF3D7_1339300	A0A5K1K8I2	conserved protein, unknown function	5	iCn3D view
PF3D7_1339700	A0A5K1K969	conserved Plasmodium protein, unknown function	42	iCn3D view
PF3D7_1339900	A0A5K1K993	ABC transporter B family member 5, putative	2	iCn3D view
PF3D7_1340200	C0H5G1	conserved Plasmodium protein, unknown function	1	iCn3D view
PF3D7_1340300	Q8IDT3	nucleolar complex protein 2, putative	9	iCn3D view
PF3D7_1340500	A0A5K1K8Z6	inner centromere protein, putative	2	iCn3D view
PF3D7_1340600	A0A5K1K8J0	RNA lariat debranching enzyme, putative	8	iCn3D view
PF3D7_1340700	C0H5G2	ras-related protein Rab-11B	8	iCn3D view
PF3D7_1340900	Q8IDS7	sodium-dependent phosphate transporter	8	iCn3D view
PF3D7_1341200	Q8IDS6	60S ribosomal protein L18, putative	5	iCn3D view
PF3D7_1341300	C0H5G3	60S ribosomal protein L18-2, putative	4	iCn3D view
PF3D7_1341500	A0A5K1K8W3	inner membrane complex suture component, putative	3	iCn3D view
PF3D7_1341800	Q8IDS1	inner membrane complex protein 1k, putative	1	iCn3D view
PF3D7_1341900	Q8IDS0	V-type proton ATPase subunit D, putative	5	iCn3D view

PF3D7_1342000	Q8IDR9	40S ribosomal protein S6	6	iCn3D view
PF3D7_1342400	Q8IDR5	casein kinase II beta chain	4	iCn3D view
PF3D7_1342500	Q8IDR4	sporozoite protein essential for cell traversal	1	iCn3D view
PF3D7_1342600	Q8IDR3	myosin A	4	iCn3D view
PF3D7_1342800	Q8IDR1	phosphoenolpyruvate carboxykinase	4	iCn3D view
PF3D7_1342900	C0H5G5	AP2 domain transcription factor AP2-HS	2	iCn3D view
PF3D7_1343000	Q8IDQ9	phosphoethanolamine N-methyltransferase	14	iCn3D view
PF3D7_1343100	Q8IDQ8	conserved Plasmodium protein, unknown function	17	iCn3D view
PF3D7_1343300	A0A5K1K996	CDT1-like protein, putative	23	iCn3D view
PF3D7_1343400	Q8IDQ5	DNA repair protein RAD5, putative	7	iCn3D view
PF3D7_1343600	Q8IDQ3	UDP-N-acetylglucosamine pyrophosphorylase, putative	1	iCn3D view
PF3D7_1343700	Q8IDQ2	kelch protein K13	20	iCn3D view
PF3D7_1343900	C0H5G7	U4/U6 small nuclear ribonucleoprotein PRP4, putative	4	iCn3D view
PF3D7_1344100	C0H5G9	TLD domain-containing protein, putative	10	iCn3D view
PF3D7_1344200	C0H5H0	endoplasmic reticulum chaperone GRP170	1	iCn3D view
PF3D7_1344300	C0H5H1	zinc finger protein, putative	10	iCn3D view
PF3D7_1344500	A0A5K1K8P1	U6 snRNA phosphodiesterase, putative	1	iCn3D view
PF3D7_1344700	A0A5K1K8X4	conserved Plasmodium protein, unknown function	11	iCn3D view
PF3D7_1344800	A0A5K1K910	aspartate carbamoyltransferase	1	iCn3D view
PF3D7_1344900	A0A5K1K8J8	conserved Plasmodium protein, unknown function	8	iCn3D view
PF3D7_1345000	C0H5H2	RING zinc finger protein, putative	2	iCn3D view
PF3D7_1345600	A0A5K1K8Y1	inner membrane complex protein	18	iCn3D view
PF3D7_1345800	A0A5K1K976	conserved Plasmodium protein, unknown function	7	iCn3D view
PF3D7_1345900	A0A5K1K999	kinetochore protein SPC25, putative	4	iCn3D view
PF3D7_1346000	Q8IDN7	dynactin subunit 2, putative	2	iCn3D view

PF3D7_1346100	Q8IDN6	protein transport protein SEC61 subunit alpha	2	iCn3D view
PF3D7_1346300	Q8IDN4	DNA/RNA-binding protein Alba 2	7	iCn3D view
PF3D7_1346500	A0A5K1K8P9	conserved Plasmodium protein, unknown function	8	iCn3D view
PF3D7_1347200	Q8IDM6	nucleoside transporter 1	5	iCn3D view
PF3D7_1347300	A0A5K1K8X9	conserved Plasmodium membrane protein, unknown function	1	iCn3D view
PF3D7_1347500	A0A5K1K8Y8	DNA/RNA-binding protein Alba 4	6	iCn3D view
PF3D7_1347700	Q8IDM2	ethanolamine-phosphate cytidylyltransferase	12	iCn3D view
PF3D7_1348200	Q8IDL7	step II splicing factor, putative	2	iCn3D view
PF3D7_1348300	Q8IDL6	elongation factor Tu, putative	7	iCn3D view
PF3D7_1348400	A0A5K1K8R0	conserved Plasmodium membrane protein, unknown function	2	iCn3D view
PF3D7_1348500	Q8IDL4	TBC domain protein, putative	1	iCn3D view
PF3D7_1348600	A0A5K1K8Z2	conserved Plasmodium protein, unknown function	8	iCn3D view
PF3D7_1348900	A0A5K1K8Y7	conserved Plasmodium protein, unknown function	2	iCn3D view
PF3D7_1349100	Q8IDK8	nucleoside diphosphate hydrolase, putative	1	iCn3D view
PF3D7_1349200	Q8IDK7	glutamate--tRNA ligase	12	iCn3D view
PF3D7_1349300	Q8IDK6	tyrosine kinase-like protein	15	iCn3D view
PF3D7_1349500	A0A5K1K9F8	conserved Plasmodium protein, unknown function	15	iCn3D view
PF3D7_1349600	A0A5K1K8Z5	spindle and kinetochore-associated protein 3, putative	7	iCn3D view
PF3D7_1349800	Q8IDK1	GPN-loop GTPase, putative	3	iCn3D view
PF3D7_1350100	Q8IDJ8	lysine--tRNA ligase	4	iCn3D view
PF3D7_1350700	A0A5K1K930	N6-adenine-specific methylase, putative	1	iCn3D view
PF3D7_1350900	A0A5K1K8Z4	AP2 domain transcription factor AP2-O4, putative	1	iCn3D view
PF3D7_1351000	Q8IDI9	phosphatidylinositol transfer protein, putative	18	iCn3D view
PF3D7_1351400	Q8IDI5	60S ribosomal protein L17, putative	3	iCn3D view
PF3D7_1351700	A0A5K1K988	inner membrane complex protein 1f, putative	45	iCn3D view

PF3D7_1351900	A0A5K1K9A6	conserved protein, unknown function	1	iCn3D view
PF3D7_1352400	A0A5K1K907	nucleoporin NUP176, putative	25	iCn3D view
PF3D7_1352600	C0H5I3	protein kinase, putative	1	iCn3D view
PF3D7_1352700	Q8IDH3	intron-binding protein aquarius, putative	16	iCn3D view
PF3D7_1352800	Q8IDH2	vacuolar fusion protein MON1, putative	13	iCn3D view
PF3D7_1353100	Q8IDG9	Plasmodium exported protein, unknown function	12	iCn3D view
PF3D7_1353200	Q8IDG8	membrane associated histidine-rich protein 2	6	iCn3D view
PF3D7_1353300	Q8IDG7	conserved Plasmodium protein, unknown function	44	iCn3D view
PF3D7_1353800	Q8IDG3	proteasome subunit alpha type-4, putative	2	iCn3D view
PF3D7_1353900	Q8IDG2	proteasome subunit alpha type-7, putative	2	iCn3D view
PF3D7_1354200	Q8IDF9	phosphoinositide phosphatase SAC1	2	iCn3D view
PF3D7_1354300	Q8IDF8	large subunit rRNA methyltransferase, putative	12	iCn3D view
PF3D7_1354500	Q8IDF6	adenylosuccinate synthetase	1	iCn3D view
PF3D7_1354900	Q8IDF2	conserved Plasmodium protein, unknown function	14	iCn3D view
PF3D7_1355100	Q8IDF0	DNA replication licensing factor MCM6	2	iCn3D view
PF3D7_1355600	Q8IDE6	PhIL1 interacting protein PIP1	3	iCn3D view
PF3D7_1355700	Q8IDE5	NLI interacting factor-like phosphatase, putative	4	iCn3D view
PF3D7_1355800	C0H5I5	splicing factor 3B subunit 5, putative	1	iCn3D view
PF3D7_1356000	Q8IDE2	conserved Plasmodium protein, unknown function	1	iCn3D view
PF3D7_1356100	Q8IDE1	conserved Plasmodium protein, unknown function	14	iCn3D view
PF3D7_1356200	Q8IDE0	mitochondrial import inner membrane translocase subunit TIM23, putative	1	iCn3D view
PF3D7_1356300	Q8IDD9	ubiquitin-conjugating enzyme E2, putative	1	iCn3D view
PF3D7_1356400	Q8IDD8	phosphatase 2A regulatory subunit-related protein, putative	3	iCn3D view
PF3D7_1356600	Q8IDD6	regulator of chromosome condensation, putative	4	iCn3D view
PF3D7_1356900	P61075	protein kinase 5	1	iCn3D view

PF3D7_1357000	Q8I0P6	elongation factor 1-alpha	27	iCn3D view
PF3D7_1357100	Q8I0P6	elongation factor 1-alpha	27	iCn3D view
PF3D7_1357400	A0A5K1K8N6	conserved Plasmodium protein, unknown function	25	iCn3D view
PF3D7_1357500	Q8IDD0	DNA helicase, putative	13	iCn3D view
PF3D7_1357700	A0A5K1K903	U3 small nucleolar RNA-associated protein 21, putative	9	iCn3D view
PF3D7_1357800	C0H5I7	T-complex protein 1 subunit delta	3	iCn3D view
PF3D7_1357900	Q8IDC6	pyrroline-5-carboxylate reductase, putative	2	iCn3D view
PF3D7_1358100	A0A5K1K911	Sas10 domain-containing protein, putative	4	iCn3D view
PF3D7_1358400	A0A5K1K9A9	conserved Plasmodium protein, unknown function	4	iCn3D view
PF3D7_1358500	Q8IDC0	zinc finger protein, putative	5	iCn3D view
PF3D7_1358700	Q8IDB8	YOP1-like protein, putative	2	iCn3D view
PF3D7_1358800	Q8IDB0	40S ribosomal protein S15	7	iCn3D view
PF3D7_1358900	C0H5I9	GTP-binding protein, putative	2	iCn3D view
PF3D7_1359000	C0H5J0	conserved Plasmodium protein, unknown function	9	iCn3D view
PF3D7_1359100	Q8IDB3	riboflavin kinase, putative	2	iCn3D view
PF3D7_1359300	Q8IDB6	exosome complex exonuclease RRP44	1	iCn3D view
PF3D7_1359400	Q8IDB7	CUGBP Elav-like family member 1	15	iCn3D view
PF3D7_1359500	C0H5J1	conserved Plasmodium protein, unknown function	3	iCn3D view
PF3D7_1359700	C0H5J3	conserved Plasmodium protein, unknown function	12	iCn3D view
PF3D7_1359900	A0A5K1K916	conserved protein, unknown function	47	iCn3D view
PF3D7_1360200	Q8IDA3	ER membrane protein complex subunit 3, putative	1	iCn3D view
PF3D7_1360400	A0A5K1K912	conserved Plasmodium protein, unknown function	5	iCn3D view
PF3D7_1360500	Q8IDA0	guanylyl cyclase beta	5	iCn3D view
PF3D7_1360700	A0A5K1K9G1	E3 SUMO-protein ligase PIAS, putative	19	iCn3D view
PF3D7_1360800	Q76NL8	falcilysin	2	iCn3D view

PF3D7_1360900	C0H5J5	RNA-binding protein, putative	13	iCn3D view
PF3D7_1361100	C0H5J6	protein transport protein Sec24A	12	iCn3D view
PF3D7_1361200	Q8ID94	conserved Plasmodium protein, unknown function	13	iCn3D view
PF3D7_1361500	C0H5J8	PH domain-containing protein, putative	1	iCn3D view
PF3D7_1361800	A0A5K1K8J3	glideosome-associated connector	20	iCn3D view
PF3D7_1361900	P61074	proliferating cell nuclear antigen 1	1	iCn3D view
PF3D7_1362200	Q8ID85	RuvB-like helicase 3	5	iCn3D view
PF3D7_1362400	A0A5K1K8Q4	calpain	9	iCn3D view
PF3D7_1362700	C0H5K2	conserved Plasmodium protein, unknown function	22	iCn3D view
PF3D7_1362800	C0H5K3	conserved Plasmodium protein, unknown function	7	iCn3D view
PF3D7_1363000	A0A5K1K998	conserved Plasmodium protein, unknown function	1	iCn3D view
PF3D7_1363100	A0A5K1K9B5	conserved Plasmodium protein, unknown function	2	iCn3D view
PF3D7_1363400	A0A5K1K8W0	polyubiquitin binding protein, putative	4	iCn3D view
PF3D7_1363500	A0A5K1K929	CCR4 domain-containing protein 2, putative	3	iCn3D view
PF3D7_1364000	A0A5K1K9G3	conserved protein, unknown function	5	iCn3D view
PF3D7_1364200	A0A5K1K9A0	nucleoporin NUP205, putative	17	iCn3D view
PF3D7_1364300	A0A5K1K9B8	pre-mRNA-splicing factor ATP-dependent RNA helicase PRP16	5	iCn3D view
PF3D7_1364500	Q8ID62	exosome complex component RRP45, putative	2	iCn3D view
PF3D7_1364800	Q8ID59	DNA-directed RNA polymerases I, II, and III subunit RPABC1, putative	1	iCn3D view
PF3D7_1365000	A0A5K1K8X0	conserved Plasmodium protein, unknown function	8	iCn3D view
PF3D7_1365700	Q8ID52	SNARE associated Golgi protein, putative	8	iCn3D view
PF3D7_1365900	Q8ID50	ubiquitin-60S ribosomal protein L40	4	iCn3D view
PF3D7_1366100	C0H5K7	DIP13 homolog, putative	3	iCn3D view
PF3D7_1366400	A0A5K1K9G4	rhoptry protein RHOP148	13	iCn3D view
PF3D7_1366500	Q8ID43	nucleoside diphosphate kinase	1	iCn3D view

PF3D7_1366600	Q8ID42	signal recognition particle receptor subunit alpha, putative	9	iCn3D view
PF3D7_1367100	Q8ID37	U1 small nuclear ribonucleoprotein 70 kDa homolog, putative	13	iCn3D view
PF3D7_1367200	C0H5L0	CLAMP domain-containing protein, putative	1	iCn3D view
PF3D7_1367400	Q8ID34	conserved Plasmodium protein, unknown function	5	iCn3D view
PF3D7_1367700	Q8ID31	alanine--tRNA ligase	4	iCn3D view
PF3D7_1368100	Q8ID28	26S proteasome regulatory subunit RPN11, putative	5	iCn3D view
PF3D7_1368300	C0H5L3	non-structural maintenance of chromosomes element 1, putative	1	iCn3D view
PF3D7_1368400	Q8ID26	ribosomal protein L1, putative	1	iCn3D view
PF3D7_1368600	Q8ID24	mitochondrial import inner membrane translocase subunit TIM9, putative	2	iCn3D view
PF3D7_1368800	Q8ID22	DNA repair endonuclease XPF, putative	11	iCn3D view
PF3D7_1368900	A0A5K1K9A4	conserved protein, unknown function	1	iCn3D view
PF3D7_1369000	C0H5L4	glycosylphosphatidylinositol anchor attachment 1 protein, putative	2	iCn3D view
PF3D7_1369100	A0A5K1K9B9	conserved Plasmodium protein, unknown function	3	iCn3D view
PF3D7_1369200	A0A5K1K8M3	conserved Plasmodium protein, unknown function	4	iCn3D view
PF3D7_1369300	Q8ID17	conserved Plasmodium protein, unknown function	1	iCn3D view
PF3D7_1369500	A0A5K1K8X7	nuclear cap-binding protein subunit 1, putative	8	iCn3D view
PF3D7_1369700	C0H5L8	U2 small nuclear ribonucleoprotein A', putative	5	iCn3D view
PF3D7_1370300	C0H5L9	membrane associated histidine-rich protein 1	2	iCn3D view
PF3D7_1371800	A0A146M1M7	Plasmodium exported protein, unknown function	5	iCn3D view
PF3D7_1371900	C0H5M6	Plasmodium exported protein, unknown function	1	iCn3D view
PF3D7_1372000	C0H5M7	Plasmodium exported protein (PHISTa), unknown function	1	iCn3D view
PF3D7_1372100	C0H5M8	Plasmodium exported protein (PHISTb), unknown function	3	iCn3D view
PF3D7_1401000	Q8IM78	GBPH protein	1	iCn3D view
PF3D7_1401100	Q8IM77	DnaJ protein, putative	2	iCn3D view
PF3D7_1401300	Q8IM75	epoxide hydrolase 2	1	iCn3D view

PF3D7_1401400	Q7KQM5	early transcribed membrane protein 14.1	1	iCn3D view
PF3D7_1401500	Q8IM74	esterase, putative	1	iCn3D view
PF3D7_1401600	Q8IM73	Plasmodium exported protein (PHISTb), unknown function	7	iCn3D view
PF3D7_1401800	Q8IM71	choline kinase	1	iCn3D view
PF3D7_1402100	Q8IM68	pseudouridine synthase, putative	1	iCn3D view
PF3D7_1402200	Q8IM67	cytochrome c oxidase subunit ApiCOX19, putative	1	iCn3D view
PF3D7_1402300	Q8IM66	26S proteasome regulatory subunit RPN6	3	iCn3D view
PF3D7_1402400	Q8IM65	zinc finger protein, putative	1	iCn3D view
PF3D7_1402700	A0A144A0W9	U2 snRNP-associated SURP motif-containing protein, putative	4	iCn3D view
PF3D7_1402800	Q8IM62	conserved Plasmodium protein, unknown function	17	iCn3D view
PF3D7_1402900	C6S3H1	conserved Plasmodium protein, unknown function	1	iCn3D view
PF3D7_1403000	Q8IM61	radial spoke protein 3, putative	1	iCn3D view
PF3D7_1403200	C6S3H3	EGF domain-containing protein, putative	2	iCn3D view
PF3D7_1403600	Q8IM58	selenoprotein 1	1	iCn3D view
PF3D7_1403700	Q8IM57	translocation associated membrane protein, putative	1	iCn3D view
PF3D7_1403800	Q8IM56	nuclear formin-like protein MISFIT, putative	4	iCn3D view
PF3D7_1404200	Q8IM52	conserved Plasmodium protein, unknown function	6	iCn3D view
PF3D7_1404800	Q8IM46	conserved Plasmodium protein, unknown function	3	iCn3D view
PF3D7_1405000	Q8IM44	conserved Plasmodium protein, unknown function	9	iCn3D view
PF3D7_1405200	Q8IM42	trafficking protein particle complex subunit 1, putative	1	iCn3D view
PF3D7_1405300	Q8IM41	conserved Plasmodium protein, unknown function	1	iCn3D view
PF3D7_1405400	Q8IM40	DNA mismatch repair protein, putative	1	iCn3D view
PF3D7_1405500	Q8IM39	COBW domain-containing protein 1, putative	1	iCn3D view
PF3D7_1405600	Q8IM38	ribonucleoside-diphosphate reductase small chain, putative	1	iCn3D view
PF3D7_1405700	Q8IM37	RING zinc finger protein, putative	2	iCn3D view

PF3D7_1405800	Q8IM36	ribosome biogenesis protein BOP1, putative	3	iCn3D view
PF3D7_1405900	A0A144A153	RNA-binding protein, putative	2	iCn3D view
PF3D7_1406100	Q8IM33	tubby domain-containing protein, putative	5	iCn3D view
PF3D7_1406200	Q8IM32	transcription elongation factor SPT6, putative	43	iCn3D view
PF3D7_1406500	Q8IM29	WD repeat-containing protein 65, putative	3	iCn3D view
PF3D7_1406700	Q8IM27	vacuolar protein sorting-associated protein 29	2	iCn3D view
PF3D7_1406800	Q8IM26	glideosome associated protein with multiple membrane spans 3	2	iCn3D view
PF3D7_1407100	Q8IM23	rRNA 2'-O-methyltransferase fibrillarin, putative	8	iCn3D view
PF3D7_1407200	Q8IM22	conserved Plasmodium protein, unknown function	5	iCn3D view
PF3D7_1407300	Q8IM21	pre-mRNA-splicing factor 38B, putative	11	iCn3D view
PF3D7_1407500	Q8IM19	multifunctional methyltransferase subunit TRM112, putative	1	iCn3D view
PF3D7_1407800	Q8IM16	plasmepsin IV	7	iCn3D view
PF3D7_1407900	Q7KQM4	plasmepsin I	5	iCn3D view
PF3D7_1408000	Q8I6V3	plasmepsin II	4	iCn3D view
PF3D7_1408100	Q8IM15	plasmepsin III	3	iCn3D view
PF3D7_1408400	Q8IM12	FANCJ-like helicase, putative	4	iCn3D view
PF3D7_1408500	Q8IM11	conserved Plasmodium protein, unknown function	2	iCn3D view
PF3D7_1408600	Q8IM10	40S ribosomal protein S8e, putative	5	iCn3D view
PF3D7_1408700	Q8IM09	conserved protein, unknown function	42	iCn3D view
PF3D7_1408800	Q8IM08	conserved Plasmodium protein, unknown function	1	iCn3D view
PF3D7_1409000	Q8IM06	WD repeat-containing protein, putative	4	iCn3D view
PF3D7_1409200	Q8IM04	conserved Plasmodium protein, unknown function	1	iCn3D view
PF3D7_1409300	Q8IM03	DNA damage-inducible protein 1	3	iCn3D view
PF3D7_1409400	Q8IM01	conserved protein, unknown function	6	iCn3D view
PF3D7_1409500	Q8IM00	conserved Plasmodium protein, unknown function	2	iCn3D view

PF3D7_1409600	Q8ILZ9	histidine phosphatase, putative	10	iCn3D view
PF3D7_1409800	Q8ILZ7	CUGBP Elav-like family member 2, putative	5	iCn3D view
PF3D7_1409900	Q8ILZ6	cytidine diphosphate-diacylglycerol synthase	10	iCn3D view
PF3D7_1410100	Q8ILZ4	alpha/beta hydrolase, putative	1	iCn3D view
PF3D7_1410200	Q8ILZ3	CTP synthase	7	iCn3D view
PF3D7_1410300	Q8ILZ2	WD repeat-containing protein, putative	15	iCn3D view
PF3D7_1410400	Q8ILZ1	rhoptry-associated protein 1	28	iCn3D view
PF3D7_1410600	Q8ILY9	eukaryotic translation initiation factor 2 subunit gamma, putative	6	iCn3D view
PF3D7_1410900	Q8ILY6	ribosome maturation protein SBDS, putative	8	iCn3D view
PF3D7_1411100	Q8ILY4	conserved Plasmodium membrane protein, unknown function	1	iCn3D view
PF3D7_1411300	Q8ILY2	DnaJ protein, putative	9	iCn3D view
PF3D7_1411500	Q8ILY0	conserved Plasmodium protein, unknown function	6	iCn3D view
PF3D7_1412100	Q8ILX3	mini-chromosome maintenance complex-binding protein	6	iCn3D view
PF3D7_1412200	Q8ILX2	MORN repeat protein, putative	1	iCn3D view
PF3D7_1412300	Q8ILX1	nuclear transport factor 2, putative	2	iCn3D view
PF3D7_1412400	Q8ILX0	conserved Plasmodium protein, unknown function	12	iCn3D view
PF3D7_1412500	Q8ILW9	actin II	6	iCn3D view
PF3D7_1412600	Q8ILW8	deoxyhypusine synthase	4	iCn3D view
PF3D7_1412700	Q8ILW7	AAA family ATPase, putative	2	iCn3D view
PF3D7_1412800	Q8ILW6	glycylpeptide N-tetradecanoyltransferase	3	iCn3D view
PF3D7_1413000	Q8ILW4	conserved Plasmodium protein, unknown function	2	iCn3D view
PF3D7_1413200	Q8ILW2	conserved Plasmodium protein, unknown function	1	iCn3D view
PF3D7_1413600	A0A144A1K5	conserved Plasmodium protein, unknown function	1	iCn3D view
PF3D7_1413700	Q8ILV8	DET1 domain-containing protein, putative	22	iCn3D view
PF3D7_1413900	Q8ILV6	DnaJ protein, putative	2	iCn3D view

PF3D7_1414000	Q8ILV5	26S proteasome regulatory subunit RPN13, putative	2	iCn3D view
PF3D7_1414100	Q8ILV4	zinc finger protein, putative	1	iCn3D view
PF3D7_1414300	Q8ILV2	60S ribosomal protein L10, putative	10	iCn3D view
PF3D7_1414400	Q8ILV1	serine/threonine protein phosphatase PP1	2	iCn3D view
PF3D7_1414600	Q8I720	mRNA-capping enzyme subunit alpha	4	iCn3D view
PF3D7_1414800	Q8ILU8	small nuclear ribonucleoprotein-associated protein B, putative	1	iCn3D view
PF3D7_1415000	Q8ILU6	uracil-DNA glycosylase	5	iCn3D view
PF3D7_1415100	Q8ILU5	conserved protein, unknown function	1	iCn3D view
PF3D7_1415300	Q8ILU3	RNA-binding protein Nova-1, putative	3	iCn3D view
PF3D7_1415400	Q8ILU2	Btz domain-containing protein, putative	35	iCn3D view
PF3D7_1415500	A0A144A1F5	conserved Plasmodium membrane protein, unknown function	1	iCn3D view
PF3D7_1415800	Q8ILT8	ribosomal RNA small subunit methyltransferase A1	2	iCn3D view
PF3D7_1415900	Q8ILT7	zinc finger protein, putative	2	iCn3D view
PF3D7_1416100	Q8ILT5	protein SEY1, putative	17	iCn3D view
PF3D7_1416400	Q8ILT2	conserved protein, unknown function	3	iCn3D view
PF3D7_1416600	Q8ILS9	conserved Plasmodium protein, unknown function	5	iCn3D view
PF3D7_1416900	Q8ILS7	prefoldin subunit 2, putative	5	iCn3D view
PF3D7_1417100	Q8ILS5	conserved Plasmodium protein, unknown function	4	iCn3D view
PF3D7_1417300	Q8ILS3	cysteine protease ATG4, putative	10	iCn3D view
PF3D7_1417500	Q8ILS0	H/ACA ribonucleoprotein complex subunit 4, putative	4	iCn3D view
PF3D7_1417600	Q8ILR9	ookinete surface and oocyst capsule protein OSCP, putative	29	iCn3D view
PF3D7_1417700	Q8ILR8	conserved protein, unknown function	7	iCn3D view
PF3D7_1417800	Q8ILR7	DNA replication licensing factor MCM2	8	iCn3D view
PF3D7_1417900	C6S3H7	ATP synthase-associated protein, putative	3	iCn3D view
PF3D7_1418000	Q8ILR6	ubiquitin fusion degradation protein 1, putative	2	iCn3D view

PF3D7_1418100	Q8ILR5	liver specific protein 1, putative	2	iCn3D view
PF3D7_1418200	Q8ILR4	conserved Plasmodium protein, unknown function	3	iCn3D view
PF3D7_1418900	C6S3H8	ATP-dependent RNA helicase DBP4, putative	5	iCn3D view
PF3D7_1419000	Q8ILR0	conserved Plasmodium protein, unknown function	31	iCn3D view
PF3D7_1419100	Q8ILQ9	ATP-dependent RNA helicase DDX55	2	iCn3D view
PF3D7_1419300	Q8ILQ7	glutathione S-transferase	1	iCn3D view
PF3D7_1419600	Q8ILQ4	conserved Plasmodium protein, unknown function	8	iCn3D view
PF3D7_1419700	Q8ILQ3	eukaryotic translation initiation factor 3 subunit H, putative	4	iCn3D view
PF3D7_1419800	O15770	glutathione reductase	1	iCn3D view
PF3D7_1419900	Q8ILQ1	YTH domain-containing protein 1, putative	1	iCn3D view
PF3D7_1420200	Q8ILP8	myosin-specific chaperone UNC, putative	1	iCn3D view
PF3D7_1420400	Q8ILP6	glycine--tRNA ligase	2	iCn3D view
PF3D7_1420700	Q8ILP3	surface protein P113	12	iCn3D view
PF3D7_1421000	Q8ILP0	DIX domain-containing protein, putative	4	iCn3D view
PF3D7_1421100	Q8ILN9	conserved Plasmodium protein, unknown function	3	iCn3D view
PF3D7_1421200	Q8ILN8	40S ribosomal protein S25	3	iCn3D view
PF3D7_1421300	Q8ILN7	conserved Plasmodium protein, unknown function	2	iCn3D view
PF3D7_1421600	Q8ILN4	conserved Plasmodium protein, unknown function	1	iCn3D view
PF3D7_1421800	Q8ILN3	conserved Plasmodium protein, unknown function	1	iCn3D view
PF3D7_1422200	Q8ILN0	conserved Plasmodium protein, unknown function	1	iCn3D view
PF3D7_1422300	C6S3I0	DnaJ protein, putative	2	iCn3D view
PF3D7_1422400	Q8ILM9	nucleolar RNA-associated protein, putative	2	iCn3D view
PF3D7_1422500	Q8ILM8	ERAD-associated E3 ubiquitin-protein ligase HRD1	1	iCn3D view
PF3D7_1422600	Q8ILM7	conserved Plasmodium protein, unknown function	4	iCn3D view
PF3D7_1422800	Q8ILM5	actin-related protein ARP4	7	iCn3D view

PF3D7_1423200	Q8ILM0	peptidyl-prolyl cis-trans isomerase	3	iCn3D view
PF3D7_1423300	Q8ILL9	serine/threonine protein phosphatase 7	6	iCn3D view
PF3D7_1423400	Q8ILL8	conserved Plasmodium membrane protein, unknown function	2	iCn3D view
PF3D7_1423500	Q8ILL7	conserved Plasmodium protein, unknown function	3	iCn3D view
PF3D7_1423700	Q8ILL5	conserved Plasmodium protein, unknown function	30	iCn3D view
PF3D7_1423800	Q8ILL4	vacuolar protein sorting-associated protein 3, putative	3	iCn3D view
PF3D7_1424100	Q8ILL3	60S ribosomal protein L5, putative	4	iCn3D view
PF3D7_1424400	Q8ILL2	60S ribosomal protein L7-3, putative	3	iCn3D view
PF3D7_1425300	A0A144A3H3	conserved Plasmodium protein, unknown function	6	iCn3D view
PF3D7_1425400	Q8ILK9	DEAD/DEAH box helicase, putative	9	iCn3D view
PF3D7_1425500	Q8ILK8	conserved Plasmodium protein, unknown function	1	iCn3D view
PF3D7_1425600	Q8ILK7	zinc finger protein, putative	1	iCn3D view
PF3D7_1425700	Q8ILK6	vacuolar ER assembly factor VMA12, putative	2	iCn3D view
PF3D7_1425800	Q8ILK5	conserved Plasmodium protein, unknown function	16	iCn3D view
PF3D7_1425900	Q8ILK4	protein HGH1, putative	2	iCn3D view
PF3D7_1426000	Q8ILK3	60S ribosomal protein L21	3	iCn3D view
PF3D7_1426100	Q8ILK2	transcription factor BTF3, putative	6	iCn3D view
PF3D7_1426200	Q8ILK1	protein arginine N-methyltransferase 1	2	iCn3D view
PF3D7_1426500	Q8ILJ9	ABC transporter G family member 2	7	iCn3D view
PF3D7_1426600	Q8ILJ8	conserved Plasmodium protein, unknown function	3	iCn3D view
PF3D7_1426700	Q8ILJ7	phosphoenolpyruvate carboxylase	8	iCn3D view
PF3D7_1427300	Q8ILJ1	conserved Plasmodium protein, unknown function	31	iCn3D view
PF3D7_1427400	Q8ILJ0	conserved Plasmodium membrane protein, unknown function	2	iCn3D view
PF3D7_1427500	Q8ILI9	DNA mismatch repair protein MSH2, putative	2	iCn3D view
PF3D7_1427600	Q8ILI8	CorA-like Mg2+ transporter protein, putative	7	iCn3D view

PF3D7_1427900	Q8ILI6	leucine-rich repeat protein	10	iCn3D view
PF3D7_1428100	Q8ILI4	WW domain-binding protein 11, putative	5	iCn3D view
PF3D7_1428300	Q8ILI2	proliferation-associated protein 2g4, putative	5	iCn3D view
PF3D7_1428500	Q8ILH9	protein kinase, putative	5	iCn3D view
PF3D7_1428700	Q8ILH7	heme/steroid binding domain containing protein, putative	2	iCn3D view
PF3D7_1428800	Q8ILH6	transcription initiation TFIID-like, putative	6	iCn3D view
PF3D7_1428900	Q8ILH5	conserved Plasmodium protein, unknown function	10	iCn3D view
PF3D7_1429000	Q8ILH4	protein archease, putative	2	iCn3D view
PF3D7_1429200	Q8ILH2	AP2 domain transcription factor AP2-O3, putative	1	iCn3D view
PF3D7_1429500	Q8ILG9	diphthamide biosynthesis protein 2, putative	1	iCn3D view
PF3D7_1429600	Q8ILG8	conserved Plasmodium protein, unknown function	4	iCn3D view
PF3D7_1429800	Q8ILG6	coatomer subunit beta, putative	16	iCn3D view
PF3D7_1429900	Q8ILG5	ADP-dependent DNA helicase RecQ	12	iCn3D view
PF3D7_1430000	Q8ILG4	conserved protein, unknown function	1	iCn3D view
PF3D7_1430400	Q8ILG0	autophagy protein 5, putative	2	iCn3D view
PF3D7_1430600	Q8ILF8	endonuclease/exonuclease/phosphatase family protein, putative	2	iCn3D view
PF3D7_1430800	Q8ILF6	PhIL1 interacting protein PIP3	4	iCn3D view
PF3D7_1431100	Q8ILF4	PhIL1 interacting protein PIP2	10	iCn3D view
PF3D7_1431200	Q8ILF3	OST-HTH associated domain protein, putative	1	iCn3D view
PF3D7_1431300	Q8ILF2	large subunit GTPase 1, putative	5	iCn3D view
PF3D7_1431400	Q8ILF1	surface-related antigen SRA	1	iCn3D view
PF3D7_1431700	Q8ILE8	60S ribosomal protein L14, putative	3	iCn3D view
PF3D7_1432100	Q8ILE3	voltage-dependent anion-selective channel protein, putative	1	iCn3D view
PF3D7_1432500	Q8ILD7	RNA methyltransferase, putative	1	iCn3D view
PF3D7_1432600	Q8ILD6	DNA-binding protein, putative	1	iCn3D view

PF3D7_1432700	A0A144A3E6	protein-L-isoaspartate(D-aspartate) O-methyltransferase, putative	3	iCn3D view
PF3D7_1432800	Q8ILD4	HP12 protein homolog, putative	2	iCn3D view
PF3D7_1432900	Q8ILD3	SF-assemblin, putative	3	iCn3D view
PF3D7_1433100	Q8ILD2	HID1 domain-containing protein, putative	1	iCn3D view
PF3D7_1433200	Q8ILD1	conserved Plasmodium protein, unknown function	2	iCn3D view
PF3D7_1433300	Q8ILD0	histone-binding protein RBBP7, putative	2	iCn3D view
PF3D7_1433500	Q8ILC8	DNA topoisomerase 2	26	iCn3D view
PF3D7_1433800	Q8ILC5	conserved Plasmodium protein, unknown function	4	iCn3D view
PF3D7_1434000	Q8ILC3	CCR4-associated factor 16, putative	1	iCn3D view
PF3D7_1434200	P62203	calmodulin	7	iCn3D view
PF3D7_1434300	Q8ILC1	Hsp70/Hsp90 organizing protein	3	iCn3D view
PF3D7_1434400	Q8ILC0	conserved protein, unknown function	1	iCn3D view
PF3D7_1434500	Q8ILB9	dynein-related AAA-type ATPase, putative	11	iCn3D view
PF3D7_1434600	Q8ILB8	methionine aminopeptidase 2	2	iCn3D view
PF3D7_1434700	Q8ILB7	mitochondrial import inner membrane translocase subunit TIM17, putative	1	iCn3D view
PF3D7_1435100	Q8ILB3	ribonuclease H2 subunit B, putative	1	iCn3D view
PF3D7_1435300	Q8ILB1	glutamate synthase [NADH], putative	9	iCn3D view
PF3D7_1435400	Q8ILB0	ER membrane protein complex subunit 4, putative	1	iCn3D view
PF3D7_1435500	Q8ILA9	clathrin light chain, putative	18	iCn3D view
PF3D7_1435600	Q8ILA8	conserved Plasmodium protein, unknown function	56	iCn3D view
PF3D7_1435700	Q8ILA7	ataxin-2 like protein, putative	2	iCn3D view
PF3D7_1436100	Q8ILA3	conserved Plasmodium membrane protein, unknown function	9	iCn3D view
PF3D7_1436300	Q8ILA1	translocon component PTEX150	16	iCn3D view
PF3D7_1436600	Q8I719	cGMP-dependent protein kinase	9	iCn3D view
PF3D7_1437000	Q8IL96	N-acetyltransferase, GNAT family	15	iCn3D view

PF3D7_1437200	Q8IL94	ribonucleoside-diphosphate reductase large subunit, putative	7	iCn3D view
PF3D7_1437300	Q8IL93	conserved Plasmodium protein, unknown function	2	iCn3D view
PF3D7_1437400	Q8IL92	pantothenate kinase 2	10	iCn3D view
PF3D7_1437500	Q8IL91	YrhK domain-containing protein, putative	12	iCn3D view
PF3D7_1437800	Q7KQM2	trafficking protein particle complex subunit 5, putative	1	iCn3D view
PF3D7_1437900	Q8IL88	HSP40, subfamily A	6	iCn3D view
PF3D7_1438000	Q8IL87	eukaryotic translation initiation factor eIF2A, putative	4	iCn3D view
PF3D7_1438100	Q8IL86	translocation protein SEC62, putative	5	iCn3D view
PF3D7_1438400	Q8IL84	metacaspase-2	53	iCn3D view
PF3D7_1438500	Q8IL83	cleavage and polyadenylation specificity factor subunit 3, putative	1	iCn3D view
PF3D7_1438700	Q7KQM1	DNA primase small subunit	1	iCn3D view
PF3D7_1438900	Q8IL80	thioredoxin peroxidase 1	2	iCn3D view
PF3D7_1439000	Q8IL79	copper transporter	1	iCn3D view
PF3D7_1439500	Q8IL74	oocyst rupture protein 2, putative	13	iCn3D view
PF3D7_1439800	Q8IL71	vesicle-associated membrane protein, putative	8	iCn3D view
PF3D7_1439900	Q7KQM0	triosephosphate isomerase	3	iCn3D view
PF3D7_1440000	Q8IL70	conserved Plasmodium protein, unknown function	33	iCn3D view
PF3D7_1440100	Q8IL69	cohesin complex subunit, putative	31	iCn3D view
PF3D7_1440400	Q8IL66	zinc finger protein, putative	8	iCn3D view
PF3D7_1440600	A0A144A207	protein SOC3, putative	1	iCn3D view
PF3D7_1440700	Q8IL63	AP-3 complex subunit mu, putative	1	iCn3D view
PF3D7_1441100	Q8IL59	conserved Plasmodium protein, unknown function	12	iCn3D view
PF3D7_1441200	Q8IL58	60S ribosomal protein L1, putative	3	iCn3D view
PF3D7_1441400	Q8IL56	FACT complex subunit SSRP1, putative	9	iCn3D view
PF3D7_1441500	Q8IL55	conserved Plasmodium protein, unknown function	2	iCn3D view

PF3D7_1441600	Q8IL54	acid cluster protein 33 homologue, putative	6	iCn3D view
PF3D7_1442300	Q8IL48	tRNA import protein tRIP	7	iCn3D view
PF3D7_1442400	Q8IL47	protein KIC9	46	iCn3D view
PF3D7_1442600	Q8IL45	TRAP-like protein	1	iCn3D view
PF3D7_1442700	Q8IL44	conserved Plasmodium protein, unknown function	12	iCn3D view
PF3D7_1442900	Q8IL42	Sec7 domain-containing protein ARFGEF, putative	50	iCn3D view
PF3D7_1443000	Q8IL41	serine/threonine protein kinase	18	iCn3D view
PF3D7_1443400	Q8IL37	WD repeat-containing protein	3	iCn3D view
PF3D7_1443600	Q8IL35	gamma-tubulin complex component, putative	12	iCn3D view
PF3D7_1443800	Q8IL33	pre-mRNA-splicing factor CWC24, putative	5	iCn3D view
PF3D7_1444100	Q8IL30	conserved Plasmodium protein, unknown function	4	iCn3D view
PF3D7_1444300	Q8IL28	1-acyl-sn-glycerol-3-phosphate acyltransferase, putative	1	iCn3D view
PF3D7_1444400	Q8IL27	conserved Plasmodium protein, unknown function	1	iCn3D view
PF3D7_1444500	Q8IL26	eukaryotic translation initiation factor 2-alpha kinase 1	3	iCn3D view
PF3D7_1444800	Q7KQL9	fructose-bisphosphate aldolase	6	iCn3D view
PF3D7_1445100	Q8IL22	histidine--tRNA ligase, putative	2	iCn3D view
PF3D7_1445200	Q8IL21	ATP-dependent RNA helicase MAK5, putative	2	iCn3D view
PF3D7_1445300	Q8IL20	ribosomal protein S29, mitochondrial, putative	1	iCn3D view
PF3D7_1445400	Q8IL19	protein serine/threonine kinase-1	30	iCn3D view
PF3D7_1445500	Q8IL18	conserved Plasmodium protein, unknown function	1	iCn3D view
PF3D7_1445600	Q8IL17	RNA-binding protein, putative	1	iCn3D view
PF3D7_1445700	Q8IL16	conserved Plasmodium protein, unknown function	21	iCn3D view
PF3D7_1445800	Q8IL15	conserved Plasmodium membrane protein, unknown function	3	iCn3D view
PF3D7_1445900	Q8IL13	ATP-dependent RNA helicase DDX17	7	iCn3D view
PF3D7_1446200	Q8IL11	M17 leucyl aminopeptidase	1	iCn3D view

PF3D7_1446600	Q8IL07	centrin-2	6	iCn3D view
PF3D7_1446700	Q8IL06	apicomplexan kinetochore protein 4, putative	3	iCn3D view
PF3D7_1446900	A0A144A3U7	glutaminyl-peptide cyclotransferase, putative	1	iCn3D view
PF3D7_1447000	Q8IL02	40S ribosomal protein S5	7	iCn3D view
PF3D7_1447100	Q8IL01	conserved Plasmodium protein, unknown function	1	iCn3D view
PF3D7_1447200	Q8IL00	conserved protein, unknown function	10	iCn3D view
PF3D7_1447900	Q8IKZ6	multidrug resistance protein 2	22	iCn3D view
PF3D7_1448000	Q8IKZ5	U3 small nucleolar RNA-associated protein 12, putative	5	iCn3D view
PF3D7_1448100	Q8IKZ4	conserved Plasmodium protein, unknown function	1	iCn3D view
PF3D7_1448200	Q8IKZ3	condensin-2 complex subunit G2, putative	1	iCn3D view
PF3D7_1448300	Q8IKZ0	conserved protein, unknown function	25	iCn3D view
PF3D7_1448600	Q8IKY7	SNARE protein, putative	4	iCn3D view
PF3D7_1448700	Q8IKY6	HSP20-like chaperone, putative	3	iCn3D view
PF3D7_1449000	Q8IKY4	gamete egress and sporozoite traversal protein, putative	1	iCn3D view
PF3D7_1449300	A0A144A465	transcription factor IIIb subunit, putative	1	iCn3D view
PF3D7_1449400	Q8IKY1	crossover junction endonuclease MUS81, putative	14	iCn3D view
PF3D7_1449500	Q8IKY0	AP2 domain transcription factor AP2-O5, putative	12	iCn3D view
PF3D7_1449600	Q8IKX9	conserved protein, unknown function	6	iCn3D view
PF3D7_1449700	Q8IKX8	exosome complex exonuclease RRP6	6	iCn3D view
PF3D7_1450000	Q8IKX5	serine/threonine protein kinase, putative	14	iCn3D view
PF3D7_1450100	Q8IKX4	signal recognition particle subunit SRP54	5	iCn3D view
PF3D7_1450400	Q8IKX2	E3 ubiquitin-protein ligase ZNF598, putative	14	iCn3D view
PF3D7_1450500	Q8IKX1	conserved Plasmodium protein, unknown function	8	iCn3D view
PF3D7_1450700	Q8IKW9	conserved Plasmodium protein, unknown function	4	iCn3D view
PF3D7_1451000	Q8IKW6	conserved Plasmodium protein, unknown function	6	iCn3D view

PF3D7_1451100	Q8IKW5	elongation factor 2	17	iCn3D view
PF3D7_1451300	Q8IKW3	E3 SUMO-protein ligase NSE2, putative	2	iCn3D view
PF3D7_1451400	Q8IKW2	transcriptional regulatory protein sir2b	5	iCn3D view
PF3D7_1451500	Q8IKW1	pre-mRNA-splicing factor CWF18, putative	1	iCn3D view
PF3D7_1451800	Q8IKV8	sortilin	6	iCn3D view
PF3D7_1451900	Q8IKV7	ribosome biogenesis protein TSR1, putative	3	iCn3D view
PF3D7_1452000	Q8IKV6	rhoptry neck protein 2	17	iCn3D view
PF3D7_1452500	Q8IKV1	syntaxin-6, putative	7	iCn3D view
PF3D7_1452600	Q8IKV0	conserved Plasmodium protein, unknown function	12	iCn3D view
PF3D7_1452700	Q8IKU9	U1 snRNP-associated protein, putative	8	iCn3D view
PF3D7_1453000	Q8IKU7	conserved Plasmodium protein, unknown function	8	iCn3D view
PF3D7_1453100	Q8IKU6	dynactin subunit 4, putative	1	iCn3D view
PF3D7_1453200	Q8IKU5	conserved Plasmodium protein, unknown function	14	iCn3D view
PF3D7_1453400	Q8IKU4	gamma-tubulin complex component, putative	4	iCn3D view
PF3D7_1453700	Q8IKU1	HSP90 co-chaperone p23	3	iCn3D view
PF3D7_1453900	Q8IKT9	conserved Plasmodium protein, unknown function	5	iCn3D view
PF3D7_1454000	Q8IKT8	RNA-binding protein, putative	5	iCn3D view
PF3D7_1454200	Q8IKT7	conserved Plasmodium protein, unknown function	4	iCn3D view
PF3D7_1454400	A0A144A2H0	aminopeptidase P	1	iCn3D view
PF3D7_1454700	Q8IKT2	6-phosphogluconate dehydrogenase, decarboxylating	1	iCn3D view
PF3D7_1455400	Q8IKS4	hemolysin III	1	iCn3D view
PF3D7_1455500	Q8IKS3	AP-1 complex subunit gamma, putative	5	iCn3D view
PF3D7_1455600	Q8IKS2	ferlin, putative	1	iCn3D view
PF3D7_1455700	Q8IKS1	conserved Plasmodium protein, unknown function	14	iCn3D view
PF3D7_1456200	Q8IKR7	conserved Plasmodium protein, unknown function	1	iCn3D view

PF3D7_1456300	Q8IKR6	conserved Plasmodium protein, unknown function	1	iCn3D view
PF3D7_1456400	Q8IKR5	conserved Plasmodium protein, unknown function	17	iCn3D view
PF3D7_1456500	Q8IKR4	STAG domain-containing protein, putative	34	iCn3D view
PF3D7_1456700	Q8IKR2	conserved Plasmodium protein, unknown function	4	iCn3D view
PF3D7_1456800	Q8IKR1	V-type H(+)-translocating pyrophosphatase, putative	1	iCn3D view
PF3D7_1457000	Q8IKQ9	signal peptide peptidase	2	iCn3D view
PF3D7_1457200	Q7KQL8	thioredoxin 1	2	iCn3D view
PF3D7_1457300	Q8IKQ7	MA3 domain-containing protein, putative	8	iCn3D view
PF3D7_1457400	Q8IKQ6	conserved Plasmodium protein, unknown function	7	iCn3D view
PF3D7_1457500	Q8IKQ5	vacuolar protein sorting-associated protein 4	3	iCn3D view
PF3D7_1457900	Q8IKQ1	conserved Plasmodium protein, unknown function	40	iCn3D view
PF3D7_1458000	Q8I6V0	cysteine proteinase falcipain 1	2	iCn3D view
PF3D7_1458300	Q8IKP8	alpha/beta hydrolase, putative	8	iCn3D view
PF3D7_1458400	Q8IKP7	aminodeoxychorismate lyase	1	iCn3D view
PF3D7_1458600	Q8IKP5	zinc finger protein, putative	5	iCn3D view
PF3D7_1458700	Q8IKP4	exonuclease V, mitochondrial, putative	1	iCn3D view
PF3D7_1458800	Q8IKP3	DNA-directed RNA polymerase III subunit RPC5, putative	1	iCn3D view
PF3D7_1459000	Q8IKP1	ATP-dependent RNA helicase DBP5	12	iCn3D view
PF3D7_1459200	Q8IKN9	WD repeat-containing protein, putative	1	iCn3D view
PF3D7_1459400	Q8IKN7	conserved protein, unknown function	2	iCn3D view
PF3D7_1459600	Q8IKN5	AP-4 complex accessory subunit Tepsin, putative	12	iCn3D view
PF3D7_1459700	Q8IKN4	pyridoxal 5'-phosphate synthase, putative	1	iCn3D view
PF3D7_1459900	Q8IKN2	rhoptry protein, putative	1	iCn3D view
PF3D7_1460100	Q8IKN0	FYVE and coiled-coil domain-containing protein	6	iCn3D view
PF3D7_1460400	Q8IKM8	ubiquitin carboxyl-terminal hydrolase isozyme L3	3	iCn3D view

PF3D7_1460600	Q8IKM6	inner membrane complex sub-compartment protein 3	3	iCn3D view
PF3D7_1460700	Q8IKM5	60S ribosomal protein L27	4	iCn3D view
PF3D7_1460800	Q8IKM4	snRNA-activating protein complex subunit 3, putative	1	iCn3D view
PF3D7_1461000	Q8IKM2	conserved Plasmodium protein, unknown function	2	iCn3D view
PF3D7_1461100	Q8IKM1	conserved Plasmodium protein, unknown function	7	iCn3D view
PF3D7_1461300	Q8IKL9	40S ribosomal protein S28e, putative	3	iCn3D view
PF3D7_1461400	Q8IKL8	MORN repeat protein, putative	7	iCn3D view
PF3D7_1461600	Q8IKL7	splicing factor 3B subunit 2, putative	11	iCn3D view
PF3D7_1461800	A0A144A533	conserved Plasmodium protein, unknown function	4	iCn3D view
PF3D7_1461900	Q8IKL5	valine--tRNA ligase, putative	2	iCn3D view
PF3D7_1462100	Q8IKL3	conserved Plasmodium protein, unknown function	2	iCn3D view
PF3D7_1462300	Q8IKL1	GTP-binding protein, putative	7	iCn3D view
PF3D7_1462500	Q8IKK9	conserved Plasmodium protein, unknown function	1	iCn3D view
PF3D7_1462800	Q8IKK7	glyceraldehyde-3-phosphate dehydrogenase	9	iCn3D view
PF3D7_1462900	Q8IKK6	AAA ATPase, putative	1	iCn3D view
PF3D7_1463200	Q8IKK4	replication factor C subunit 3, putative	2	iCn3D view
PF3D7_1463400	Q8IKK2	DNA-directed RNA polymerase III subunit RPC4, putative	2	iCn3D view
PF3D7_1463900	Q8IKJ8	rhoptry neck protein 11, putative	3	iCn3D view
PF3D7_1464000	Q8IKJ7	YL1 nuclear protein, putative	7	iCn3D view
PF3D7_1464200	Q8IKJ5	zinc finger CCCH domain-containing protein, putative	1	iCn3D view
PF3D7_1464600	Q8IKJ1	serine/threonine protein phosphatase UIS2, putative	6	iCn3D view
PF3D7_1464700	Q8IKJ0	ATP synthase (C/AC39) subunit, putative	4	iCn3D view
PF3D7_1465100	Q8IKI7	conserved oligomeric Golgi complex subunit 6, putative	4	iCn3D view
PF3D7_1465200	Q8IKI6	mediator of RNA polymerase II transcription subunit 4, putative	3	iCn3D view
PF3D7_1465300	Q8IKI5	ribonuclease Z, putative	1	iCn3D view

PF3D7_1465700	Q8IKI0	plasmepsin VIII, putative	1	iCn3D view
PF3D7_1465900	Q8IKH8	40S ribosomal protein S3	10	iCn3D view
PF3D7_1466100	Q8IKH5	protein phosphatase containing kelch-like domains	9	iCn3D view
PF3D7_1466300	Q8IKH3	26S proteasome regulatory subunit RPN2, putative	12	iCn3D view
PF3D7_1466400	Q8IKH2	AP2 domain transcription factor AP2-EXP	23	iCn3D view
PF3D7_1466600	Q8IKH1	enkurin domain-containing protein, putative	2	iCn3D view
PF3D7_1466800	Q8IKG9	NOC3 domain-containing protein, putative	15	iCn3D view
PF3D7_1466900	Q8IKG8	conserved Plasmodium protein, unknown function	25	iCn3D view
PF3D7_1467000	A0A144A2U1	magnesium transporter, putative	1	iCn3D view
PF3D7_1467100	Q8IKG6	DNA-3-methyladenine glycosylase	2	iCn3D view
PF3D7_1467500	Q8IKG2	DNA/RNA-binding protein KIN17, putative	4	iCn3D view
PF3D7_1467900	Q8IKF8	rab GTPase activator, putative	30	iCn3D view
PF3D7_1468200	Q8IKF5	telomere length and silencing protein 1, putative	3	iCn3D view
PF3D7_1468400	A0A144A3J8	C3H1-type zinc finger protein CZIF1	15	iCn3D view
PF3D7_1468700	Q8IKF0	eukaryotic initiation factor 4A	19	iCn3D view
PF3D7_1469100	Q8IKE6	Golgi SNAP receptor complex member 1, putative	3	iCn3D view
PF3D7_1469200	Q8IKE5	shewanella-like protein phosphatase 1, putative	1	iCn3D view
PF3D7_1469300	Q8IKE4	pre-rRNA-processing protein PNO1, putative	2	iCn3D view
PF3D7_1469600	Q8IKE1	acetyl-CoA carboxylase	1	iCn3D view
PF3D7_1469800	Q8IKD9	mediator of RNA polymerase II transcription subunit 22, putative	5	iCn3D view
PF3D7_1470100	Q8IKD7	conserved Plasmodium protein, unknown function	5	iCn3D view
PF3D7_1470200	Q8IKD6	conserved Plasmodium protein, unknown function	1	iCn3D view
PF3D7_1470300	Q8IKD5	conserved Plasmodium protein, unknown function	1	iCn3D view
PF3D7_1470500	Q8IKD3	cGMP-specific 3',5'-cyclic phosphodiesterase delta	2	iCn3D view
PF3D7_1471100	Q8IKC8	exported protein 2	6	iCn3D view

PF3D7_1471200	Q8IKC7	inorganic anion exchanger, inorganic anion antiporter	6	iCn3D view
PF3D7_1471400	Q8IKC5	diacylglycerol kinase, putative	8	iCn3D view
PF3D7_1471600	Q8IKC3	apical polar ring protein APR2, putative	2	iCn3D view
PF3D7_1472200	A0A144A4T0	histone deacetylase, putative	24	iCn3D view
PF3D7_1472300	Q8IKB5	conserved protein, unknown function	1	iCn3D view
PF3D7_1472400	Q8IKB4	M1-family alanyl aminopeptidase, putative	1	iCn3D view
PF3D7_1472500	Q8IKB3	conserved Plasmodium protein, unknown function	1	iCn3D view
PF3D7_1472900	Q8IKA9	dihydroorotase, putative	2	iCn3D view
PF3D7_1473100	Q8IKA7	GTPase-activating protein, putative	1	iCn3D view
PF3D7_1473200	Q8IKA6	DnaJ protein, putative	29	iCn3D view
PF3D7_1473400	Q8IKA3	conserved protein, unknown function	4	iCn3D view
PF3D7_1473500	Q8IKA2	conserved Plasmodium protein, unknown function	1	iCn3D view
PF3D7_1473700	Q8IKA0	nucleoporin NUP116/NSP116, putative	24	iCn3D view
PF3D7_1473900	Q8IK99	ribosome assembly 1 protein	1	iCn3D view
PF3D7_1474000	Q8IK98	conserved Plasmodium protein, unknown function	5	iCn3D view
PF3D7_1474300	Q8IK95	DNA repair metallo-beta-lactamase protein, putative	2	iCn3D view
PF3D7_1474400	Q8IK94	conserved Plasmodium protein, unknown function	4	iCn3D view
PF3D7_1474500	Q8IK93	splicing factor 3A subunit 1, putative	9	iCn3D view
PF3D7_1474600	Q8IK92	vacuole membrane protein 1, putative	2	iCn3D view
PF3D7_1474800	Q8IK90	proteasome subunit alpha type-1, putative	5	iCn3D view
PF3D7_1474900	Q8IK89	trailer hitch homolog, putative	17	iCn3D view
PF3D7_1475200	Q8IK86	conserved protein, unknown function	2	iCn3D view
PF3D7_1475600	Q8IK82	bromodomain protein 4, putative	4	iCn3D view
PF3D7_1475700	Q8IK81	tubulin epsilon chain, putative	1	iCn3D view
PF3D7_1475900	A0A5K1K8Y2	KELT protein	1	iCn3D view

PF3D7_1476500	Q8IK71	probable protein, unknown function	1	iCn3D view
PF3D7_1476600	Q8IK70	Plasmodium exported protein, unknown function	2	iCn3D view
PF3D7_1477300	Q8IK62	Plasmodium exported protein (PHIST), unknown function	2	iCn3D view
PF3D7_1477400	Q8IK61	Plasmodium exported protein (PHIST), unknown function	1	iCn3D view
PF3D7_1477500	Q8IK60	Plasmodium exported protein (PHISTb), unknown function	1	iCn3D view
PF3D7_1478000	Q8IK55	Plasmodium exported protein (PHISTa), unknown function	5	iCn3D view
PF3D7_1478600	Q8IK49	EMP1-trafficking protein	10	iCn3D view
PF3D7_1479000	Q7KQL7	acyl-CoA synthetase	1	iCn3D view
PF3D7_0310200	O77336	phd finger protein, putative	11	iCn3D view
PF3D7_0511500	Q8I3Z1	RNA pseudouridylate synthase, putative	77	iCn3D view
PF3D7_1021700	Q8IJI6	VPS13 domain-containing protein, putative	97	iCn3D view
PF3D7_1426800	Q8ILJ6	conserved Plasmodium protein, unknown function	12	iCn3D view
